# Mechanistic investigations into the cyclization and crystallization of benzobisoxazole-linked two-dimensional covalent organic frameworks†
†Electronic supplementary information (ESI) available: Synthetic procedures, FT-IR, solid-state ^13^C NMR, TGA, PXRD, SEM, optimized *XYZ* coordinates, vibrational frequencies, and full NPA results. See DOI: 10.1039/c8sc01683f


**DOI:** 10.1039/c8sc01683f

**Published:** 2018-06-25

**Authors:** David A. Pyles, William H. Coldren, Grace M. Eder, Christopher M. Hadad, Psaras L. McGrier

**Affiliations:** a Department of Chemistry & Biochemistry , The Ohio State University , Columbus , Ohio 43210 , USA

## Abstract

Although many diverse covalent organic frameworks (COFs) have been synthesised over the past decade, our fundamental understanding of their nucleation and growth during the crystallization process has progressed slowly for many systems. In this work, we report the first in-depth mechanistic investigation detailing the role of nucleophilic catalysts during the formation of two distinct benzobisoxazole (BBO)-linked COFs. The BBO-COFs were constructed by reacting 1,3,5-tris(4-formylphenyl)benzene (TFPB) and 1,3,5-tris(4-formylphenyl)triazine (TFPT) *C*_3_-symmetric monomers with a *C*_2_-symmetric *o*-aminophenol substituted precursor using different nucleophiles (*e.g.* NaCN, NaN_3_, and NaSCH_3_). Our experimental and computational results demonstrate that the nucleophiles help initiate an oxidative dehydrogenation pathway by producing radical intermediates that are stabilized by a captodative effect. We also demonstrate that the electron deficient TFPT monomer not only aids in enhancing the crystallinity of the BBO-COFs but also participates in the delocalization of the radicals generated to help stabilize the intermediates.

## Abstract 2

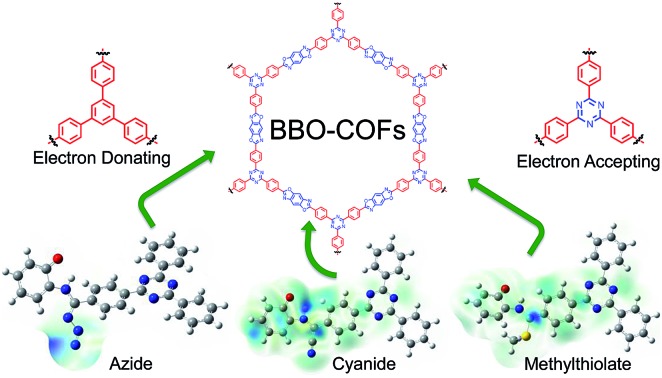
A mechanistic investigation detailing the role of nucleophilic catalysts during the formation of benzobisoxazole (BBO)-linked COFs.

## Introduction

Covalent organic frameworks (COFs)^1^–^4^ are an advanced class of crystalline porous materials that have emerged as appealing candidates for applications related to gas storage,^5^,^6^ catalysis,^7^–^9^ optoelectronics,^10^–^14^ proton conduction,^15^,^16^ and energy storage.^17^–^19^ COFs offer the unique advantage of utilizing the geometry of various molecular building blocks to construct atomically precise two-dimensional (2D) and three-dimensional (3D) polymeric networks with high surface areas and tunable pore sizes. While the field has enjoyed a rapid increase of exceptional COF architectures with tailored functionalities over the past decade, there has been an increased effort of late to probe the underlying mechanisms that govern the nucleation and crystallization of COFs containing the most commonly used boronate ester^20^–^23^ and imine^24^–^26^ covalent linkages. Such investigations are believed to be vital for not only bypassing the tedious task of screening numerous reactions conditions (*e.g.*, solvent concentrations, reaction times, temperature, *etc.*) to produce a desired framework, but also establishing a rational experimental protocol for generating high quality COF structures with improved efficiency and controlled morphologies.

**Scheme 1:**
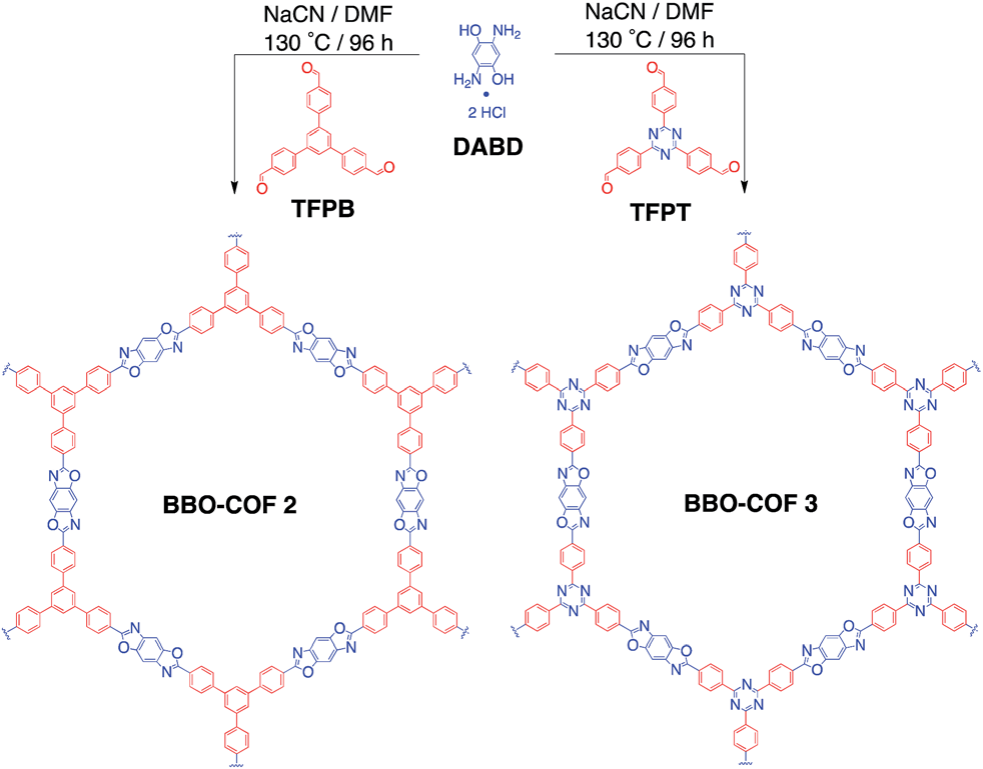
Cyanide-catalyzed synthesis of BBO-COF **2** and **3**.

Recently, we reported a cyanide-catalyzed protocol to synthesize chemically stable benzobisoxazole-linked (BBO) COFs utilizing *C*_3_-symmetric formyl- and *C*_2_-symmetric *o*-aminophenol-substituted organic linkers.^26^ Upon doing so, we discovered that the addition of cyanide enables the construction of ordered polymeric materials with high surface areas. We hypothesized that the addition of cyanide induces ring closure and the formation of a benzoxazoline intermediate that then undergoes subsequent aerobic oxidation in the presence of air to form the BBO-COF. This result is consistent with what has been reported for the cyanide-catalyzed 5-exo-tet cyclization of benzo-fused azoles,^28^,^29^ which is based on Baldwin's rules^30^ for ring closure. We concluded that the non-covalent interactions between the adjacent π-systems during this cyclization process were significant enough to stabilize the stacking layers upon formation of the irreversible BBO-linkage giving rise to the ordered 2D BBO-COF structures. In contrast, there are only a few synthetic routes that have been reported to construct BBO-based porous materials: (1) the annealing of silyl protected prepolymers at high temperatures,^31^ and (2) the direct formation of aniline Schiff base precursors followed by subsequent dehydrogenation and cyclization.^32^ While both methods are effective, they exclusively lead to the formation of amorphous polymer networks. Since the second synthetic route is similar to our method with the exception of adding a nucleophile to catalyze the reaction, we began to question the active role of cyanide during the cyclization process. Since BBO-COFs could be utilized for developing 2D materials for organic electronics^33^ and sensory applications,^34^ a thorough investigation into the optimal reaction conditions needed to produce ordered BBO-based polymeric systems are warranted (Scheme 1).

Recently, Wang and Yu reported a density functional theory (DFT)^35^ study suggesting that cyanide does not actually promote the direct cyclization of benzoxazoles but, instead, assists with the oxidative dehydrogenation process by generating a radical intermediate that is stabilized through a captodative effect.^36^ The captodative effect is a process by which adjacent electron donating and electron withdrawing substituents are utilized to stabilize a radical center through resonance stabilization. This concept of push–pull resonance stabilization was embraced in the field of polymer science as a method to help improve the lack of control and reactivity of 1,1-disubstituted monomers used in radical polymerizations.^37^ It has also emerged as an alternative method for enhancing the conductivity of organic electronic devices.^38^ However, mechanistic studies detailing how the captodative effect and careful selection of organic linkers can support the nucleation and growth of ordered 2D polymeric systems has yet to be reported.

In an effort to validate the likelihood of a mechanistic pathway involving the captodative effect experimentally, we were curious to determine if other nucleophiles (*e.g.* NaN_3_, NaSCH_3_) could be used to catalyze the formation of the BBO-COFs. We were also interested in examining what impact an electron deficient organic linker like 1,3,5-tris(4-formylphenyl)triazine (TFPT) would have on the formation and crystallization of the BBO-linked COFs. Since triazine-based monomers are known for exhibiting high electron mobilities (thanks to their electron withdrawing nature),^39^ we hypothesized that this feature could assist with stabilizing the radical species generated during the oxidative dehydrogenation process.

In this study, we first established the optimal cyanide-catalyzed reaction conditions for synthesizing BBO-COF **3** using TFPT and 2,5-diamino-1,4-benzenediol dihydrochloride (DABD) organic linkers.^40^ Afterwards, we investigated the ability of other good nucleophiles like NaN_3_ and NaSCH_3_ to initiate the formation of BBO-COF **3** and the previously synthesized BBO-COF **2**. These studies were performed in conjunction with one another to determine if the electron withdrawing TFPT linker of BBO-COF **3** would exhibit any enhanced effect on the formation and crystallization of the COFs in comparison to the isostructural 1,3,5-tris(4-formylphenyl)benzene (TFPB) linker that was used to synthesize BBO-COF **2**. We demonstrate that NaN_3_ and NaSCH_3_ are effective at catalyzing the formation of BBO-COFs, but NaCN is the only catalyst that promotes the stabilization of radical intermediates through the captodative effect. Interestingly, we also show that the electron withdrawing TFPT monomer not only enhances the crystallinity of BBO-COF **3**, but also plays a significant role in stabilizing the radical intermediates generated during oxidative dehydrogenation. These results were validated using DFT calculations, including population and spin density analyses, along with powder X-ray diffraction (PXRD). We expect that this work will provide a rational protocol for constructing highly ordered BBO-based polymeric systems for practical applications.

## Results and discussion

### Synthesis and characterization of BBO-COF **3**

BBO-COF **3** was synthesized by reacting TFPT with DABD in DMF at –15 °C for ∼3 h. Afterwards, the reaction mixture was slowly warmed to room temperature overnight before adding 1 equiv. of NaCN dissolved in 0.2 mL of methanol. The mixture was then stirred at 130 °C for four days in the presence of air. The product was obtained by filtration and washed with acetone to afford a light brown crystalline solid. BBO-COF **3** was purified by immersing the solids in methanol and acetone for 24 h to remove any unreacted monomers and dried under vacuum. Thermogravimetric analysis (TGA) revealed that BBO-COF **3** maintained more than 98% of its weight up to 470 °C (Fig. S32†).

BBO-COF **3** was characterized using Fourier transform infrared (FT-IR) and ^13^C cross-polarization magic angle spinning (CP-MAS) spectroscopic analyses. The FT-IR spectrum revealed stretching modes at 1662 (C=N) and 1120 cm^–1^ (C–O) confirming the formation of the benzoxazole ring (Fig. S11†). The TFPT linker exhibited two intense stretches at 1515 and 1360 cm^–1^, which correspond to the presence of the benzene and triazine moieties (Fig. S10†). The triazine ring and BBO-linkage were also confirmed by solid-state ^13^C CP-MAS NMR, displaying distinct resonances at 167.7, 160.5, and 146.4 ppm (Fig. S31†).

**Fig. 1:**
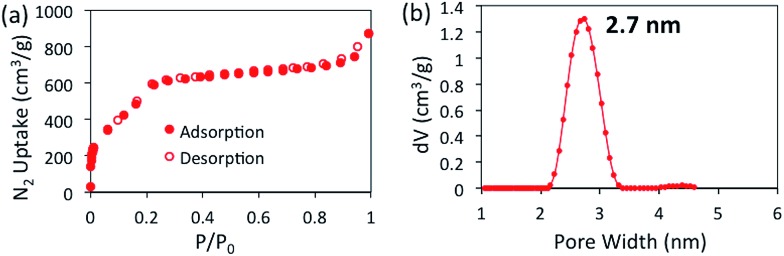
Nitrogen isotherm at 77 K (a) and NLDFT pore size distribution (b) for BBO-COF **3**.

The porosity of BBO-COF **3** was evaluated using nitrogen gas adsorption measurements at 77 K (Fig. 1).

BBO-COF **3** displays a type IV isotherm exhibiting a steep uptake at low pressure (*P*/*P*_0_ < 0.06) followed by a noticeable step between *P*/*P*_0_ = 0.06 and 0.22 which validates the mesoporosity of the material. The Brunauer–Emmett–Teller (BET) method was applied over the low-pressure region (0.01 < *P*/*P*_0_ < 0.22) of the isotherm to afford a surface area of 2039 m^2^ g^–1^. It is worth noting that this is the highest surface area reported to date for a benzoxazole-linked porous material.^27^,^31^,^32^ The total pore volume of BBO-COF **3** calculated at *P*/*P*_0_ = 0.989 provided a value of 1.22 cm^3^ g^–1^. The pore size distribution was estimated using nonlocal density functional theory (NLDFT) to provide an average pore size of 2.7 nm, which is close to the predicted value of 3.3 nm. In comparison, the isostructural BBO-COF **2** exhibited an average pore size of 1.8 nm. The enhanced pore size of BBO-COF **3** could be attributed to the planarity of the triazine TFPT, which allows for coplanarity between the vertex and linker units (Fig. S46 and S47†). This is in contrast to the out-of-plane twisting of the TFPB moieties, which can hinder the formation of vertically stacked eclipsed layers.

In contrast to the PXRD pattern of BBO-COF **2**, BBO-COF **3** displays an enhancement in crystallinity (see Fig. S30†). We believe this enhancement is attributed to (1) the planarity of the TFPT units allowing for more efficient van der Waals interactions between the layers, and (2) the ability of the electron deficient triazine units to form donor–acceptor (DA) stacks with the adjacent TFPT aromatic rings along the *c* direction. Lotsch and coworkers have also observed this phenomenon for imine-linked COFs containing triazine moieties.^41^ Since DA interactions are often more favourable than non-covalent interactions between π-electron rich aromatic rings,^42^ this could also further explain why the offsets for BBO-COF **2** (15 Å) are more significant compared to BBO-COF **3** (6 Å).

### BBO-COF **3** time-dependent growth studies

Before evaluating other nucleophiles as potential catalysts, we first wanted to ensure that the optimal cyanide-catalyzed reaction conditions for constructing BBO-COF **3** were established. Initially, we found that allowing the monomers to react at –15 °C under nitrogen for several hours before adding 1 eq. of cyanide and heating the reaction to 130 °C under air facilitated the precipitation of amorphous imine-linked porous polymers after reacting for one day. However, it was unclear if the reaction really required four days to produce the BBO-COFs.

**Fig. 2:**
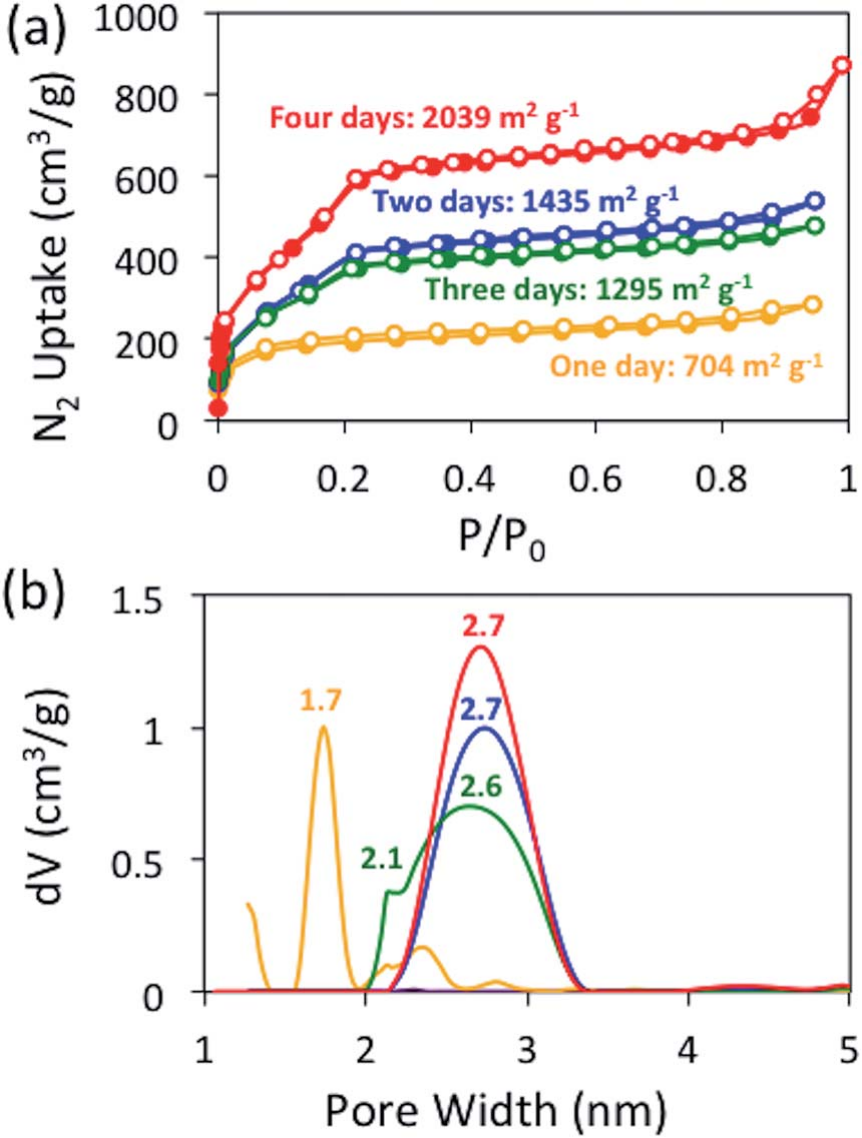
Nitrogen isotherms at 77 K (a) and NLDFT (b) pore size distributions for BBO-COF **3** over a four-day reaction period.

**Fig. 3:**
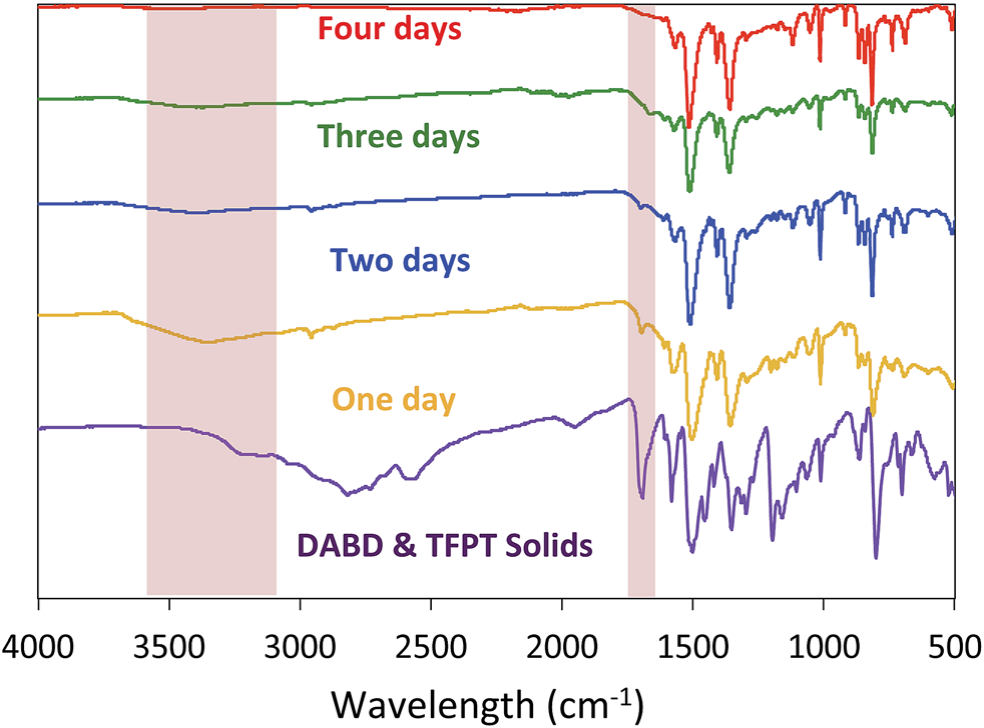
IR spectra of BBO-COF **3** highlighting the disappearance of the OH stretch from phenolic imine-linked intermediate and aldehyde stretch from the TFPT linker over a four-day reaction period.

To address this issue, we monitored the growth of BBO-COF **3** over time. After reacting for one day, the isolated solid provided a low surface area of 704 m^2^ g^–1^ and a pore size of 1.7 nm indicating that the reaction requires a longer reaction time (Fig. 2). The IR spectrum displayed a broad stretch at 3343 cm^–1^ and a sharp stretch at 1693 cm^–1^, which are attributed to the OH stretch from the phenolic imine-linked intermediate and the aldehyde (C=O) stretch from the TFPT linker, respectively (Fig. 3). Although the IR stretches for the intermediate and starting material begin to disappear after reacting for a few additional days, the surface area increases by two orders of magnitude to 1435 m^2^ g^–1^ after day two, but then surprisingly decreases to 1295 m^2^ g^–1^ on day three. This trend is echoed in the pore size data yielding a value of 2.7 nm on day two followed by the formation of multiple smaller pores at 2.6 and 2.1 nm on day three. Since the benzoxazole bond is irreversible, we believe that the dramatic changes in the surface area and pore size when transitioning from day-one to day-three could be attributed to the formation of phenolic imine-linked polymeric networks that are generated during the dynamic imine exchange process prior to the formation of the BBO linkage.^25^ PXRD patterns from the isolated solids also indicate that a four-day reaction period is required to attain the crystalline BBO-COF **3** structure (Fig. S20†). Allowing the reaction to proceed beyond the four-day reaction period did not lead to an additional increase in the surface area (Fig. S21†).

It is also worth noting that the BBO-linkage does not form without oxygen even under the optimal growth conditions (Fig. S6–S8 and S23–S25†).

### BBO-COF formation with different nucleophiles

**Scheme 2:**
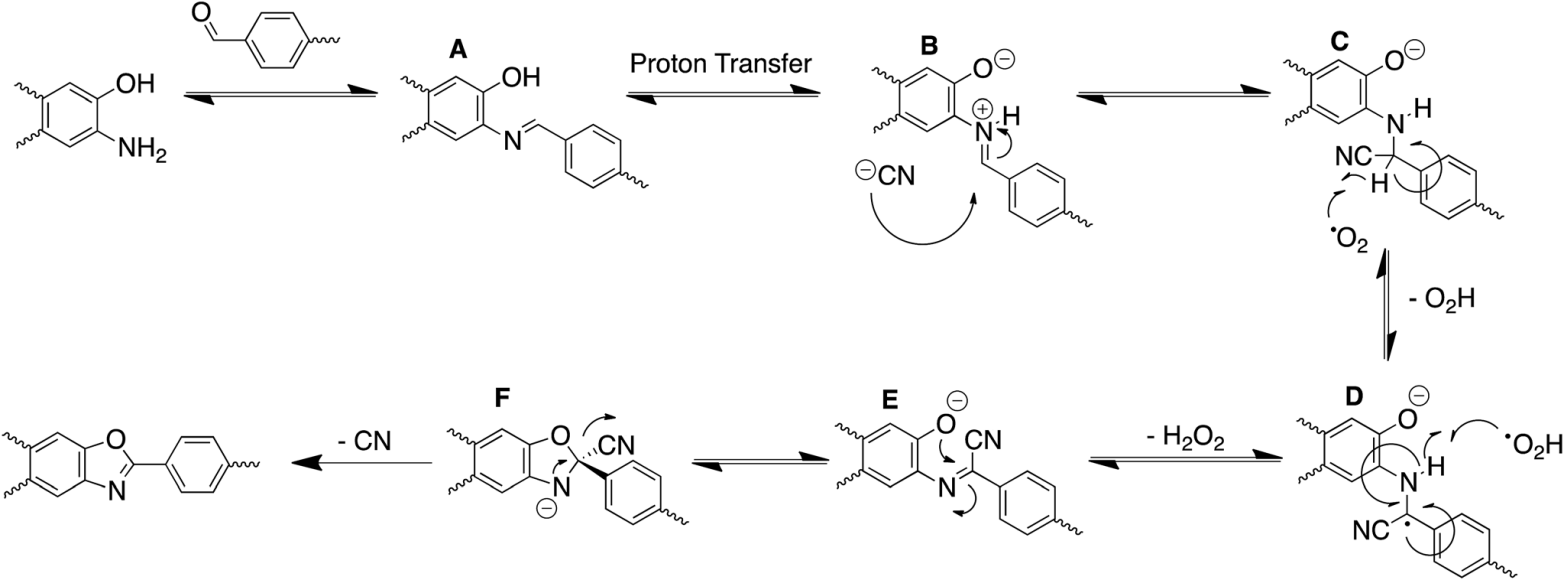
Proposed mechanism for the stepwise oxidative dehydrogenation pathway to form the BBO-linkage using NaCN as a catalyst.

With the optimal reaction conditions established, we examined if other nucleophiles could be utilized to catalyze the formation of the BBO-COFs. Wang and Yu have shown that the activation energy for the direct 5-exo-tet cyclization of benzoxazole in the presence of NaCN has to overcome a high-energy barrier of 47.9 kcal mol^–1^,^35^ and suggested that the cyclization proceeds through a stepwise mechanism. DFT calculations revealed that after the cyanide attacks the imine carbon to convert the hybridization from sp^2^ to sp^3^ triplet- state oxygen then directly abstracts a hydrogen from the newly generated sp^3^ α-carbon to produce a radical that is stabilized through the captodative effect (Scheme 2). Surprisingly, the activation energy for this process was found to be ∼24.4 kcal mol^–1^ lower than the suggested direct cyanide-catalyzed cyclization pathway. Intrigued by these results, we were curious to examine if (1) BBO-COF **2** and **3** could be constructed using other good nucleophiles like NaN_3_ and NaSCH_3_, and (2) the electron deficient TFPT linker of BBO-COF **3** would have any effect on the cyclization and crystallization of the framework compared to BBO-COF **2**, which contained the electron rich TFPB linker.

**Fig. 4:**
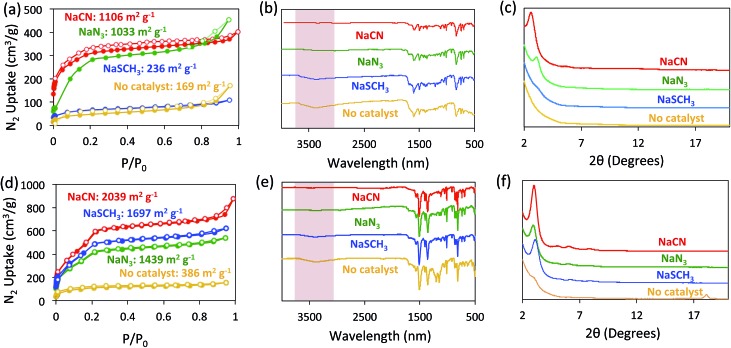
Nitrogen gas isotherms (a and d), IR spectra (b and e), and PXRD patterns (c and f) of BBO-COF **2** (top) and BBO-COF **3** (bottom). Each reaction was catalyzed by adding 1 eq. of NaCN (red), NaSCH_3_ (blue), and NaN_3_ (green). Reactions in which no catalyst was used are shown in orange.

The formation of BBO-COF **2** and **3** using NaN_3_ and NaSCH_3_ as catalysts was evaluated using nitrogen gas adsorption isotherms, IR spectroscopy, and PXRD analysis (Fig. 4). Since the direct cyclization of benzoxazoles only requires ∼8.4 kcal mol^–1^,^35^ we also monitored the formation of the BBO-COFs without the addition of a nucleophile. The activated solids for BBO-COF **2** provided surface area values of 1033 and 236 m^2^ g^–1^ for NaN_3_ and NaSCH_3_, respectively, whereas the reaction without catalyst yielded a surface area of 169 m^2^ g^–1^. Interestingly, NaSCH_3_ and NaN_3_ yielded pore sizes that were closer to the predicted value than NaCN (Fig. S5†). The IR spectra for BBO-COF **2** revealed that the reaction with NaSCH_3_ exhibited broad OH stretches at ∼3300 cm^–1^ indicating that the isolated solids still contain some of the unreacted phenolic imine-linked intermediate (Fig. 4b). The PXRD data revealed that NaN_3_ and NaCN were the only catalysts that produced crystalline samples of BBO-COF **2** while NaSCH_3_ afforded an amorphous porous polymer (Fig. 4c). In contrast to BBO-COF **2**, the activated solids for BBO-COF **3** yielded surface areas of 1697, 1439, and 386 m^2^ g^–1^ for NaSCH_3_, NaN_3_, and the reaction with no catalyst, respectively (Fig. 4d). The BBO-COF **3** synthesized without a catalyst contained two pores at 1.7 and 2.6 nm, while the others displayed one distinct pore size at ∼2.7 nm (Fig. S15†). NaSCH_3_ is the only nucleophile that exhibited an OH stretch at ∼3300 cm^–1^ signifying that a small portion of the polymer was not fully converted to the BBO-linkage (Fig. 4e). Surprisingly, all of the nucleophiles studied provided high quality samples of BBO-COF **3** with the lone exception being the reaction in which no catalyst was used (Fig. 4f). Although the complete formation of BBO-COF **2** and **3** seemed to vary upon the usage of NaSCH_3_ or NaN_3_, our studies suggest that NaCN is the most effective catalyst at producing crystalline BBO-COF materials. It should be noted that the reactions without catalyst generated amorphous porous polymers, and were unsuccessful at promoting the cyclization of the BBO-linkage even in the presence of oxygen. This also indicates that nucleophiles are critical for providing the sp^3^ hybridized α-carbon needed to initiate the stepwise oxidative dehydrogenation mechanistic pathway (Scheme 2).

### NPA population and spin density analysis – proposed mechanism

With this in mind, we wanted to validate that the radical generated at the α-carbon prior to the cyclization of the BBO-linkage is stabilized through the captodative effect. We were also curious to explore the role of the TFPB and TFPT linkers during the radical delocalization process. To examine this, we performed DFT geometry optimizations (B3LYP/6-31+G*)^43^–^46^ along with natural population analysis (NPA, B3LYP/6-31+G*)^47^ charge density (population) and spin density calculations on the fragment cores of BBO-COF **2** and **3**, as implemented in Gaussian 16.^48^ The results of these calculations are shown in Tables 1 and 2, respectively. Surprisingly, the calculations show that 100% of the single electron is delocalized entirely on the azide for both BBO-COF **2** and **3** indicating that these intermediates are not stabilized by the captodative effect. In the case of cyanide, the single electron for BBO-COF **2** is broadly delocalized on the nitrogen atom (25%), cyanide substituent (10%), and the TFPB linker (18%). In contrast, BBO-COF **3** displays similar percentages for the nitrogen atom and cyanide substituent, but exhibits a 7% increase in charge delocalization for the electron deficient TFPT linker. The calculations for cyanide confirm that (1) the radical intermediates generated are stabilized by the captodative effect for these particular systems, and (2) electron deficient substituents can also play a vital role in stabilizing radical intermediates that are generated during the formation of BBO-COFs. Lastly, only a small percentage of the single electron is delocalized on the SCH_3_ substituent for BBO-COF **2** and **3** possibly due to its electron donating nature, while 32% and 47% are delocalized on the TFPB and TFPT linkers, respectively. The higher charge delocalization for the latter could explain why the NaSCH_3_ catalyzed reaction for BBO-COF **3** produces a crystalline solid, while the reaction for BBO-COF **2** yields an amorphous powder.

**Table 1:** B3LYP/6-31+G* NPA population and spin density analysis for BBO-COF **2**. The calculated structure with a single electron on the α-carbon is shown

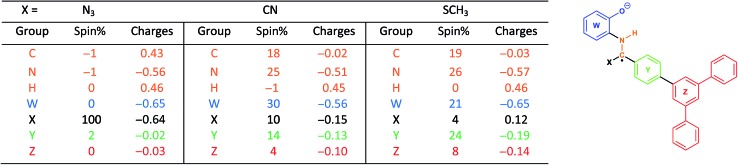

**Table 2:** B3LYP/6-31+G* NPA population and spin density analysis for BBO-COF **3**. The calculated structure with a single electron on the α-carbon is shown

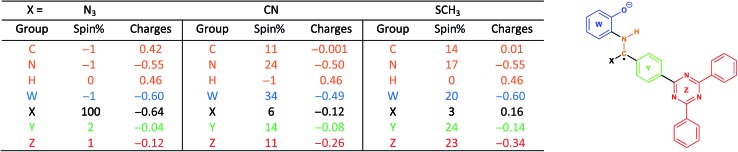

Based on the experimental and computational data collected, we propose the following mechanism for the cyclization of the BBO-linkage during the nucleation process (Scheme 2). The aminophenol and formyl precursors initially undergo an imine condensation reaction to produce (A) followed by subsequent proton transfer to form the protonated imine intermediate (B). Then, cyanide attacks the imine α-carbon of (B) to generate the sp^3^ hybridized intermediate (C). Afterwards, triplet state oxygen moves in to abstract the hydrogen atom at the sp^3^ hybridized α-carbon of (C) to produce intermediate (D) and generate a hydroperoxyl radical species. Later, this hydroperoxyl radical species returns to abstract a hydrogen atom from the β-nitrogen of (D) to eliminate hydrogen peroxide and produce intermediate (E). From here, an intramolecular cyclization followed by elimination of the cyano group produces the BBO-linkage. Although the proposed mechanism does shed more light on the role of cyanide during the cyclization process, it is unclear if radical–radical interactions^49^ between the adjacent layers of BBO-COFs occur or assist with the nucleation and growth process.

## Conclusions

In summary, we have reported the first in-depth mechanistic investigation into the cyclization and crystallization of BBO-COFs utilizing different nucleophiles (*e.g.* NaN_3_ and NaSCH_3_). However, we found that NaCN provided BBO-COFs with the highest surface areas and best crystallinity. Our experimental and electron spin population studies also confirm that the nucleophiles do not promote the direct cyclization of the BBO-linkage but instead, initiates an oxidative dehydrogenation mechanistic pathway by producing radical intermediates that are stabilized by a captodative effect. In addition, we discovered that the electron deficient TFPT monomer not only aids in increasing crystallinity of the materials by promoting the formation of D–A stacking layers, but also participates in the delocalization of radicals generated during the oxidative dehydrogenation process. We believe this study provides a fundamental understanding of how BBO-COFs are formed, and an intricate thread towards unravelling the mysteries surrounding the crystallization of COFs. Such investigations are necessary for obtaining high quality COF structures for practical applications.

## Conflicts of interest

There are no conflicts of interest.

## Supplementary Material

Supplementary information
