# Supplementary material for: Mechanistic investigations into the cyclization and crystallization of benzobisoxazole-linked two-dimensional covalent organic frameworks
Source: Chem Sci. 2018 Jun 25;9(30):6417–23. doi: 10.1039/c8sc01683f (PMC6115699; doi:10.1039/c8sc01683f)
Supplement: Supplementary file 1 [file SC-009-C8SC01683F-s001.pdf]

Electronic Supplementary Material (ESI) for Chemical Science.
This journal is © The Royal Society of Chemistry 2018

Supporting Information for

Mechanistic investigations into the cyclization and crystallization of benzobisoxazole-linked two-dimensional covalent organic frameworks

David A. Pyles, William H. Coldren, Grace, M. Eder, Christopher, M. Hadad, Psaras L. McGrier

Department of Chemistry & Biochemistry, The Ohio State University, Columbus, Ohio, 43210

TABLE OF CONTENTS

**A.**	**Materials**	**S2**
**B.**	**Instrumentation and Methods**	**S2**
**C.**	**Synthetic Methods**	**S3**
**D.**	**BBO Analog Trials**	**S4**
**E.**	**BBO-COF 2 Formation Studies**	**S5**
**F.**	**BBO-COF 3 Formation Studies**	**S12**
**G.**	**BBO-COF 3 Experimental & Simulated PXRD Data**	**S27**
**H.**	**Solid-State NMR Spectra**	**S31**
**I.**	**TGA Profile**	**S31**
**J.**	**Surface Area Analysis**	**S32**
**K.**	**SEM Images**	**S38**
**L.**	**Computational Studies**	**S38**

A. Materials

Unless stated otherwise all reagents were purchased from commercial sources and used without further purification. Dimethylformamide and tetrahydrofuran were purified by passage over activated alumina.

B. Instrumentation and Methods

Infrared spectra were recorded on a Thermo Scientific Nicolet iS5 with an iD7 diamond ATR attachment and are uncorrected.

Powder X-Ray diffraction (PXRD) patterns were recorded on a Bruker D8 Advance diffractometer (40 kV, 40 mA, sealed Cu X-Ray tube) equipped with an incident beam monochromator (Johansson type SiO_2-crystal) and Lynxeye XE-T position sensitive detector. Samples were mounted on a zero background sample holder by dropping powders from a vial and flattening them by firmly pressing the sample with a wide-blade spatula. No sample grinding was used prior to analysis. The holder was then placed on the mounting apparatus.

Surface area measurements were conducted on a Micromeritics ASAP 2020 Surface Area and Porosity Analyzer and a Micromeritics Tristar Surface Area and Porosity Analyzer using ca. 20 mg samples. Nitrogen isotherms were generated by incremental exposure to ultra high purity nitrogen up to ca. 1 atm in a liquid nitrogen (77 K) bath. Surface parameters were determined using BET adsorption models in the instrument software. Pore size distributions were determined using the non-local density functional theory (NLDFT) model (cylinder pore, N_2-cylindrical pores-oxide surface with high regularization) in the instrument software (Micromeritics ASAP 2020 V4.02).

^{1}H NMR spectra were recorded in deuterated solvents on a Bruker Avance DPX 400 (400 MHz). Chemical shits are reported in parts per million (ppm, δ) using the solvent as the internal standard. ^{13}C NMR spectra were recorded on a Bruker Avance DPX 400 (100 MHz) using the solvent as an internal standard.

Solid-state ^{13}C NMR spectra for BBO-COF 3 was recorded using a Bruker AVIII 600 MHz spectrometer with wide-bore magnet (600.3 MHz) using a 3.2 mm magic angle spinning (MAS) HXY solid-state NMR probe and running 32 k scans. Cross-polarization with MAS (CP-MAS) was used to acquire ^{13}C data at 150.9 MHz. The ^{13}C cross polarization time was 2 ms at 50 kHz for ^{13}C. ^{1}H decoupling was applied during data acquisition. The decoupling power corresponded to 100 kHz. The HXY sample spinning rate was 15 kHz.

Elemental analysis was performed by Galbraith Laboratories using a Thermo Finnigan FlashEA 1112 Elemental Analyzer.

Thermogravimetric analysis (TGA) was performed by Galbraith Laboratories by heating samples from 25 °C to 500 °C under air at a heating rate of 10 °C min^{-1}.

Scanning electron microscopy (SEM) was performed on a FEI sirion FE-SEM. Materials were deposited onto a film of a wet colloidal silver paint on an aluminum sample stub and dried in a vacuum oven at 40 °C. The samples were coated with gold in a Leica EM ACE600 coater, using rotation, to a depth of approximately 20 nm. After coating, the samples were imaged in the SEM at 5 keV, without tilting, using both the secondary electron (SE) detector and the through lens detector (TLD).

C. Synthetic Methods

2,5-Diamino-1,4-benzenediol dihydrochloride (DABD): this compound was prepared using procedures adapted from Strom and Jeffries-EL et al.[1,2]

1,3,5-Tris(4-formylphenyl)benzene (TFPB): this compound was prepared using a procedure from Cooper et al.[3]

1,3,5-Tris(4-bromophenyl)triazine: this compound was prepared using a procedure from Adachi et al.[4]

1,3,5-Tris(4-formylphenyl)triazine (TFPT): this compound was prepared using a procedure from El-Kaderi et al.[5]

BBO-COF 2: BBO-COF 2 was synthesized as previously reported, varying the nucleophilic catalyst's identity.[6]

BBO-COF 3: A dry 25 mL vial with stirbar was loaded with 2,5-diamino-1,4-benzenediol dihydrochloride (31.9 mg, 0.15 mmol, 1.5 eq) and DMF (5 mL) under a N_2 atmosphere. The solution was cooled to -15˚C (ethylene glycol/CO_2) and 2,4,6-Tris(4-formylphenyl)-1,3,5-triazine (39.3 mg, 0.10 mmol, 1 eq.) in DMF (5 mL) was added to the solution over 5 minutes. The solution was stirred at -15˚C for an additional 3 h before warming to room temperature in a water bath overnight. The reaction was opened to air and the NaCN (0.10 mmol, 1 eq) was dissolved in MeOH (0.2 mL) before adding to the solution. The reaction was heated to 130˚C for 96 h before filtering solids and washing with acetone. The solids were soaked in methanol for 24 h changing out the solvent 3 times over that period. An additional soak was performed in acetone following the same procedure. The light brown solids were filtered and dried under vacuum before characterization. (38.4 mg 74.8%). FT-IR (powder, ATR): 1569, 1515, 1430, 1409, 1360, 1296, 1176, 1120, 1054, 1015, 919, 862, 843, 816, 739, 690, 512. CP-MAS 13C NMR (75.5 MHz, δ ppm): 167.8, 160.6, 146.4, 138.7, 138.4, 136.2,

128.0, 127.9. Elemental analysis for $(C_{33}H_{15}O_3N_6)_n$: Calculated: C (72.92%), H (2.78%), N (15.46%). Observed: C (66.43%), H (3.31%), N (14.79%).

D. BBO Analog Trials

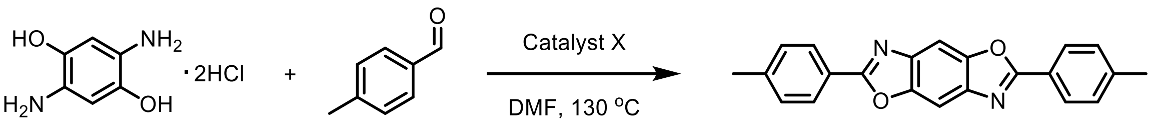

BBO-1

X
NaCN = 62%
NaN_3 = 37%
$NaSCH_3$ = 21%
No. Cat. = 11%

Figure S1. Synthesis of BBO-1

BBO-1: A dry 25 mL flask with stirbar was loaded with DABD (106.5 mg, 0.5 mmol, 0.5 eq), and DMF (8.3 mL). The reaction flask was purged with nitrogen and cooled to -15 ^{o}C, in an ethylene glycol/CO_2 bath. Once cooled, p-tolualdehyde (0.11 mL, 1.0 mmol, 1 eq) was added via microsyringe. The flask was kept under nitrogen, and stirred at -15 ^{o}C for 3h. The reaction was removed from the cooling bath and warmed to room temperature in a water bath overnight. The reaction was opened to air and the chosen nucleophilic catalyst (1.0 mmol, 1 eq) was dissolved in a chosen solvent (1.3 mL) before adding to the solution. NaCN and $NaSCH_3$ were dissolved in methanol, but due to solubility reasons DMF was chosen to dissolve NaN_3. The mixture was heated at 130˚C for 24 hours. After, the mixture was cooled to room temperature, and diluted with water (15 mL). A precipitate formed, which was vacuum filtered, washing with water. The solids were collected and recrystallized from toluene to afford BBO-1 as a brown solid. ^{1}H-NMR ($CDCl_3$, 400 MHz) δ 8.16 (d, 4H); 7.88 (s, 2H); 7.34 (d, 4H). ^{13}C-NMR ($CDCl_3$, 150 MHz) 164.6, 148.6, 142.4, 140.5, 129.7, 127.6, 124.4, 100.7, 21.8.

BBO-1 deoxygenated: A dry 25 mL flask was loaded with DMF (8.3 mL) and p-tolualdehyde (0.11 mL, 1.0 mmol, 1 eq), which were degassed using the freeze-pump-thaw method 3 times and backfilled with N_2. The solution was transferred via syringe to a round bottom flask under inert atmosphere containing a stirbar and DABD (106.5 mg, 0.5 mmol, 0.5 eq). The reaction was cooled to -15 ^{o}C (ethylene glycol/CO_2) while remaining under N_2. The flask was kept under nitrogen, and stirred at -15 ^{o}C for 3h. The reaction was removed from the cooling

bath and warmed to room temperature in a water bath overnight. NaCN (1.0 mmol, 1 eq) was dissolved in a MeOH (1.3 mL) and was degassed using the freeze-pump-thaw method three times and backfilled with N_2. The NaCN solution was transferred via syringe to the reaction flask. The mixture was heated at 130˚C for 24 hours, while remaining under nitrogen. After, the mixture was cooled to room temperature, and diluted with water (15 mL) and prepared as above.

Table S1. Summary of synthesis trials of BBO-1 with different nucleophiles

Nucleophile (Nuc)	Eq (Nuc)	Temp (˚C)	Reaction Time (h)	Yield of oxazole
NaCN	1	130	24	62%
NaN_3	1	130	24	37%
$NaSCH_3$	1	130	24	21%
None	1	130	24	11%
*NaCN	1	130	24	<1%

*Trial was performed without oxygen present

E. BBO-COF 2 Formation Studies

General Synthetic Procedure for BBO-COF 2: BBO-COF 2 was synthesized as previously reported, varying the nucleophilic catalyst's identity.[6] To test the different nucleophiles for this reaction, the chosen catalyst (1.0 mmol, 1 eq) was dissolved in a chosen solvent (1.3 mL) before adding to the solution. NaCN and $NaSCH_3$ were dissolved in methanol, but due to solubility reasons DMF was chosen to dissolve NaN_3.

Table S2. Summary of BBO-COF 2 synthesis with different nucleophilic catalysts

Trial	Nucleophile (Nuc)	Temp (˚C)	Reaction Time	Porosity (m^2/g)	% Yield
Nucleophiles	NaCN	130	4d	1106	62%
	NaN_3	130	4d	1033	54%
	$NaSCH_3$	130	4d	236	40%
	None	130	4d	169	40%
	*NaCN	130	4d	142	13%

*Sample was run without oxygen present (deoxygenated)

E-1 BBO-COF 2 Nucleophile Trials

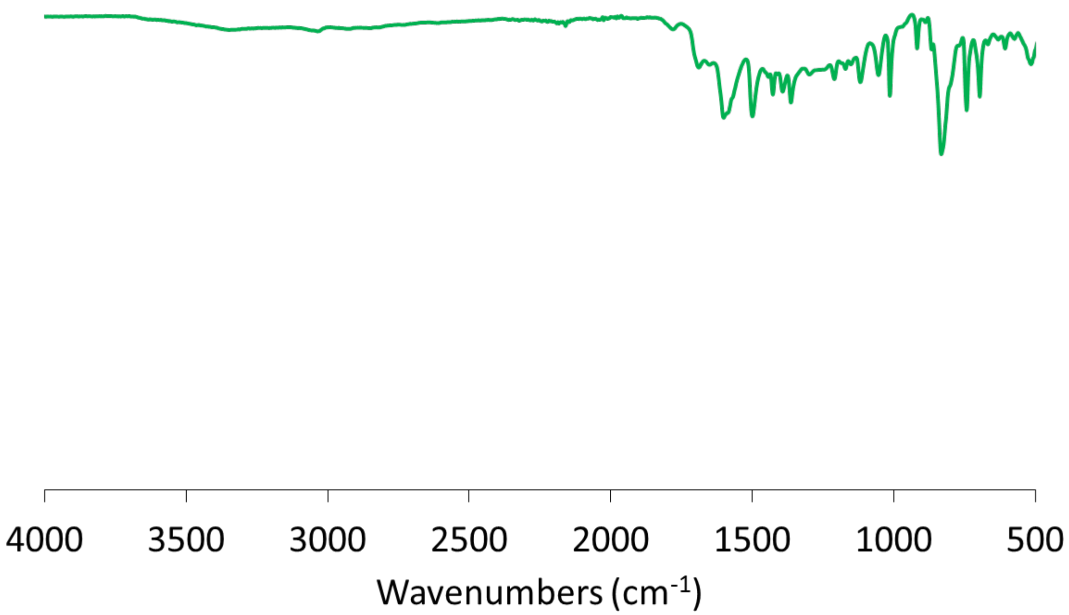

Figure S2. FT-IR spectra of BBO-COF 2 synthesized with NaN_3

Table S3. FT-IR peak assignments for BBO-COF 2 synthesized with NaN_3

Peak (cm^{-1})	Assignment
**1624**	**C=N stretch of benzoxazole**
**1118**	**C-O stretch of benzoxazole**

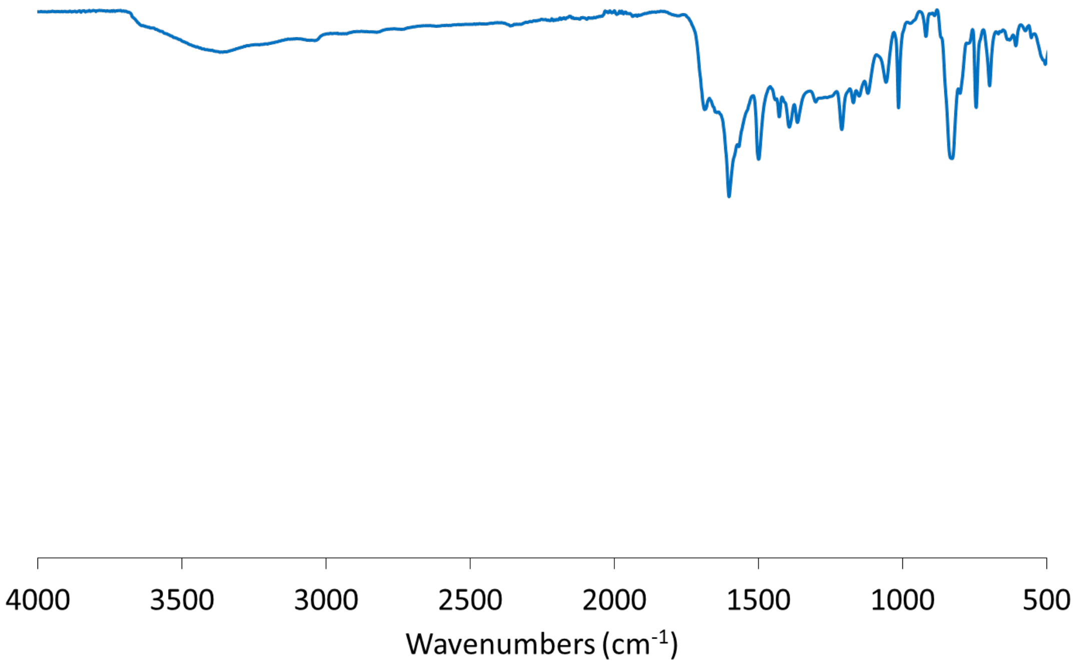

Figure S3. FT-IR spectra of BBO-COF 2 synthesized with $NaSCH_3$

Table S4. FT-IR peak assignment for BBO-COF 2 synthesized with $NaSCH_3$

Peak (cm^{-1})	Assignment
3369	O-H stretch
1630	C=N stretch of benzoxazole
1120	C-O stretch of benzoxazole

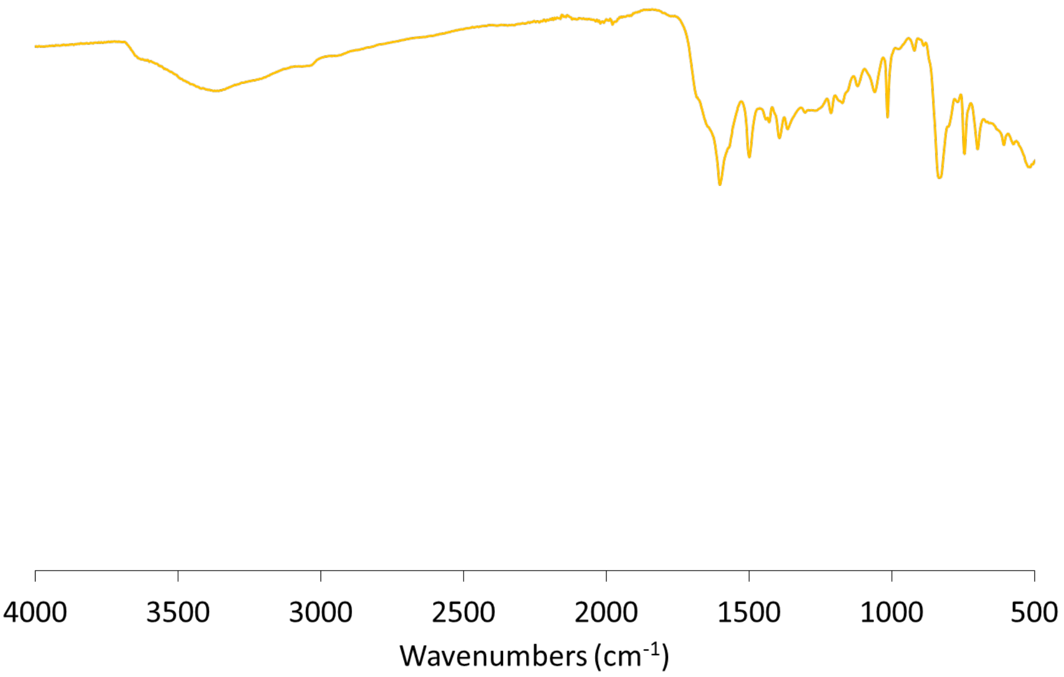

Figure S4. FT-IR spectra of BBO-COF 2 synthesized without a catalyst

Table S5. FT-IR peak assignments for BBO-COF 2 synthesized without a catalyst

Peak (cm^{-1})	Assignment
3360	O-H stretch
1666	C=N stretch of benzoxazole
1212	C-O stretch of benzoxazole

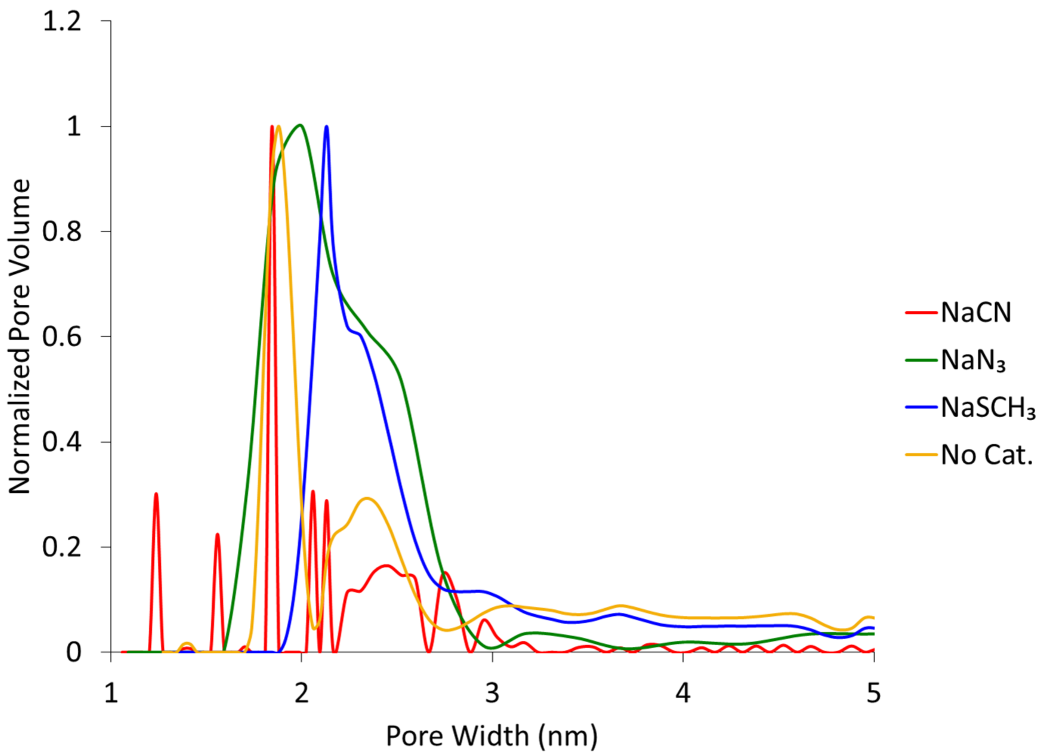

Figure S5. Normalized pore size distributions of BBO-COF 2 synthesized with different nucleophiles

E-2 BBO-COF 2 Deoxygenated Trial

Procedure for BBO-COF 2 deoxygenated:

BBO-COF 2 deoxygenated was synthesized by modifying the previously reported synthesis.[6] For the deoxygenated sample, DMF, and methanol were degassed using the freeze-pump-thaw method three times each and backfilled with N_2. The reaction was performed under nitrogen throughout the four day heating process. NaCN was used as the catalyst.

In the deoxygenated sample we see a peak signifying significant –OH stretching and a shift in the C=N imine stretch that corresponds to the imine linked instead of the benzoxazole linked polymer.

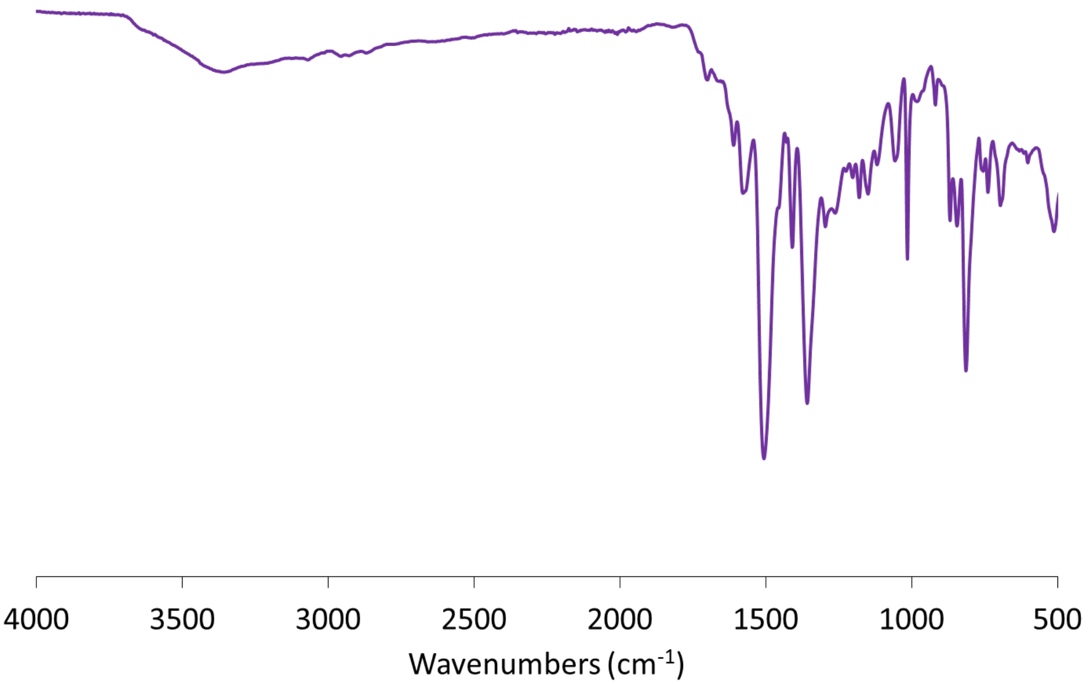

Figure S6. FT-IR spectra of BBO-COF 2 deoxygenated

Table S6. FT-IR peak assignments for BBO-COF 2 deoxygenated

Peak (cm^{-1})	Assignment
3361	O-H stretch
1701	C=N stretch of Imine
1205	C-O stretch

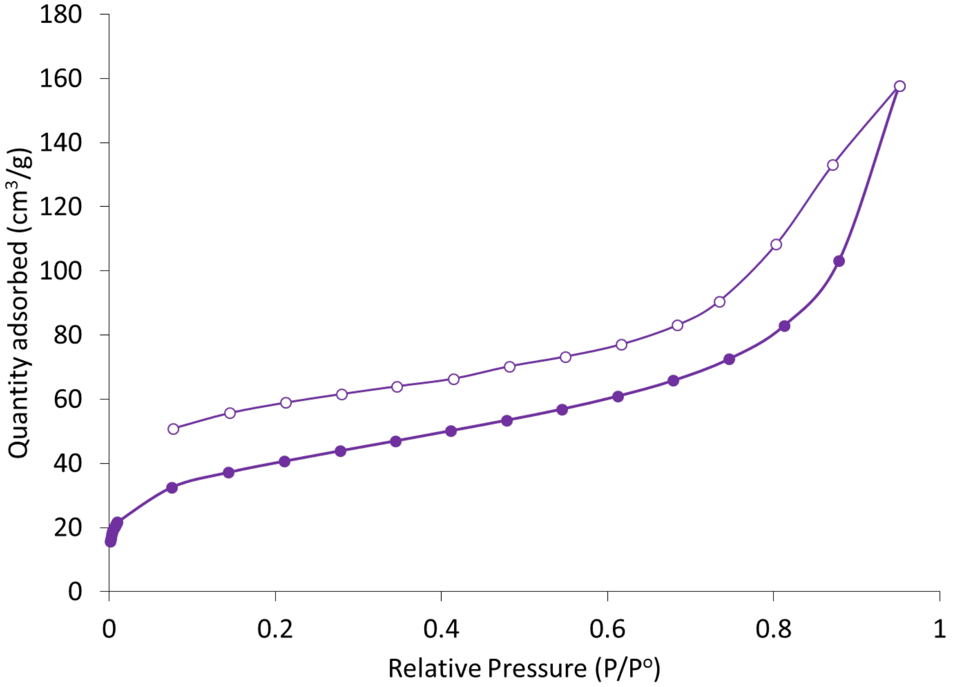

Figure S7. N_2 isotherm of BBO-COF 2 deoxygenated

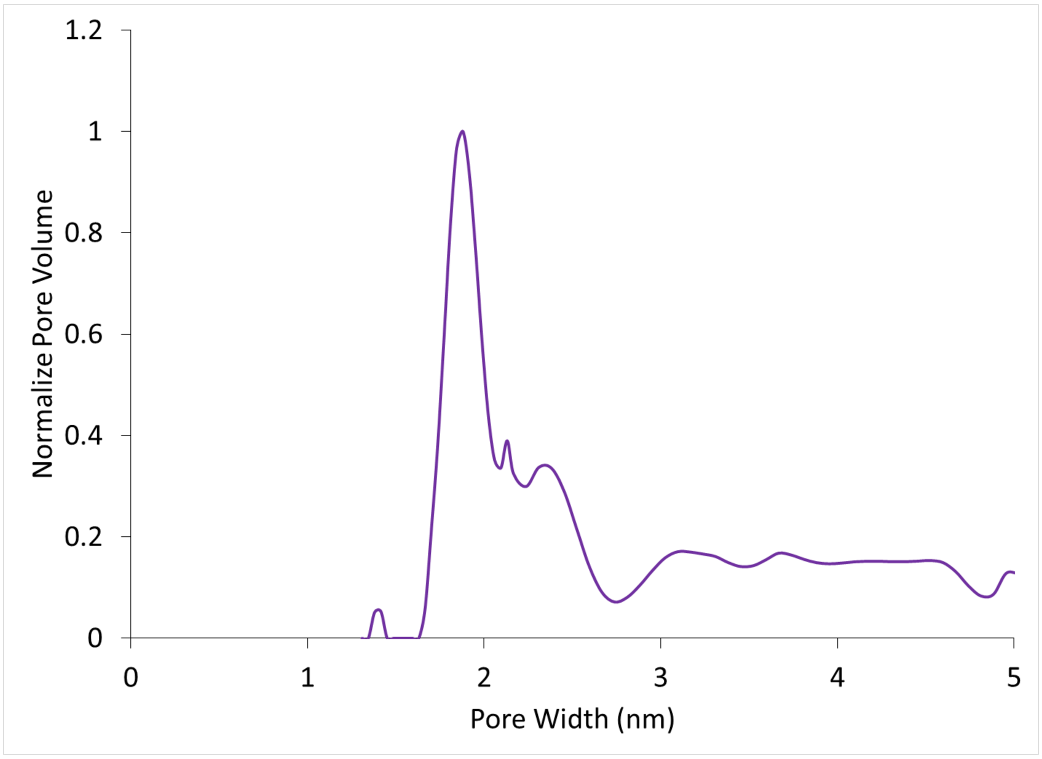

Figure S8. Pore size distribution of BBO-COF 2 deoxygenated

F. BBO-COF-3 Formation Studies

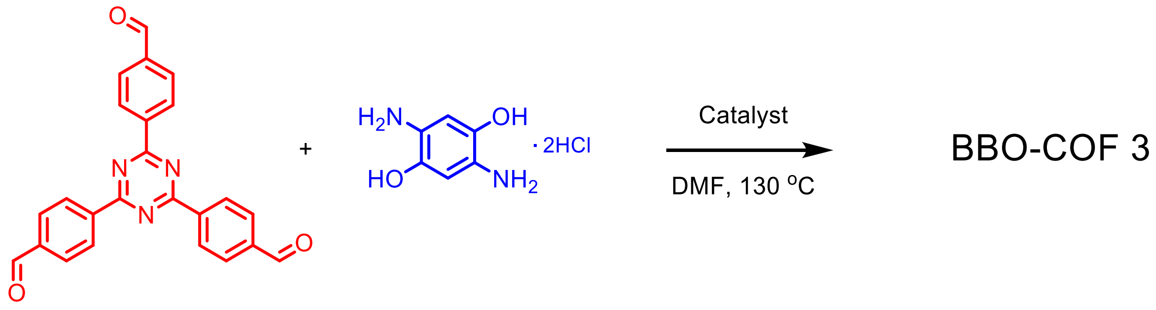

Figure S9. Synthetic scheme for BBO-COF 3

Procedure for BBO-COF 3 nucleophile trials: The nucleophile trials were run using the synthetic procedure found above for BBO-COF 3, varying the nucleophilic catalyst's identity. The chosen catalyst (1.0 mmol, 1 eq) was dissolved in a chosen solvent (1.3 mL) before adding to the solution. NaCN and $NaSCH_3$ were dissolved in methanol, but due to solubility reasons DMF was chosen to dissolve NaN_3.

Procedure for BBO-COF 3 time trials: The time trials were run using the synthetic procedure found above for BBO-COF 3, with NaCN as the catalyst. The polymer trials were stopped at various times throughout the heating process. The before heating trial was stopped prior to the exposure to air and addition of NaCN. The heated samples were stopped at 24h, 48h, 72h, and 96h to determine the required length of reaction time for complete oxazole formation. All 5 different time trials were worked up by filtering the solids and soaking them in methanol for 24 h, changing out the solvent 3 times, followed by an additional soak in acetone for 24 h, refreshing the solvent 3 times. The solids were filtered and dried under vacuum before characterization.

Table S7. Summary of trials for BBO-COF 3.

Trial	Nucleophile	Temp (˚C)	Reaction Time	Porosity (m^2/g)	% Yield
Nucleophiles	NaCN	130	4d	2039	74.8%
	NaN_3	130	4d	1439	71.6%
	$NaSCH_3$	130	4d	1697	67.6%
	None	130	4d	386	64.5%
Reaction Time	NaCN	130	Before Heating	Nonporous	83.2%
	NaCN	130	1 d	704	79.6%
	NaCN	130	2 d	1435	73.7%
	NaCN	130	3 d	1295	74.1%
	NaCN	130	4 d	2039	74.8%
	*NaCN	130	4 d	1366	47.9%

*Sample was run without oxygen present (deoxygenated)

F-1 BBO-COF 3 Nucleophile Trials

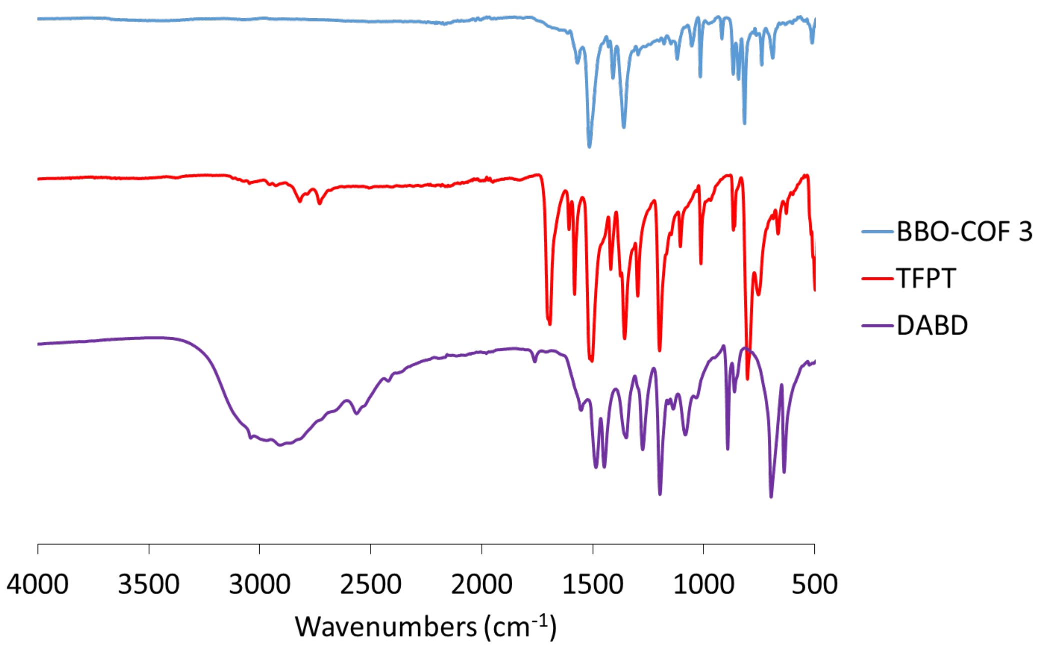

Figure S10. FT-IR spectra of BBO-COF 3 synthesized with NaCN compared with the monomers.

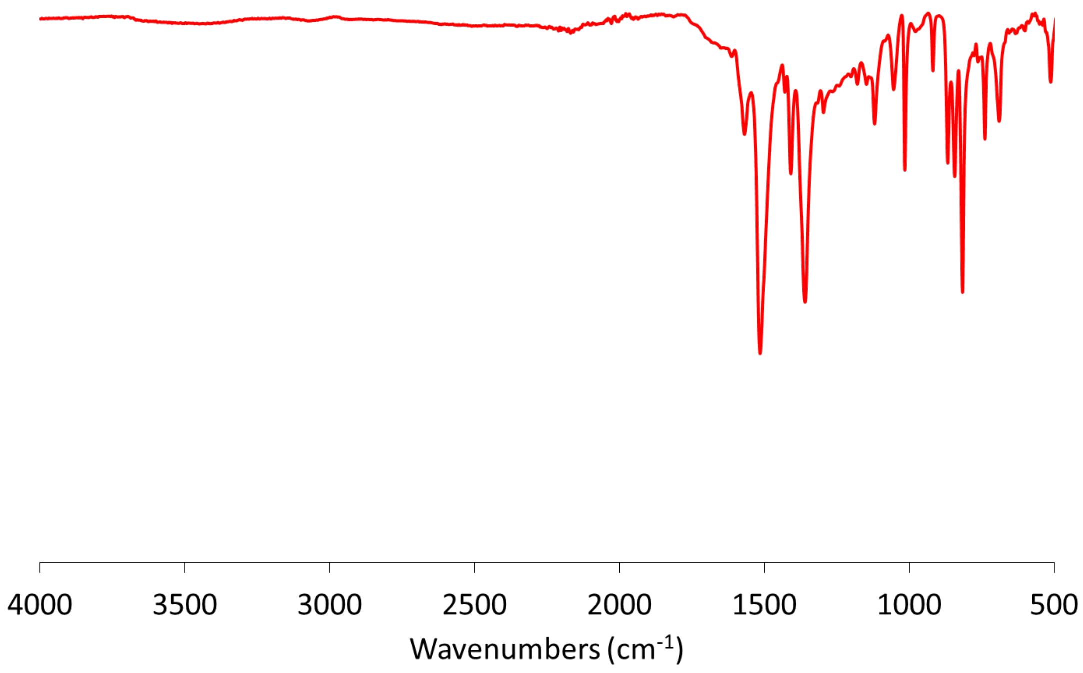

Figure S11. FT-IR spectra of BBO-COF 3 synthesized with NaCN

Table S8. FT-IR peak assignments for BBO-COF 3 synthesized with NaCN

Peak (cm^{-1})	Assignment
1640	C=N stretch of benzoxazole
1569	C=N stretch of triazine
1119	C-O stretch of benzoxazole

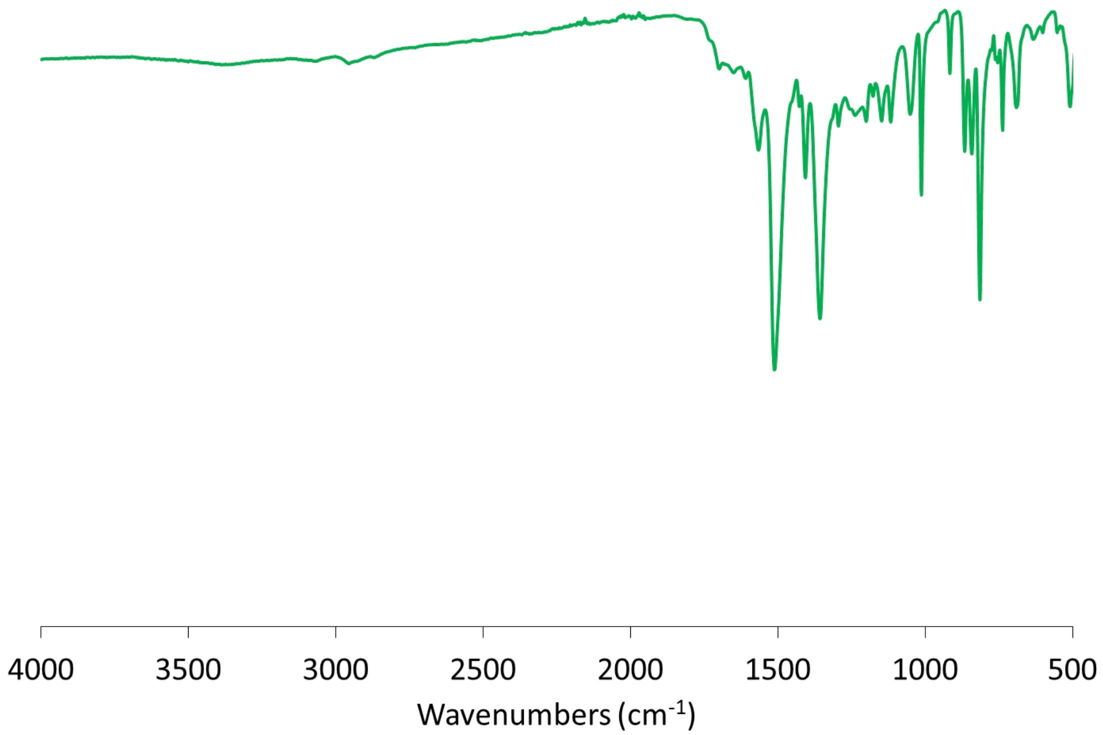

Figure S12. FT-IR spectra of BBO-COF 3 synthesized with NaN_3

Table S9. FT-IR peak assignments for BBO-COF 3 synthesized with NaN_3

**Peak (cm^{-1})**	**Assignment**
**1647**	**C=N stretch of benzoxazole**
**1566**	**C=N stretch of triazine**
**1117**	**C-O stretch of benzoxazole**

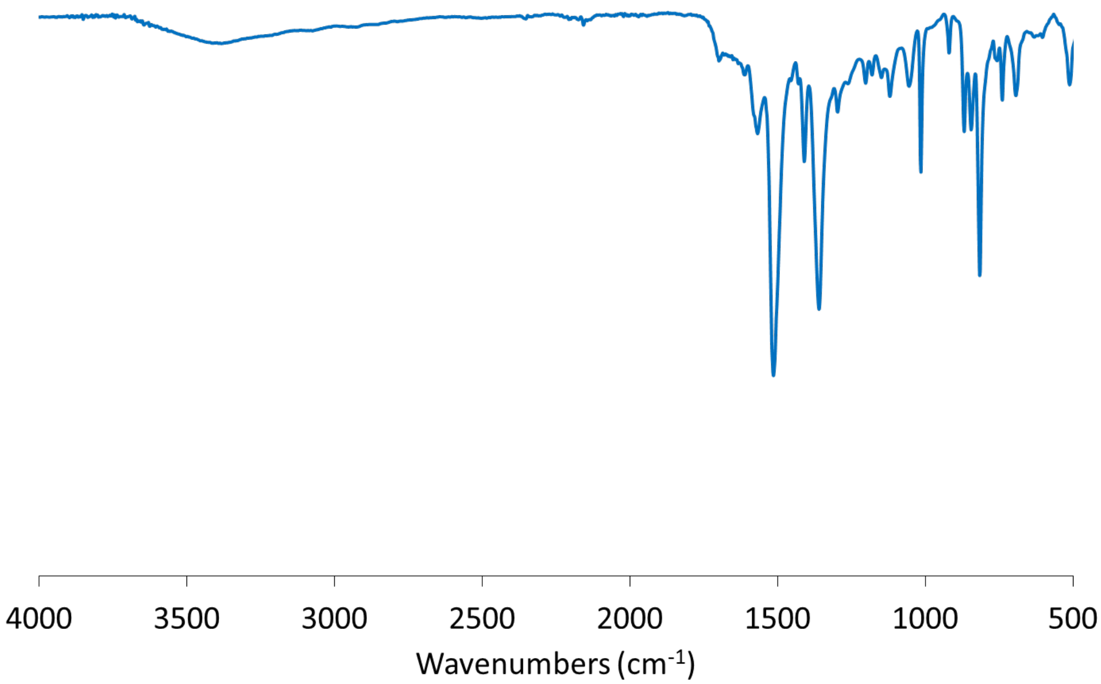

Figure S13. FT-IR spectra of BBO-COF 3 synthesized with $NaSCH_3$

Table 10. FT-IR peak assignments for BBO-COF 3 synthesized with $NaSCH_3$

Peak (cm^{-1})	Assignment
3384	O-H stretch
1624	C=N stretch of benzoxazole
1568	C=N stretch of triazine
1120	C-O stretch of benzoxazole

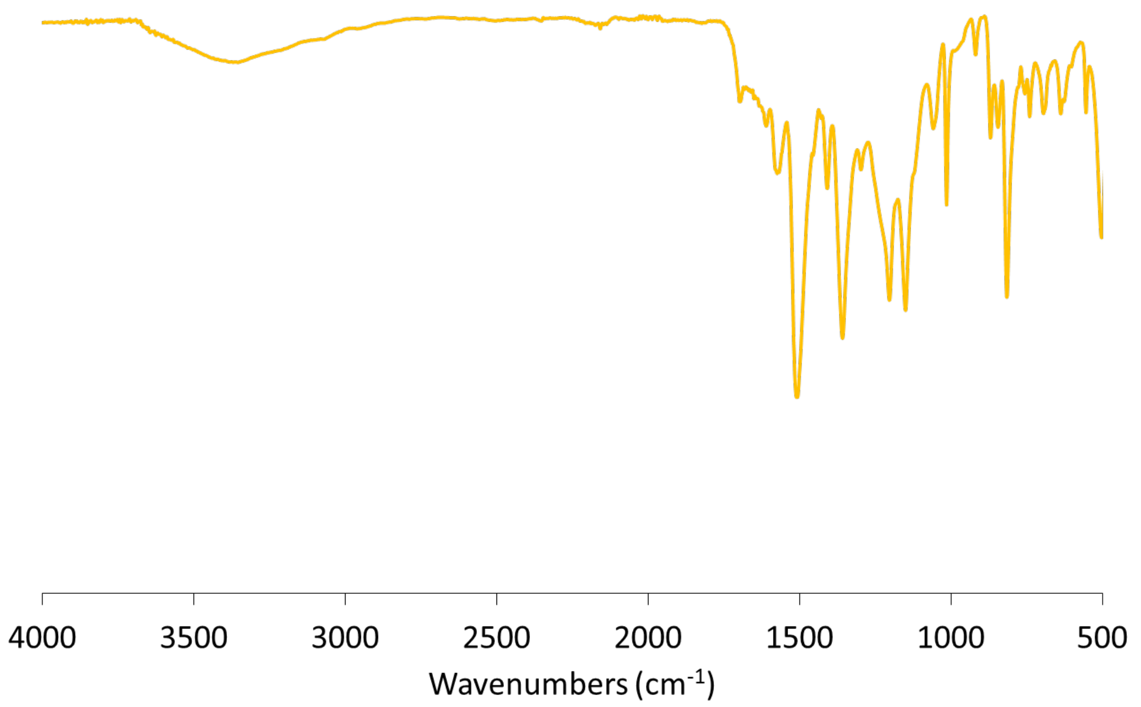

Figure S14. FT-IR spectra of BBO-COF 3 synthesized without a catalyst

Table S11. FT-IR peak assignments for BBO-COF 3 synthesized without a catalyst

Peak (cm^{-1})	Assignment
3352	O-H stretch
1695	C=N stretch of imine
1574	C=N stretch of triazine
1149	C-O stretch

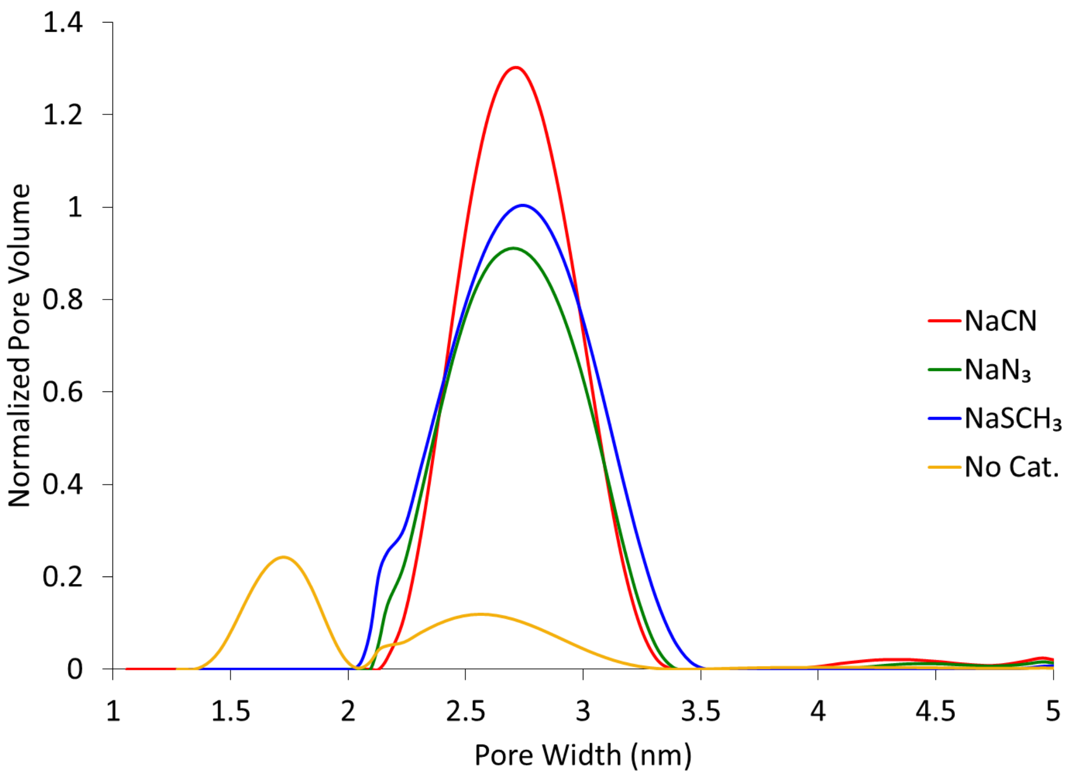

Figure S15. Normalized pore size distributions of BBO-COF 3 synthesized with different nucleophiles

F-2 BBO-COF 3 Reaction Time Trials

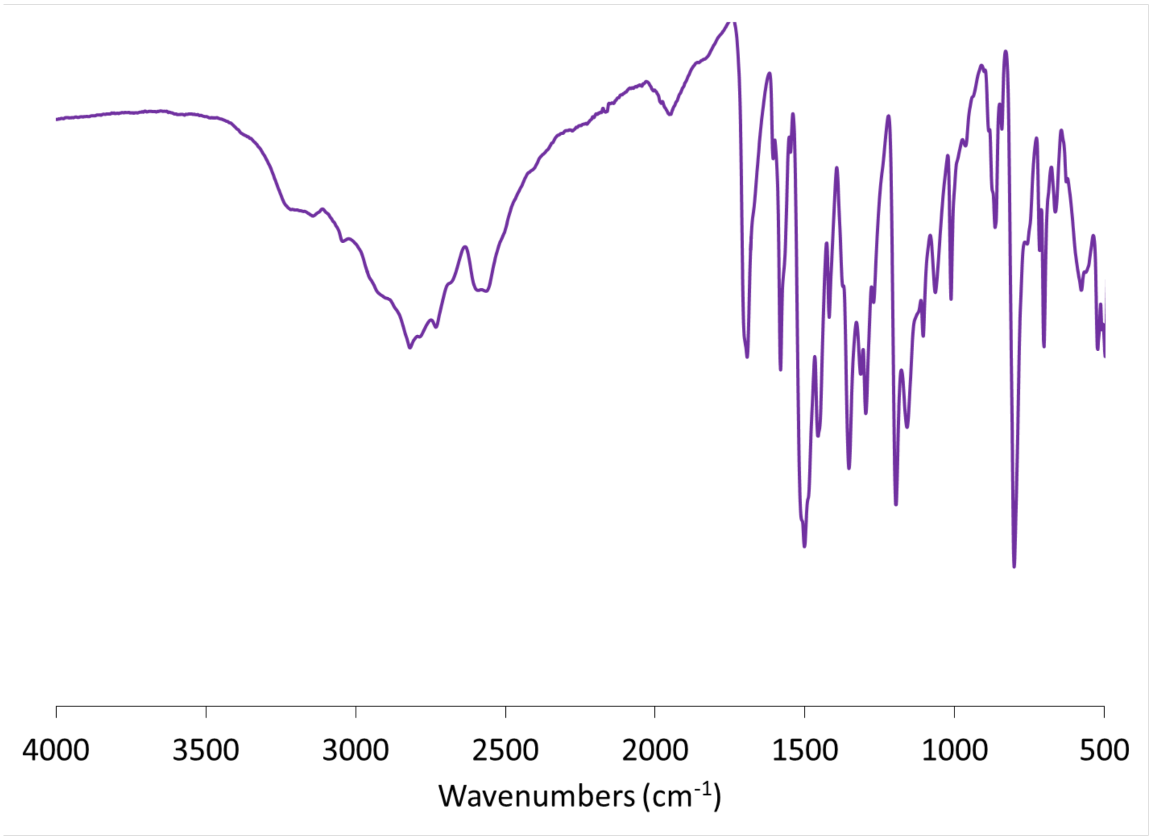

Figure S16. FT-IR spectra of a ground mixture of BBO-COF 3 monomers, DABD and TFPT

Table S12. FT-IR peak assignments for a ground mixture of BBO-COF 3 monomers, DABD and TFPT

Peak (cm^{-1})	Assignment
2814	O-H stretch of DABD monomer
1696	C=O stretch of aldehyde
1581	C=N stretch of triazine

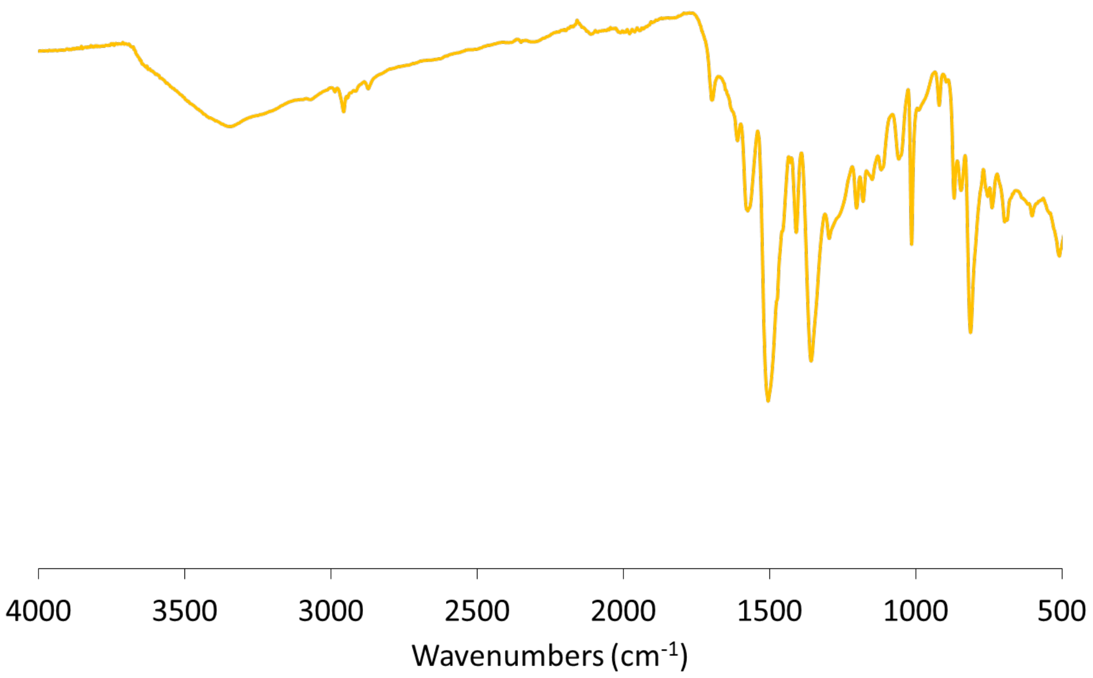

Figure S17. FT-IR spectra of BBO-COF 3 after 1 day of heating

Table S13. FT-IR peak assignments for BBO-COF 3 after 1 day of heating

Peak (cm^{-1})	Assignment
3343	O-H stretch
1693	C=N stretch of imine
1573	C=N stretch of triazine
1012	C-O stretch

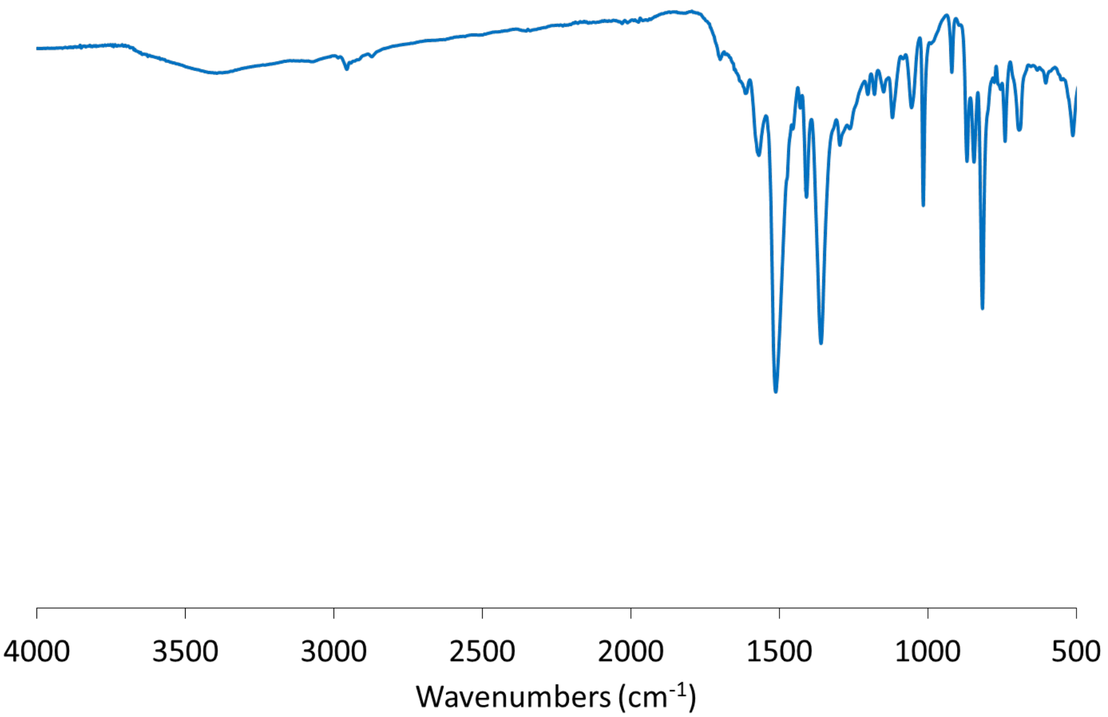

Figure S18. FT-IR spectra of BBO-COF 3 after 2 days of heating

Table S14. FT-IR peak assignments for BBO-COF 3 after 2 days of heating

Peak (cm^{-1})	Assignment
3379	O-H stretch
1698	C=N stretch of imine
1567	C=N stretch of triazine
1015	C-O stretch

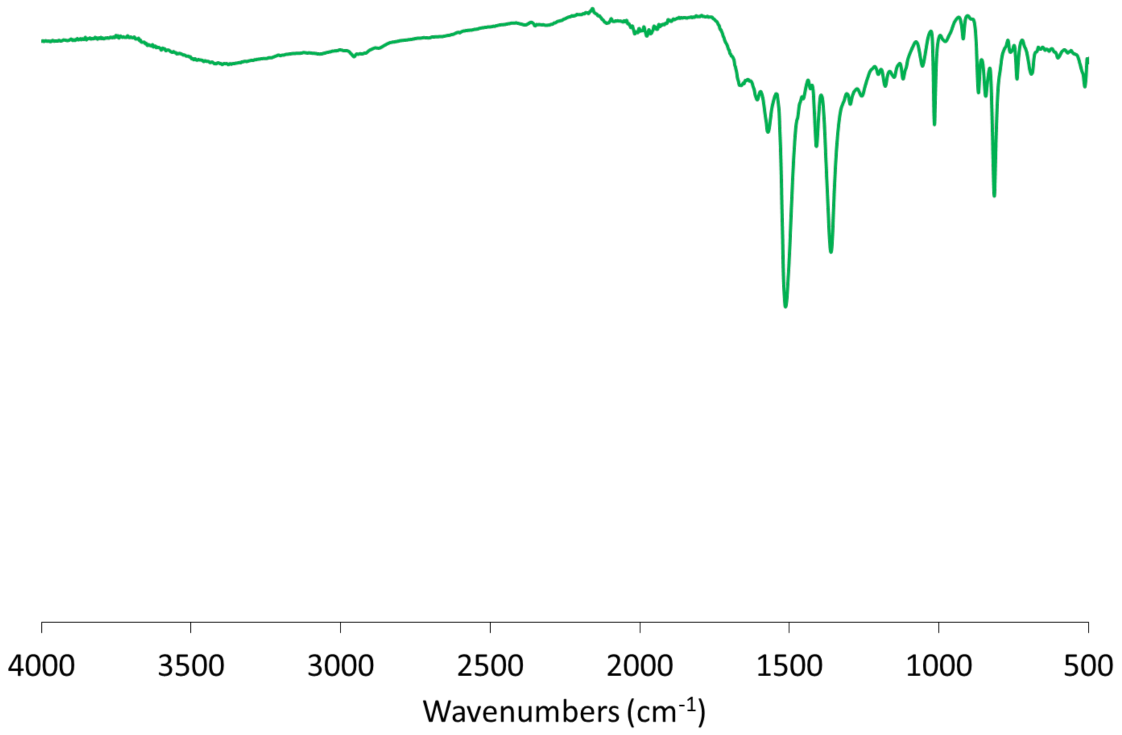

Figure S19. FT-IR spectra of BBO-COF 3 after 3 days of heating

Table S15. FT-IR peak assignments for BBO-COF 3 after 3 days of heating

Peak (cm^{-1})	Assignment
**3369**	**O-H stretch**
**1660**	**C=N stretch of benzoxazole**
**1572**	**C=N stretch of triazine**
**1015**	**C-O stretch of benzoxazole**

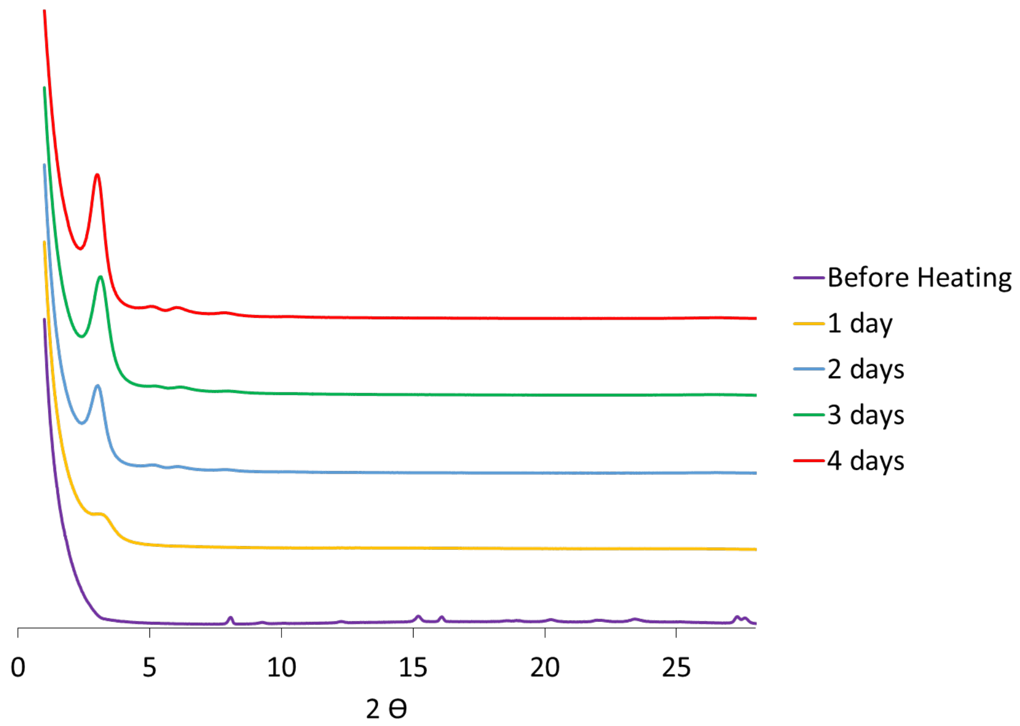

Figure S20. Normalized PXRD of BBO-COF 3 synthesized over different reaction times

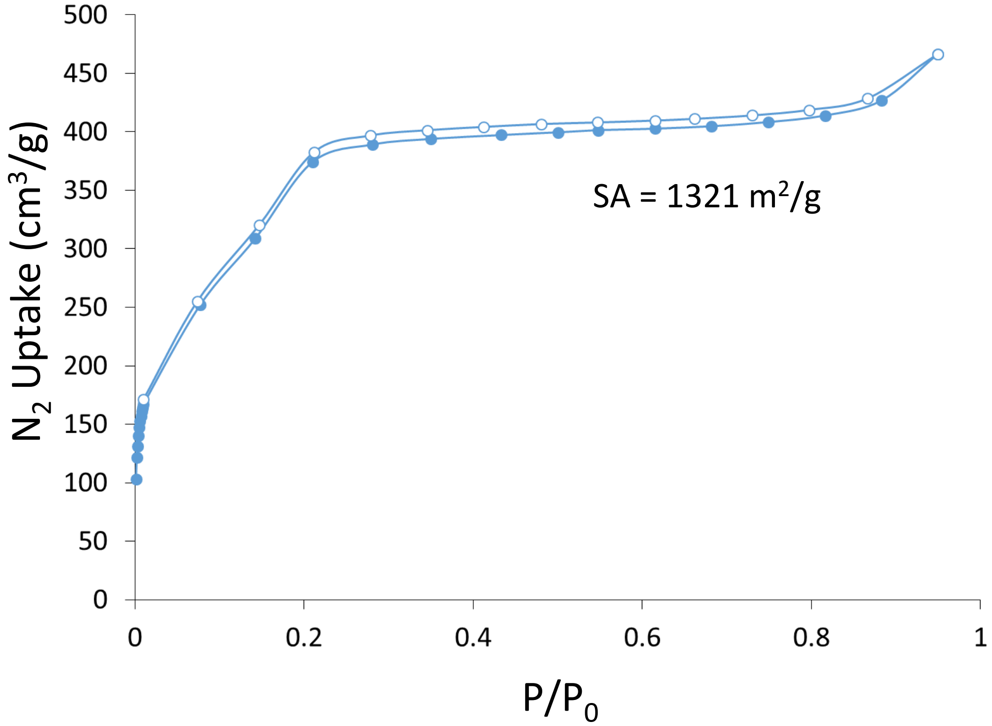

Figure S21. Nitrogen isotherm at 77 K for BBO-COF 3 after a 7-day reaction time.

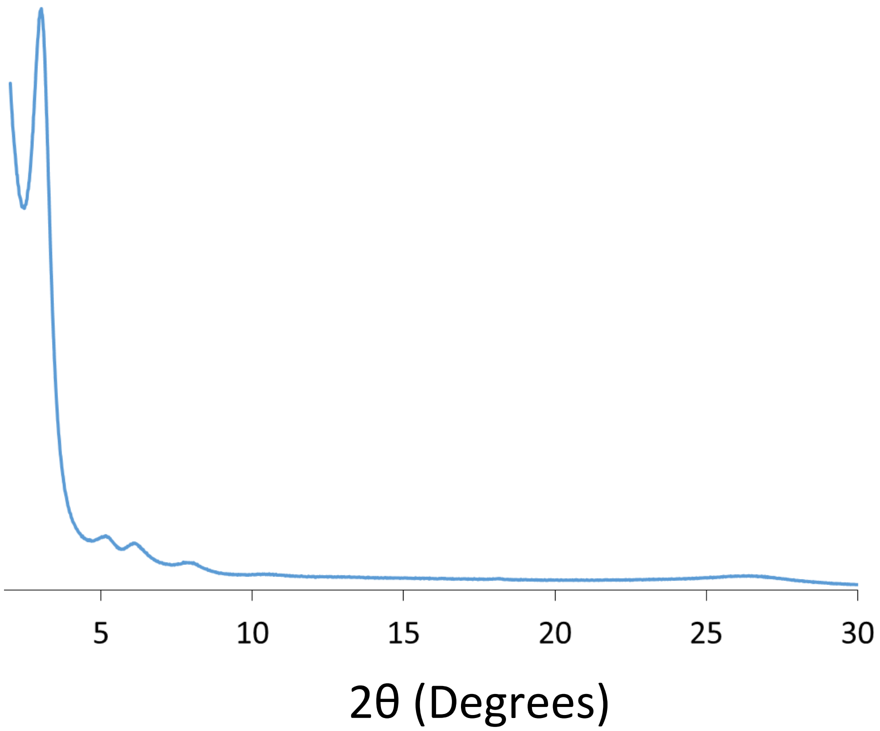

Figure S22. PXRD of BBO-COF 3 after a 7-day reaction time.

F-3 BBO-COF 3 Deoxygenated Trial

Procedure for BBO-COF 3 deoxygenated:
BBO-COF 3 deoxygenated was synthesized by modifying the procedure mentioned above.[6] For the deoxygenated sample, DMF, and methanol were degassed using the freeze-pump-thaw method three times each and backfilled with N_2. The reaction was performed under nitrogen throughout the four day heating process. NaCN was used as the catalyst.

In the deoxygenated sample we see a peak signifying significant –OH stretching and a shift in the C=N imine stretch that we corresponds to the imine linked instead of the benzoxazole linked polymer. For BBO-COF 3 we also see a 0.2 nm shift in the major pore of the material, which can happen due to the increased slip stacking cause by the flexible imine linkage.

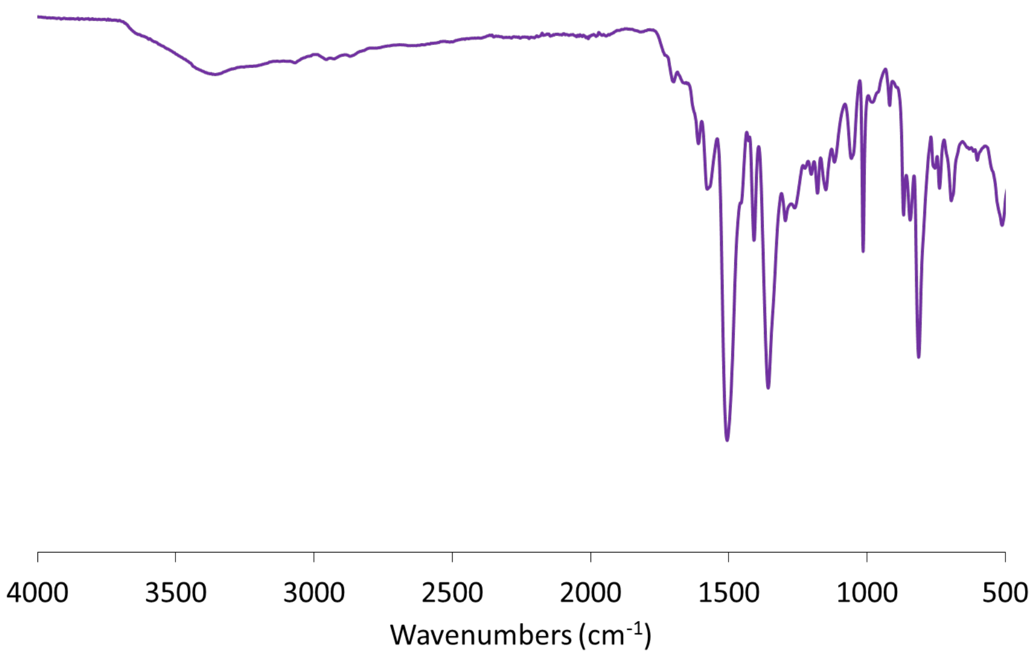

Figure S23. FT-IR spectrum for BBO-COF 3 deoxygenated

Table S16. FT-IR peak assignments for BBO-COF 3 deoxygenated

Peak (cm^{-1})	Assignment
3359	O-H stretch
1699	C=N stretch of imine
1572	C=N stretch of triazine
1016	C-O stretch

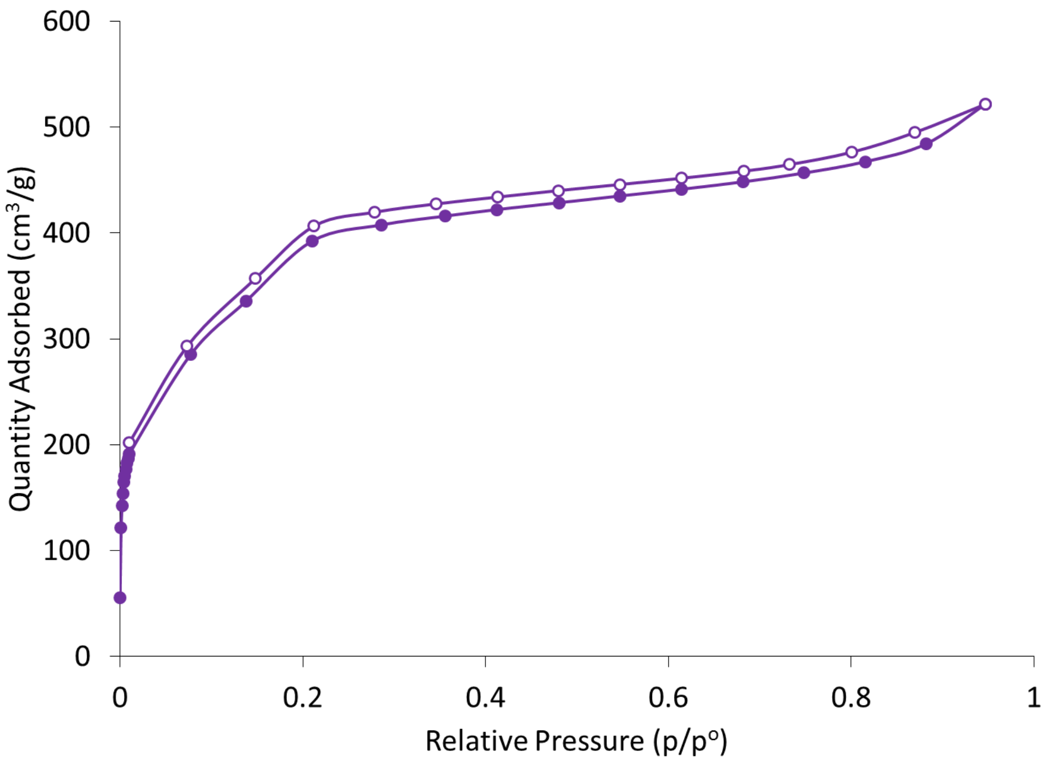

Figure S24. N_2 isotherm for BBO-COF 3 deoxygenated

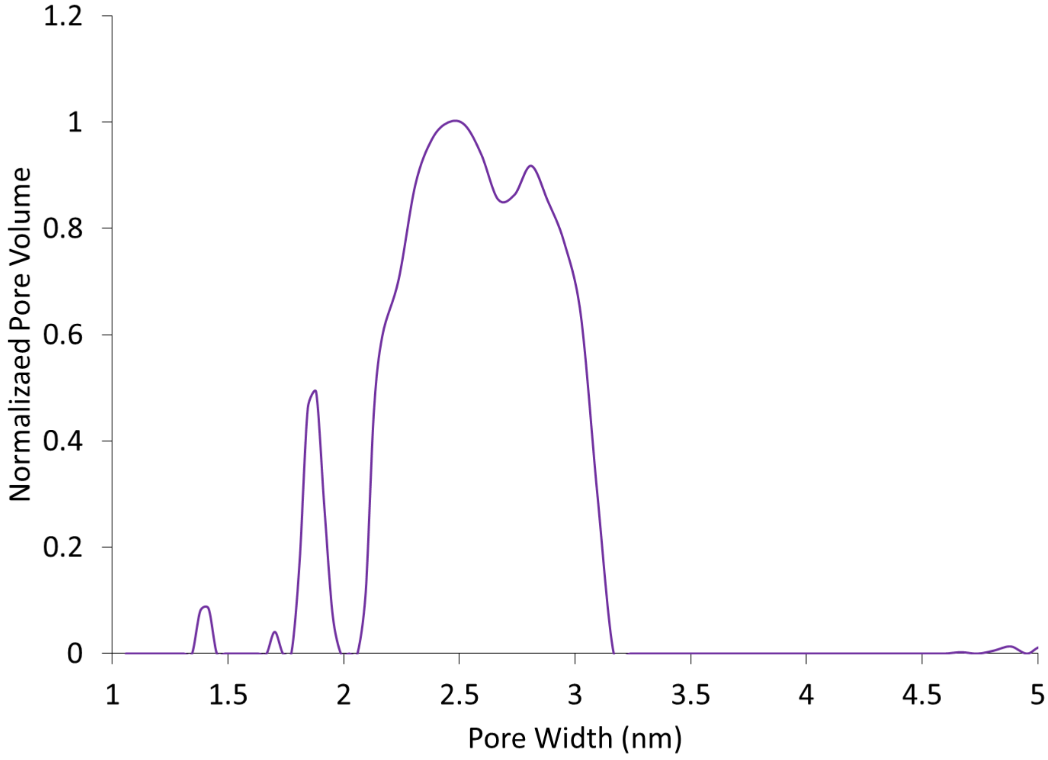

Figure S25. Pore size distribution for BBO-COF 3 deoxygenated

G. BBO-COF 3 Experimental & Simulated PXRD Data

The simulated PXRD profiles were performed using Materials Studio 7.0 using the unit cell precursors shown in Figure S26. Before the simulations were performed, each precursor was optimized using the geometry optimization task and Universal Forcefield parameters from the Forcite module. Each structure was then modeled using a primitive hexagonal unit cell with a P6 space group. The a = b parameters were estimated by measuring the distance between the BBO phenyl ring of the linkers for each COF. The c parameter was arbitrarily set at 3.4 Å. The staggered molecular models were performed by offsetting the initial structures by half of the a=b parameters using a gra ($P6_3$/mmc) space group and c parameter of 6.7 A. Simulation of the possible structures was performed using Reflux Plus module to produce the expected PXRD profiles. The experimental PXRDs were then subjected to a Pawley refinement using Pseudo-Voigt peak shape function and Berar-Baldinozzi asymmetry correction function to produce the refined PXRD profile.

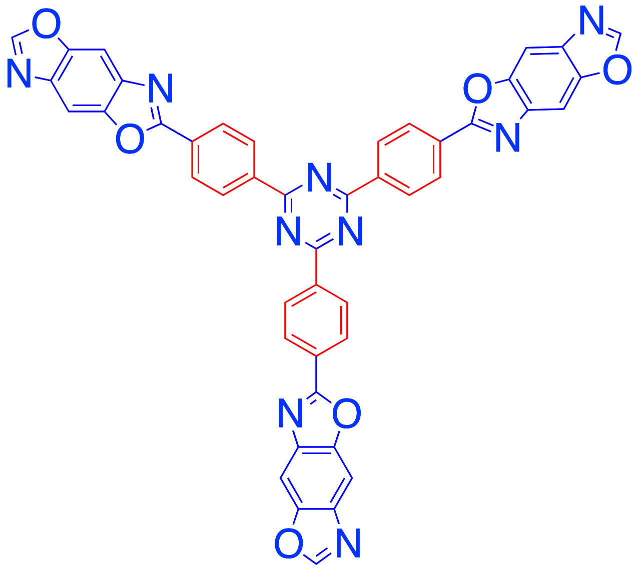

Figure S26. Precursor used to construct the hexagonal unit cell for BBO-COF 3.

PXRD Analysis Discussion: Powder X-ray diffraction (PXRD) was used to evaluate the crystallinity of BBO-COF **3** (Fig. S27). Taking into account the variations between the observed and predicted pore size distributions, we modeled BBO-COF **3** using a *P6* hexagonal unit cell wherein the adjacent layers were offset by 6 Å. BBO-COF **3** displayed an intense peak at 2.95 followed by smaller peaks at 5.11, 5.89, 7.82, 26.2°, which correspond to the (100), (110), (200), (210), and (001) planes, respectively. Pawley refinement of the experimental PXRD data afforded unit cell parameters of a = b = 34.595 Å and c = 3.4 (residuals R_p = 3.20%, R_{wp} = 4.55%). The experimental PXRD data is consistent with the simulated hexagonal unit cell in which the layers are slipped by 6 Å. We also considered the possibility of BBO-COF **3** forming staggered gra ($P6_3$ /mmm) stacking layers, but the simulated patterns did not match the experimental data (Fig. S29).

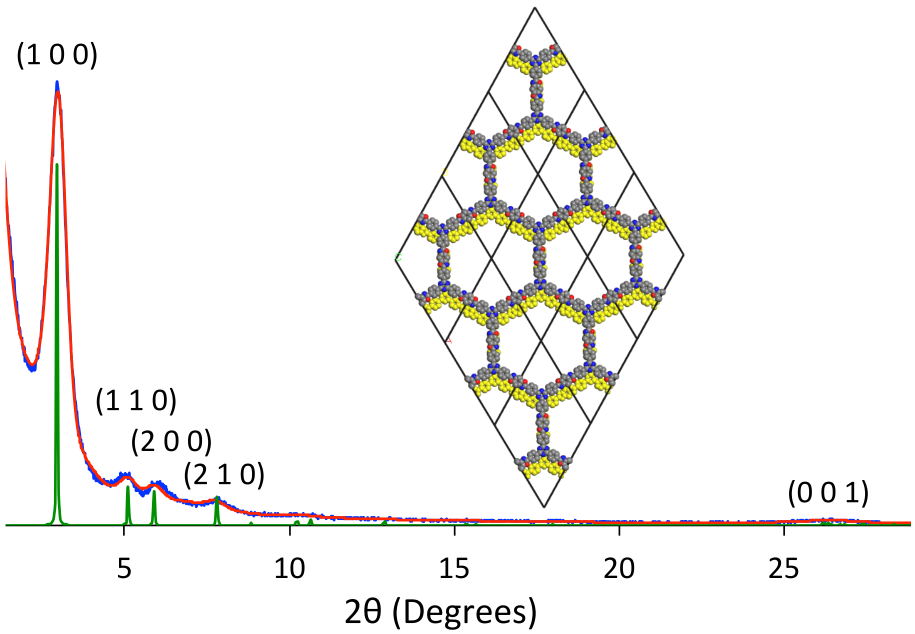

Figure S27. Indexed experimental (blue) and Pawley refined (red) PXRD data of BBO-COF **3** compared to the simulated hexagonal unit cell (green) with an offset of 6 Å and a view along the c direction.

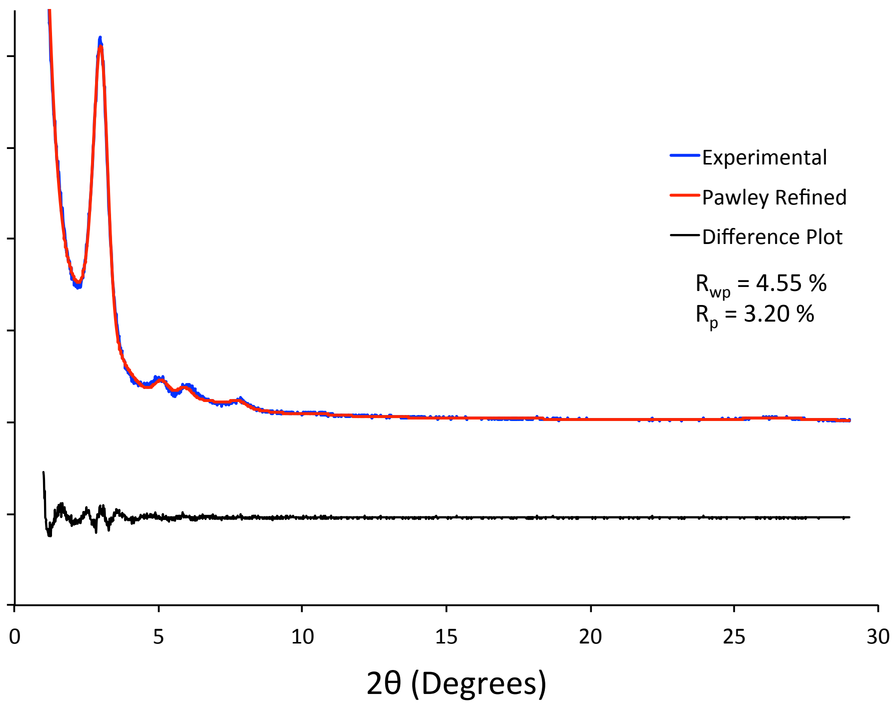

Figure S28. Experimental (blue), Pawley refined (red), and difference plot (black) BBO-COF 3.

Table S17. Fractional atomic coordinates for the P6 unit cell of BBO-COF 3 calculate using Materials Studio 7.0

**BBO-COF 3**			
Hexagonal, P6<br>a=b= 34.595, c = 3.4			
**Atom**	**x**	**y**	**z**
C1	0.414957	0.580507	1.203390
C2	0.439019	0.557393	1.203020
N3	0.419963	0.515597	1.204470
C4	0.454138	0.507057	1.203550
C5	0.451984	0.467082	1.204530
H6	0.419799	0.433412	1.206270
C7	0.491783	0.468837	1.203290
O8	0.499673	0.434900	1.203900
C9	0.544986	0.456600	1.202100
C10	0.569052	0.433489	1.202070
C11	0.546982	0.388605	1.203970
H12	0.508932	0.369451	1.205550
C13	0.569544	0.366349	1.204020
H14	0.550296	0.328300	1.205660
C15	0.614588	0.388606	1.202160

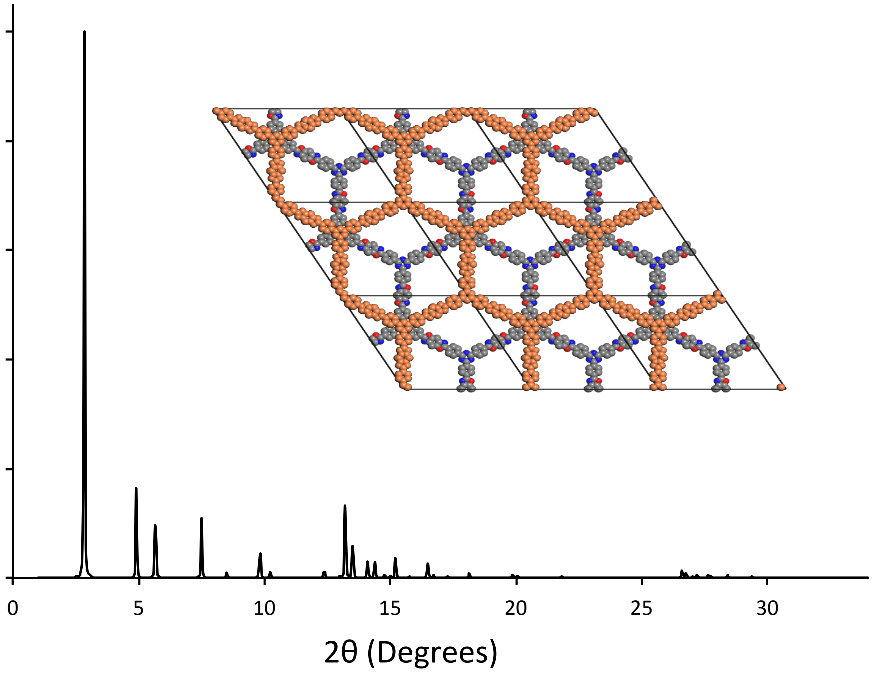

Figure S29. Simulated PXRD of BBO-COF 3 modeled using a gra unit cell.

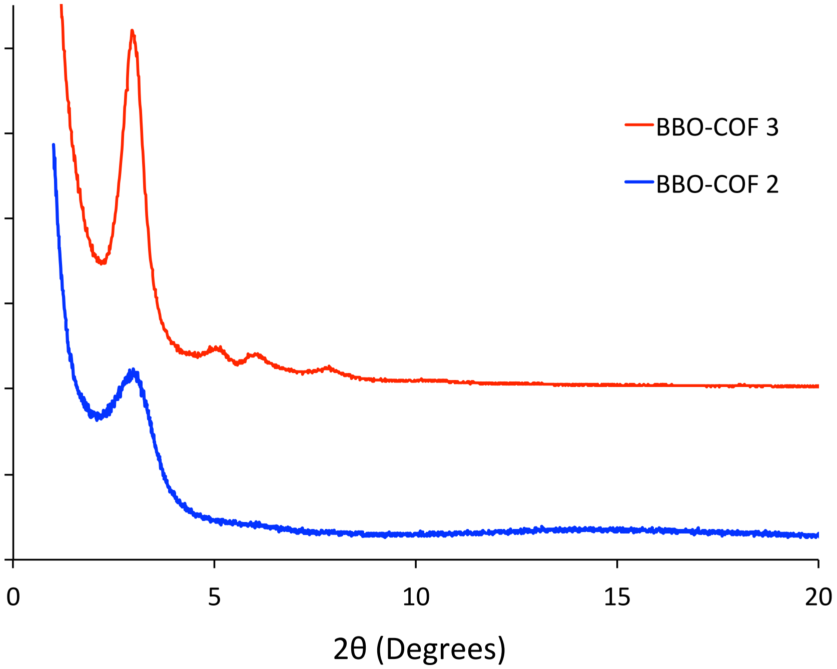

Figure S30. PXRD profiles for BBO-COF 2 and 3 synthesized using NaCN as a catalyst.

H. Solid State NMR Spectra

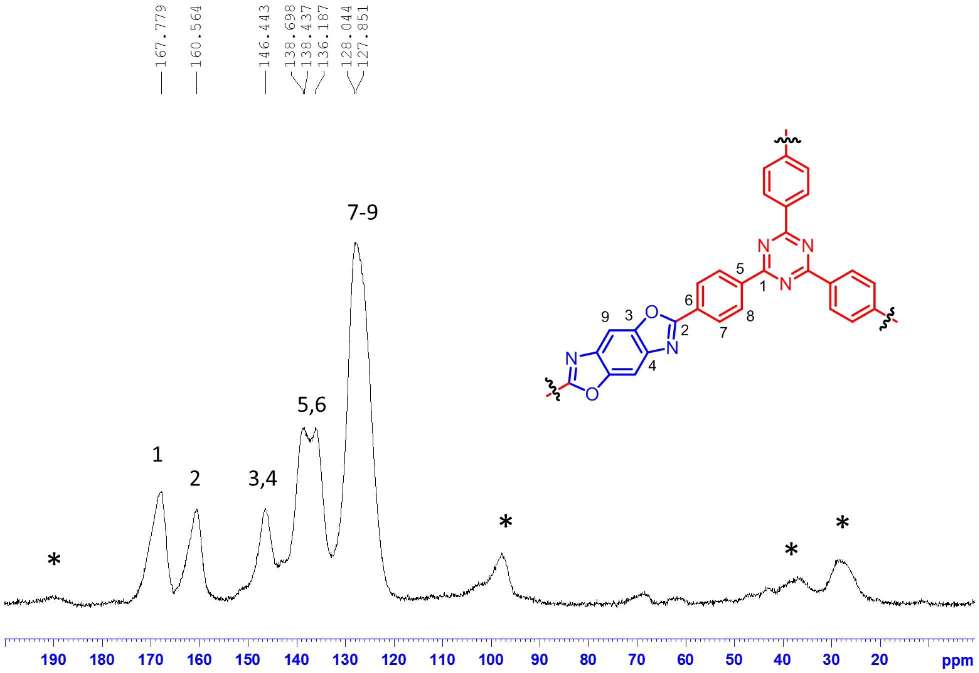

Figure S31. 150.9 MHz ^{13}C CP-MAS solid-state NMR spectra of BBO-COF 3.

I. TGA Profile

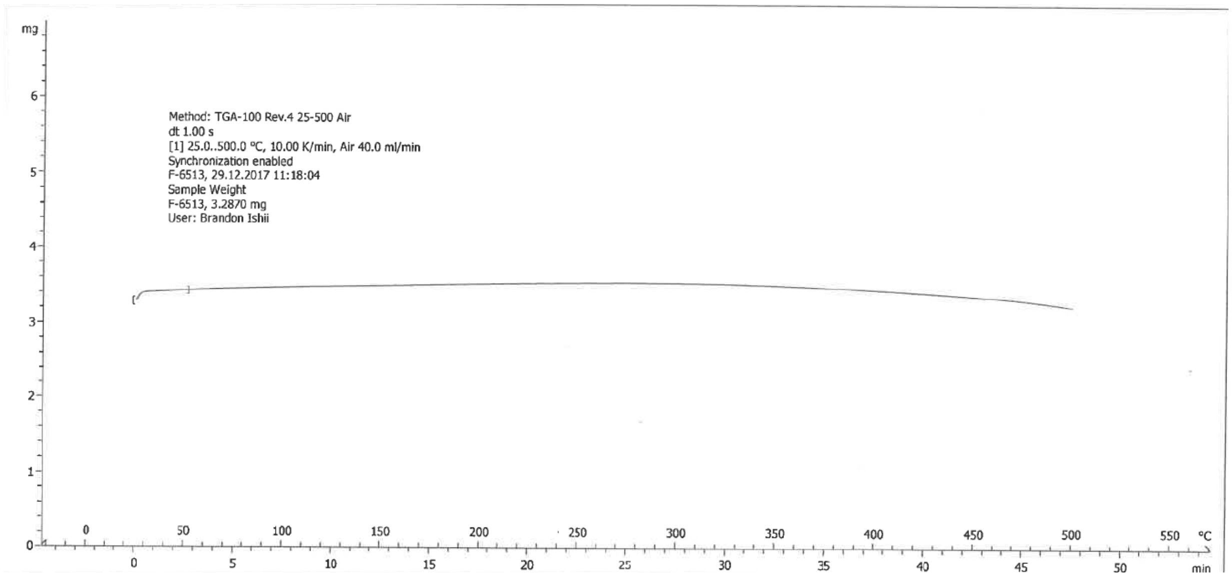

Figure S32. TGA profile for BBO-COF 3

J. Surface Area Analysis

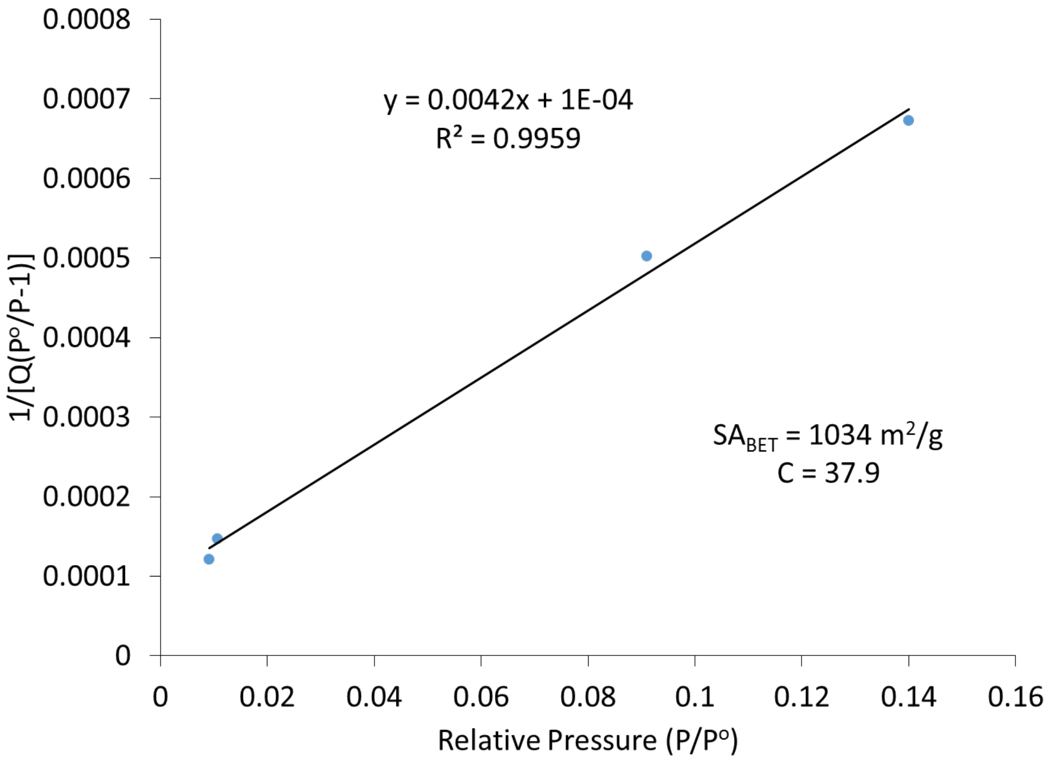

Figure S33. BET surface area plot for BBO-COF 2 synthesized with NaN_3

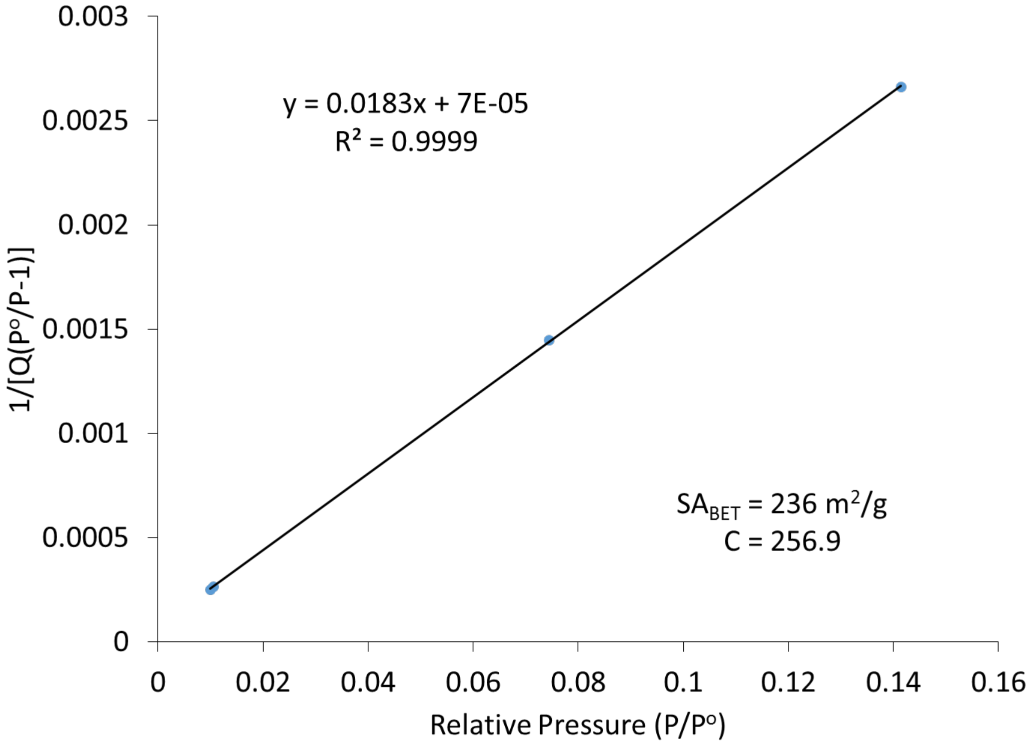

Figure S34. BET surface area plot for BBO-COF 2 synthesized with $NaSCH_3$

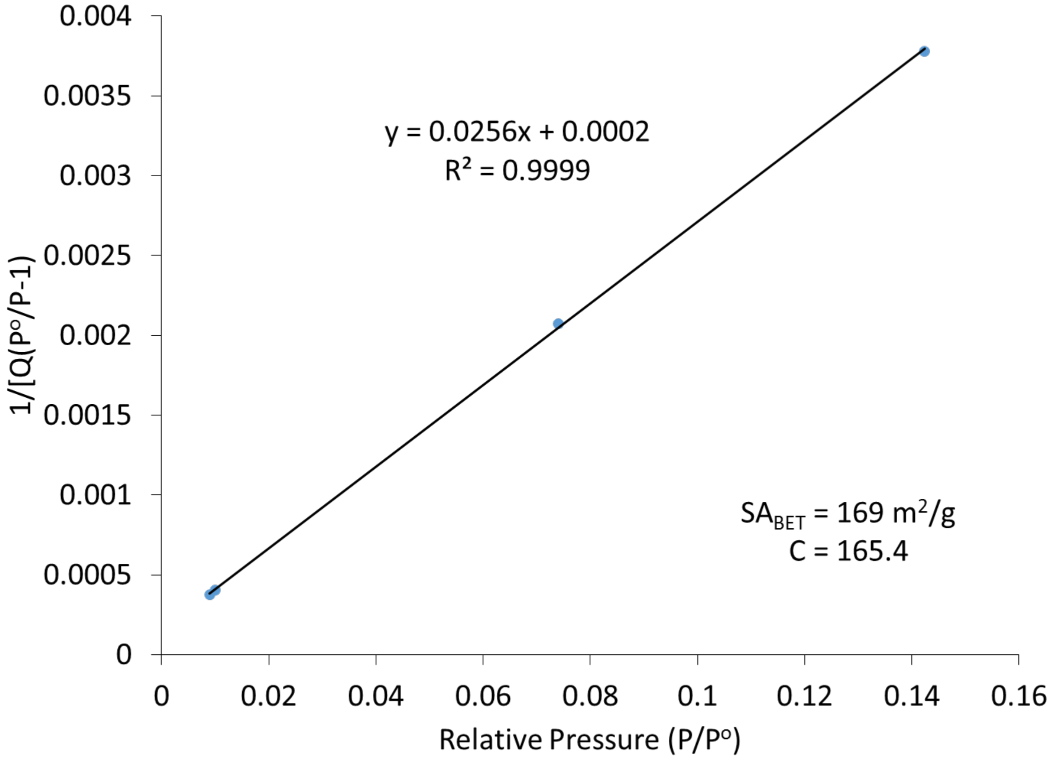

Figure S35. BET surface area plot for BBO-COF 2 synthesized without a catalyst

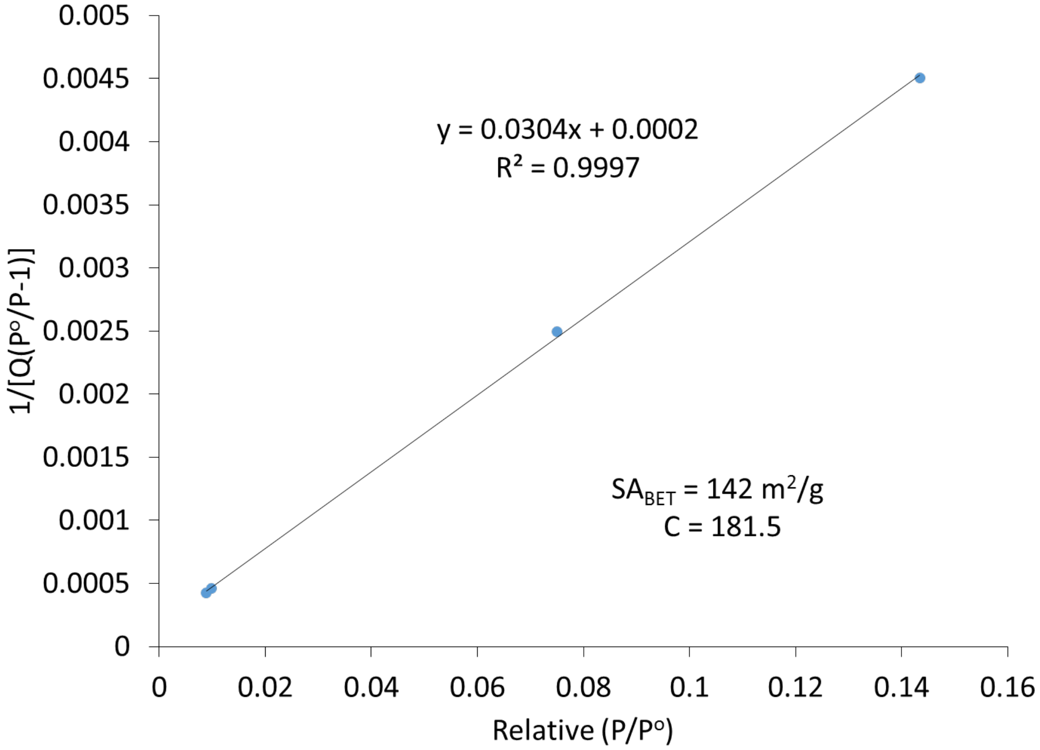

Figure S36. BET surface area plot for BBO-COF 2 deoxygenated

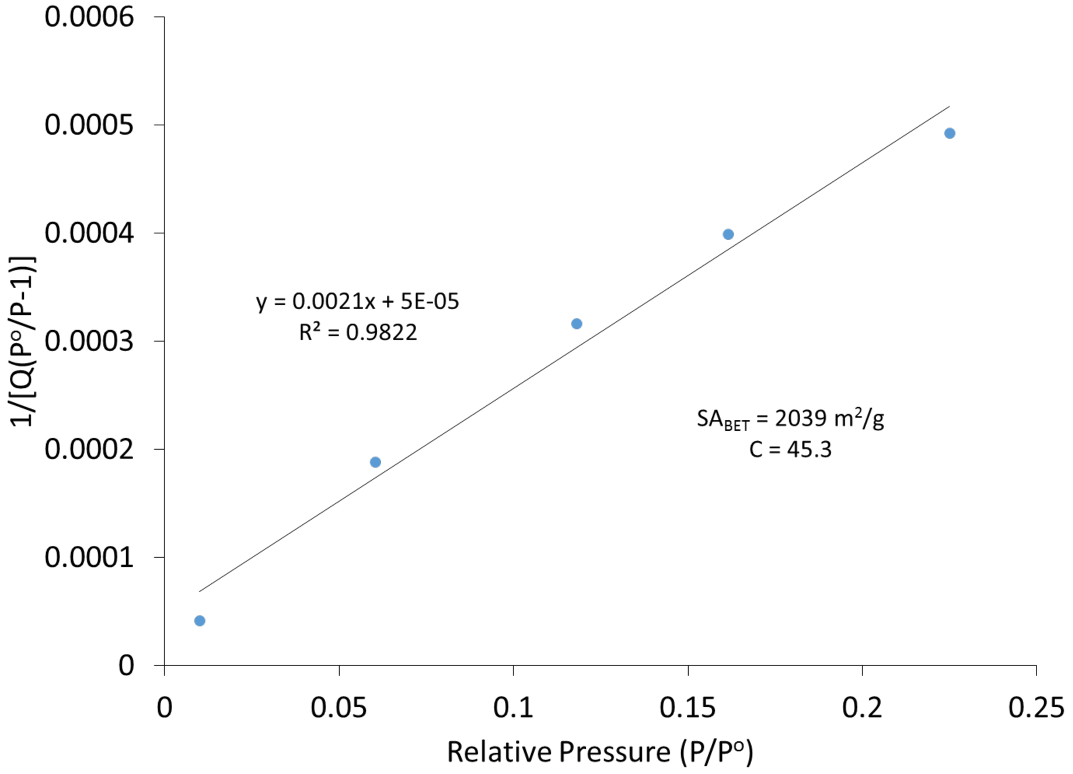

Figure S37. BET surface area plot for BBO-COF 3 synthesized with NaCN

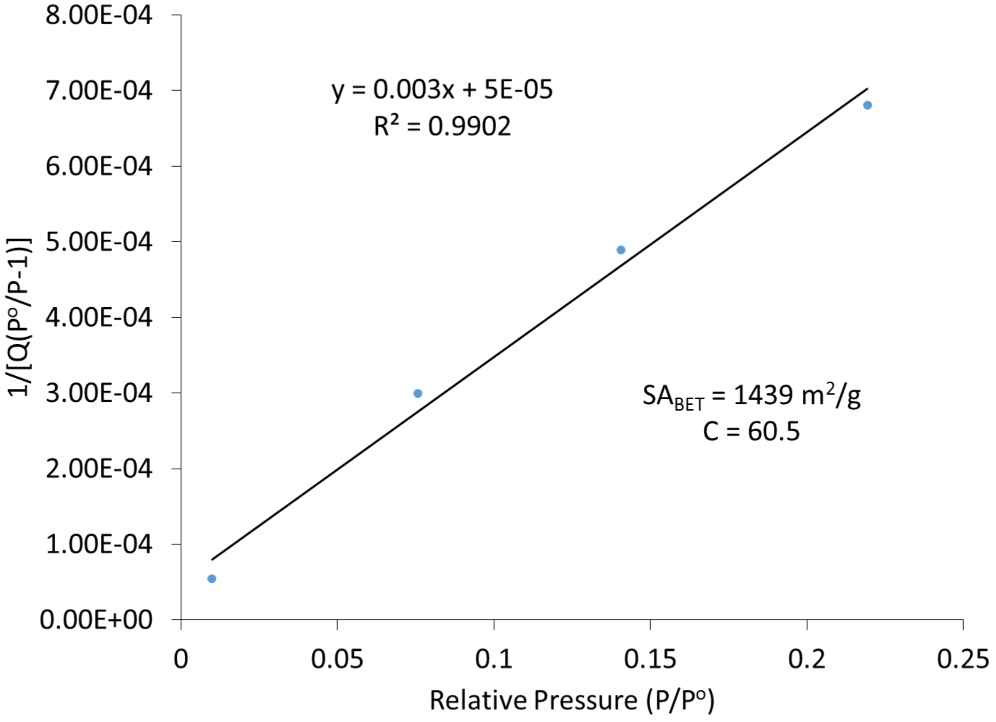

Figure S38. BET surface area plot for BBO-COF 3 synthesized with NaN_3

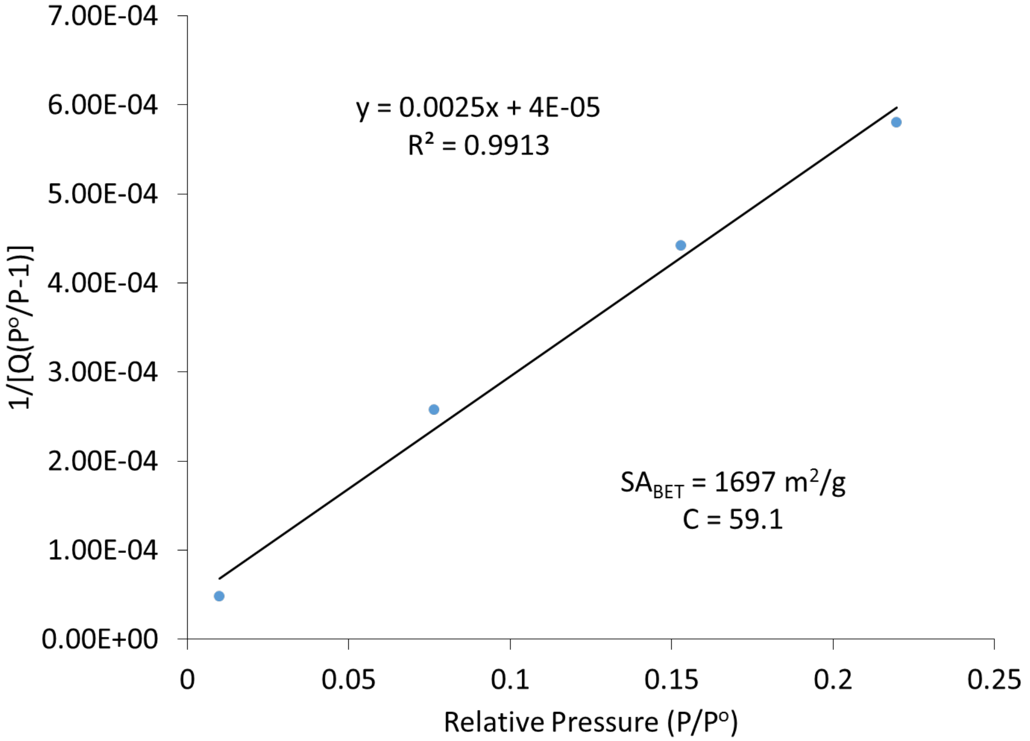

Figure S39. BET surface area plot for BBO-COF 3 synthesized with $NaSCH_3$

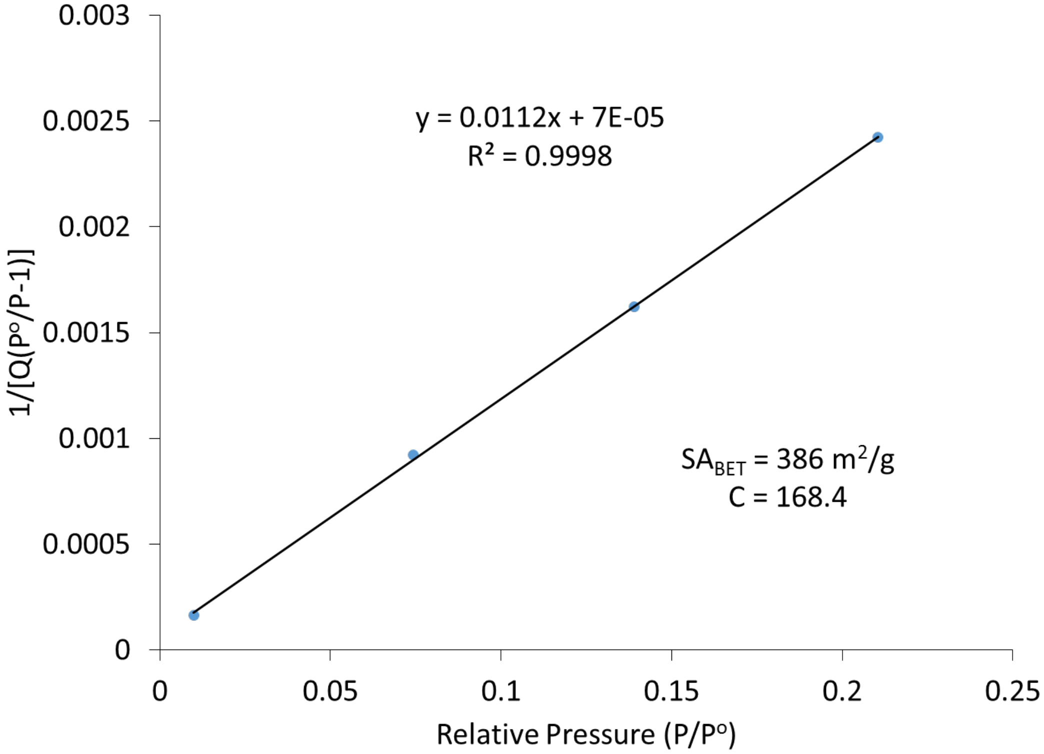

Figure S40. BET surface area plot for BBO-COF 3 synthesized without a catalyst

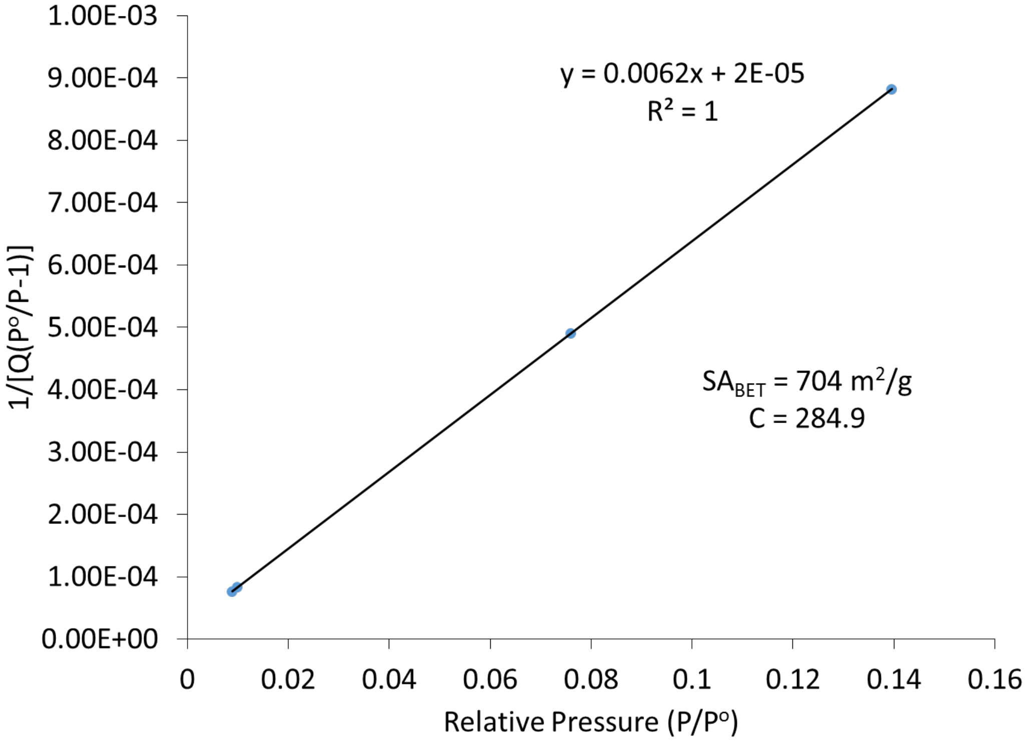

Figure S41. BET surface area plot for BBO-COF 3 after 1 day of heating with NaCN

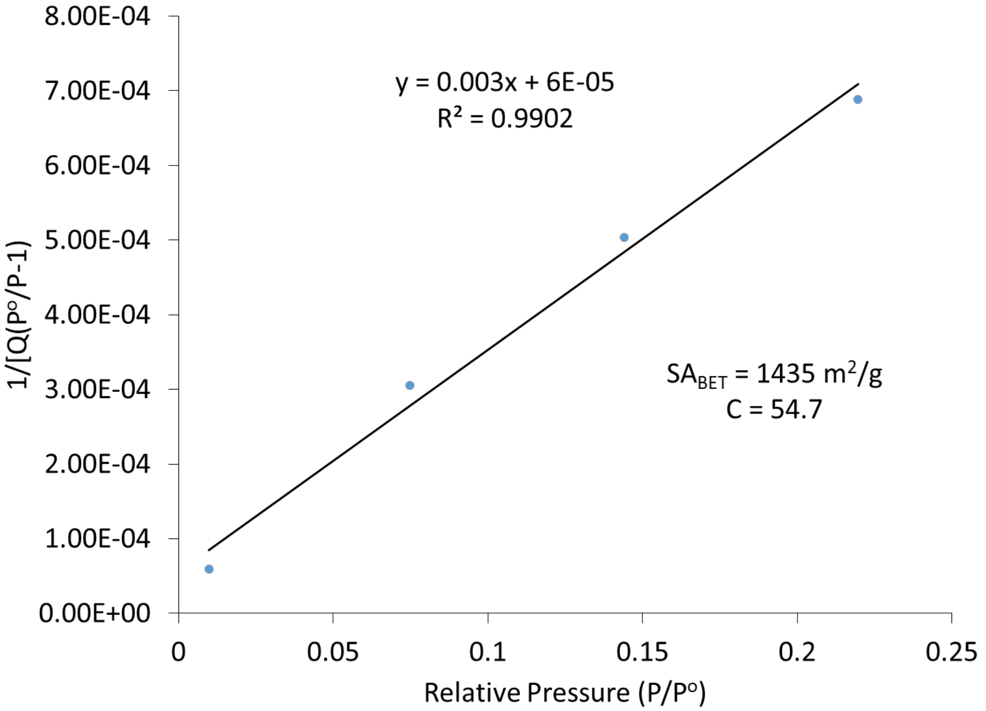

Figure S42. BET surface area plot for BBO-COF 3 after 2 days of heating with NaCN

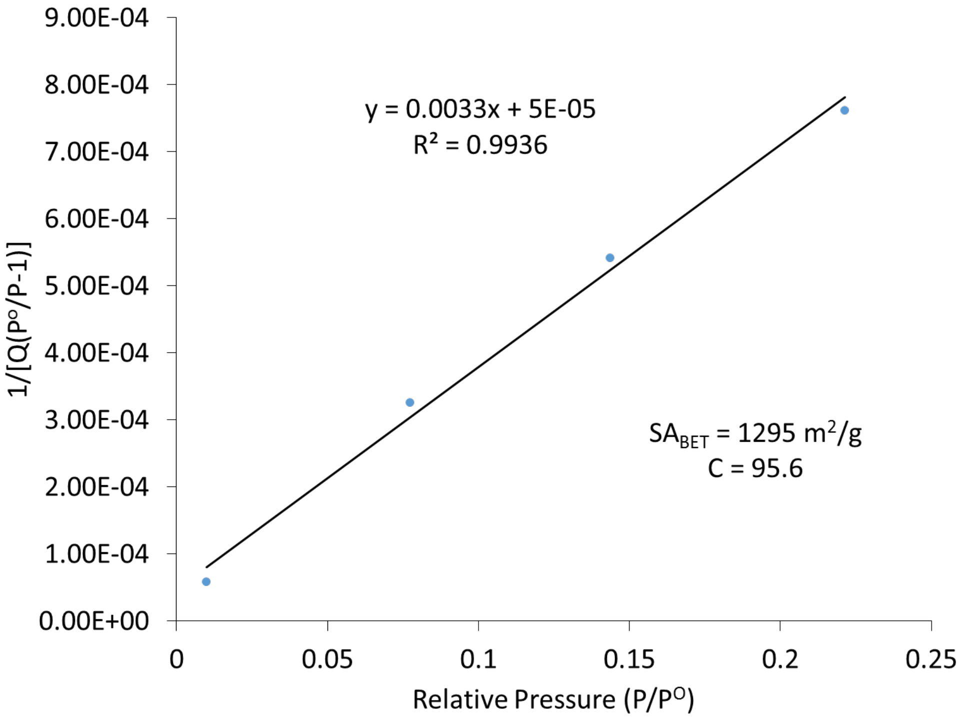

Figure S43. BET surface area plot for BBO-COF 3 after 3 days of heating with NaCN

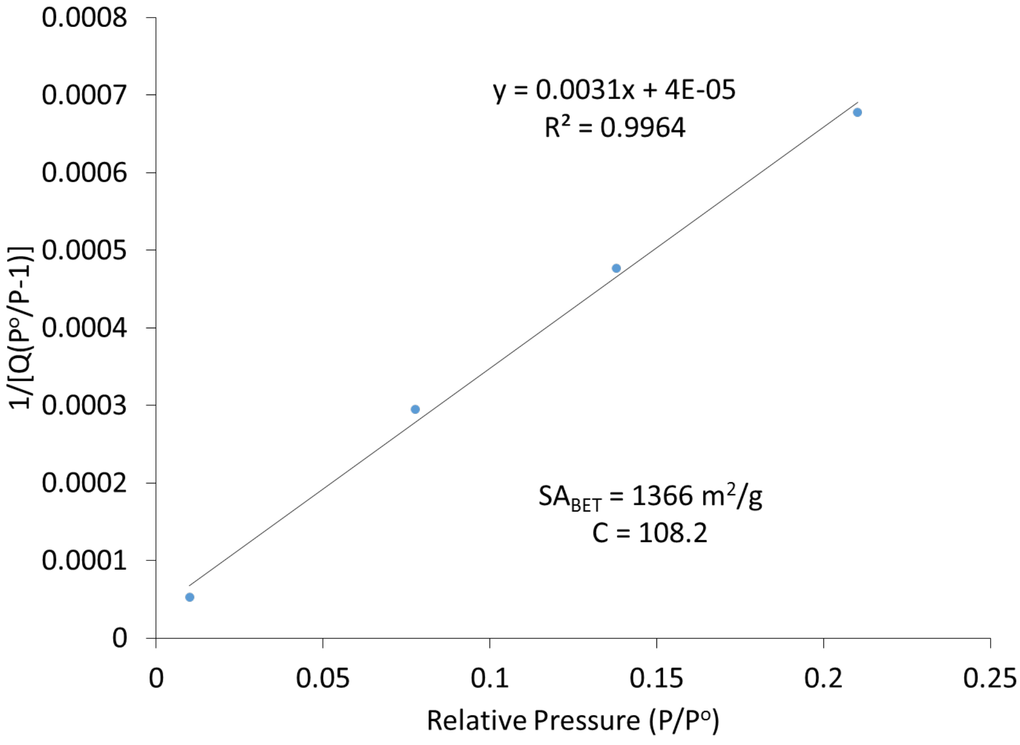

Figure S44. BET surface area plot for BBO-COF 3 deoxygenated

K. Scanning Electron Microscopy (SEM) Images

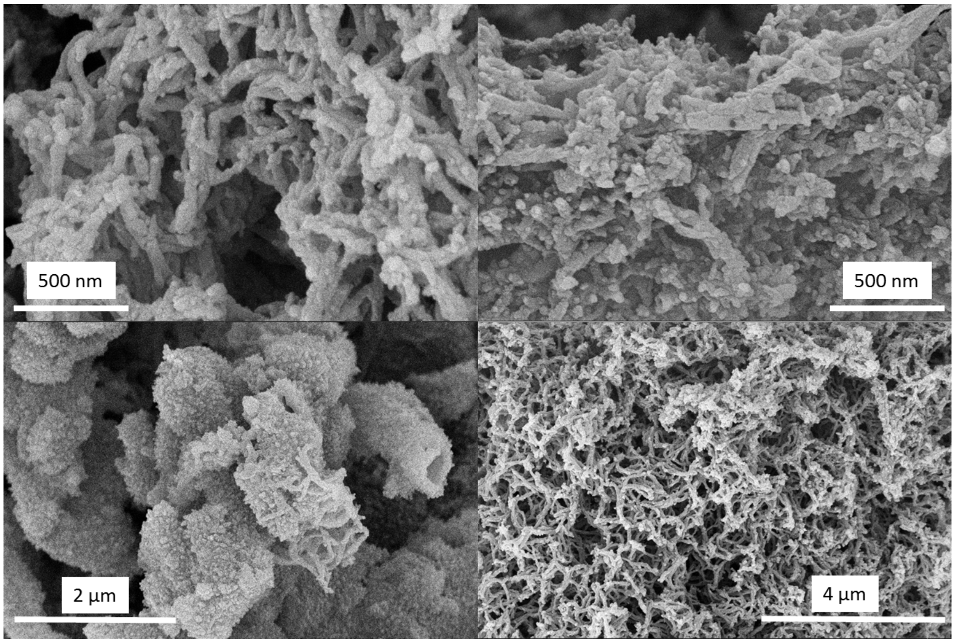

Figure S45. SEM images of BBO-COF 3 at various magnifications

L. Computational Studies

Computational Methods

All calculations were performed using the Gaussian 16 suite of programs.[7] Geometry optimizations and frequency calculations for BBO-COF 2 and BBO-COF 3 monomers were performed with the 6-31G(d)[8] basis set in conjunction with Becke's three-parameter hybrid exchange functional and the LeeYang–Parr correlation functional (B3LYP) density functional theory (DFT) method.[9,10] Rotational barriers were located by torsional scans and the located transitions states were optimized. The nature of all stationary points, either minima or transition states, was confirmed by calculating the vibrational frequencies at the corresponding level of theory. Minima were characterized by the absence of any imaginary vibrational frequencies, while transition states possessed only one imaginary vibrational frequency.

In order to support the presence of a captodative effect promoting radical formation and subsequent cyclization of benzobisoxazole precursors computational methods were employed to evaluate the thermodynamic and molecular properties of these substrates. Both the anionic starting materials and corresponding radical anions were examined to assess bond dissociation energies, spin distribution, and charge distribution. Spin distribution and charge

distribution were quantified using natural population analysis as implemented by nbo version 3.1 in the Gaussian 16 suite of programs.[11] Due to the conformational flexibility of these substrates mixed torsional/Low-mode sampling Monte Carlo conformational analyses were performed on all starting anionic compounds with the OPLS3 forcefield[12] using the MacroModel package of the Schrodinger suite of programs.[13] The resulting structures were further refined by reoptimization and frequency calculations conformation at the B3LYP/6-31+G* level of theory.[14] In order to calculate the geometry of the radical anions, the labile hydrogen atom was abstracted from the anionic starting material *in silico* and the structure optimized for a final time. BDEs were calculated by comparing the energy of the starting anion to the sum of the energies of the product radical and hydrogen atom. Spin density contour plots were produced using the cubegen utility of the Gaussian 16 suite of programs.

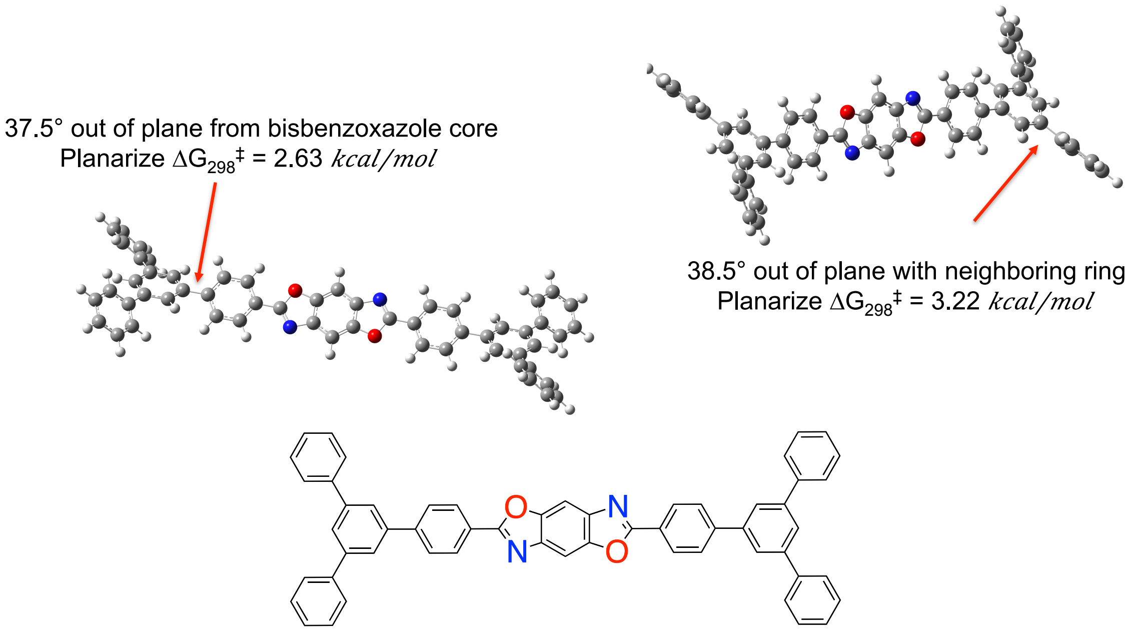

Figure S46. Benzobisoxazole modeling representative of BBO–COF 2

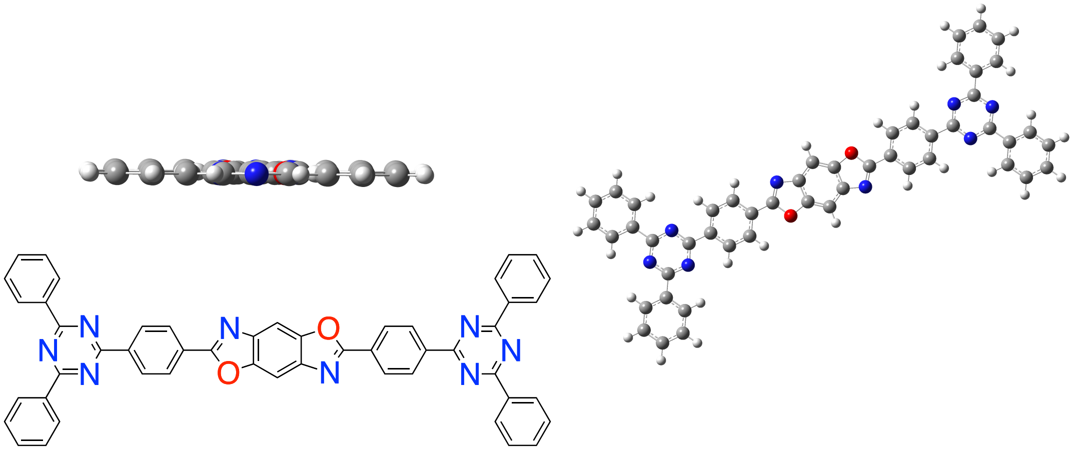

Figure S47. Benzobisoxazole modelling representative of BBO–COF 3

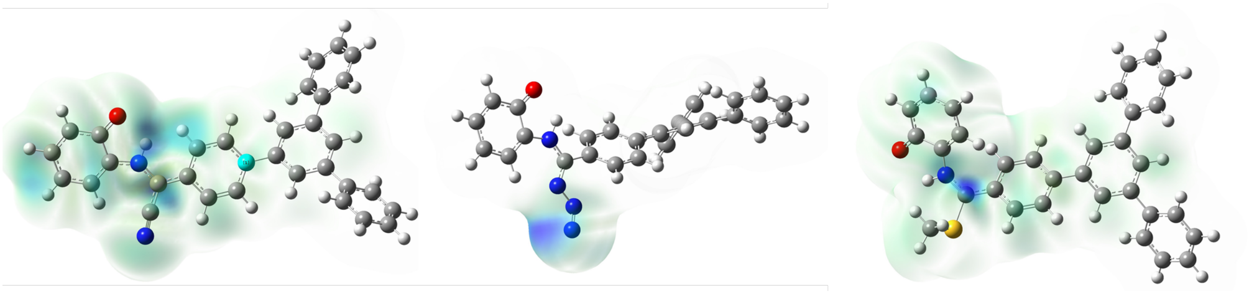

Figure S48. Spin density maps for BBO-COF 2 with NaCN (left), NaN_3 (center), and $NaSCH_3$ (right)

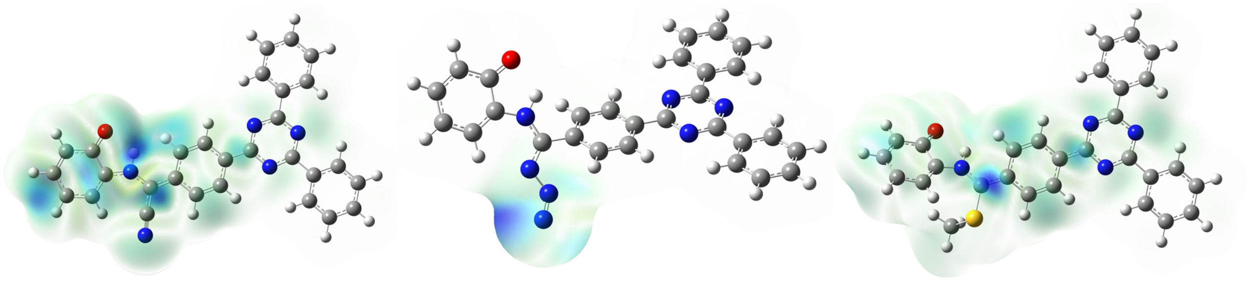

Figure S49. Spin density maps for BBO-COF 3 with NaCN (left), NaN_3 (center), and $NaSCH_3$ (right)

Data

Optimized XYZ coordinates, bottom of the well energies, $<S^2>$ values, and unscaled vibrational frequencies reported in cm^{-1} are supplied for all species calculated at the B3LYP/6-31+G* level of theory unless otherwise specified. Summary spin density and charge distribution calculated by natural population analysis are also provided.

NPA Spin Distribution Summary

Table S18. BBO–COF 2 formation with NaN_3: Before H atom abstraction

Energy: –1489.44615258 hartrees

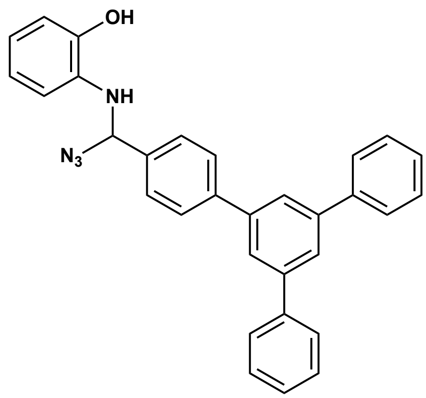

C	8.82430	0.86494	-0.92082
C	8.38094	-0.45908	-0.79385
C	7.03292	-0.69679	-0.47065
C	6.15781	0.37031	-0.29690
C	6.57911	1.75931	-0.40610
C	7.95777	1.94428	-0.73028
N	4.78907	0.25978	-0.01194
C	4.06974	-0.88305	0.19190
N	4.39450	-1.54971	1.71971
C	2.58234	-0.68658	0.15423
C	1.78244	-1.47119	-0.68647
C	0.40011	-1.28880	-0.73743
C	-0.23102	-0.31428	0.05304
C	0.57699	0.46216	0.90587
C	1.95471	0.27460	0.96321
C	-1.70091	-0.10825	-0.00978
C	-2.58027	-1.19719	-0.12859
C	-3.97082	-1.01600	-0.17747
C	-4.48468	0.28792	-0.10202
C	-3.63414	1.39800	0.01808
C	-2.24869	1.18288	0.06216
O	5.72436	2.69660	-0.21433
N	4.27536	-2.76286	1.71743
N	4.16645	-3.91570	1.71047
H	9.86911	1.05874	-1.16693
H	9.06586	-1.29396	-0.92436
H	6.68453	-1.72079	-0.34153
H	8.31432	2.96840	-0.82674

H	4.39946	1.18880	0.16841
H	4.39401	-1.70222	-0.45989
H	2.24788	-2.22138	-1.32154
H	-0.19028	-1.89101	-1.42376
H	0.11548	1.20045	1.55700
H	2.55024	0.86450	1.65456
H	-2.17523	-2.20503	-0.13135
H	-5.55608	0.44435	-0.19197
H	-1.58045	2.03402	0.15441
C	-4.88414	-2.18352	-0.30681
C	-4.56543	-3.26239	-1.15100
C	-6.09223	-2.24199	0.41115
C	-5.42174	-4.35828	-1.27339
H	-3.64776	-3.23096	-1.73233
C	-6.95066	-3.33632	0.28910
H	-6.34825	-1.43303	1.09049
C	-6.61964	-4.40079	-0.55426
H	-5.15469	-5.17699	-1.93745
H	-7.87447	-3.36166	0.86255
H	-7.28629	-5.25450	-0.64852
C	-4.19027	2.77708	0.07583
C	-5.35652	3.05634	0.81081
C	-3.56494	3.84013	-0.60034
C	-5.88035	4.34930	0.86670
H	-5.84223	2.25666	1.36417
C	-4.08640	5.13402	-0.54401
H	-2.67378	3.64731	-1.19157
C	-5.24746	5.39543	0.18919
H	-6.77807	4.54103	1.45003
H	-3.58647	5.93754	-1.07952
H	-5.65267	6.40332	0.23420

Table S19. Vibrational frequencies for BBO–COF 2 formation with NaN_3: Before H atom abstraction

10	16	26	26	34	41
42	45	53	56	59	89
114	128	141	146	155	172
204	225	234	239	270	280
288	295	323	353	374	404
408	418	419	421	454	462
508	517	520	559	570	581
588	589	597	624	626	628

634	641	645	658	675	685
702	708	709	709	722	737
752	771	781	794	827	835
839	847	854	857	858	870
876	897	903	917	922	925
933	937	966	979	979	981
997	998	1009	1015	1017	1026
1040	1047	1054	1066	1107	1108
1109	1119	1121	1145	1167	1189
1189	1191	1213	1214	1218	1230
1242	1266	1268	1272	1296	1304
1321	1326	1329	1342	1345	1351
1360	1365	1371	1372	1377	1419
1440	1451	1471	1493	1499	1504
1527	1532	1539	1540	1558	1590
1609	1623	1624	1631	1637	1638
1652	1653	1659	2149	3057	3131
3150	3169	3173	3173	3177	3180
3181	3182	3184	3191	3192	3194
3195	3198	3199	3202	3203	3206

Table S20. BBO–COF 2 formation with NaN_3: Radical intermediate

Energy: –1488.84825212 hartrees
$\langle S^2 \rangle = 0.7570$

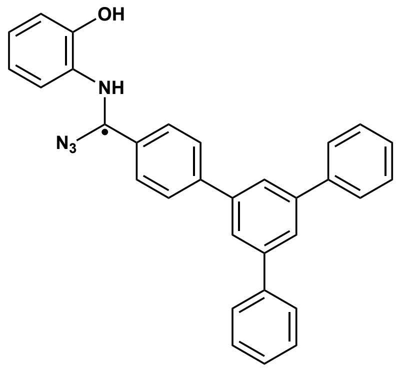

C	8.74464	0.11658	-1.63147
C	8.55171	0.07672	-0.23977
C	7.24976	0.01208	0.27420
C	6.16489	-0.00760	-0.60390
C	6.31955	0.02585	-2.05282
C	7.66951	0.09289	-2.51670
N	4.80080	-0.05214	-0.26891
C	4.12874	-0.09727	0.88914
N	4.79244	-0.17583	2.04268
C	2.63977	-0.06896	0.73542
C	2.03602	0.87003	-0.11588
C	0.65270	0.89073	-0.29320
C	-0.17628	-0.03130	0.36632
C	0.43452	-0.97577	1.21221
C	1.81396	-0.99117	1.39907
O	5.27950	-0.00189	-2.80352

N	3.93841	-0.02231	3.15378
N	4.28252	-0.11647	4.28584
H	9.75904	0.16534	-2.02934
H	9.40144	0.09311	0.43892
H	7.07092	-0.02506	1.34259
H	7.82794	0.12144	-3.59296
H	4.27942	-0.08812	-1.15824
H	2.65487	1.59067	-0.64341
H	0.21722	1.61670	-0.97532
H	-0.18084	-1.69037	1.75333
H	2.25745	-1.72359	2.06500
C	-1.64989	-0.01202	0.17600
C	-2.35006	1.20237	0.09387
C	-2.37866	-1.20788	0.07316
C	-3.74084	1.23760	-0.08672
H	-1.79908	2.13514	0.17118
C	-3.76989	-1.20710	-0.10698
H	-1.85023	-2.15476	0.13499
C	-4.43860	0.02411	-0.18535
H	-5.51593	0.03807	-0.32501
C	-4.46312	2.53617	-0.16909
C	-4.11955	3.60941	0.67260
C	-5.50876	2.72662	-1.09033
C	-4.79617	4.82829	0.59616
H	-3.32879	3.47990	1.40678
C	-6.18742	3.94447	-1.16675
H	-5.77445	1.92079	-1.76970
C	-5.83443	5.00206	-0.32357
H	-4.51499	5.64094	1.26176
H	-6.98593	4.07008	-1.89438
H	-6.36068	5.95153	-0.38414
C	-4.52194	-2.48670	-0.21568
C	-5.77234	-2.64760	0.40801
C	-4.00256	-3.57096	-0.94572
C	-6.47867	-3.84764	0.30499
H	-6.18215	-1.83282	0.99940
C	-4.70703	-4.77203	-1.04830
H	-3.04823	-3.46371	-1.45446
C	-5.94935	-4.91656	-0.42391
H	-7.43943	-3.95040	0.80427
H	-4.28643	-5.59330	-1.62367
H	-6.49775	-5.85202	-0.50305

Table S21. Vibrational frequencies for BBO–COF 2 formation with NaN_3: Radical intermediate

11	18	26	41	43	46
51	54	57	71	96	116
119	120	151	155	188	198
210	228	236	258	269	280
297	300	338	345	373	393
418	418	420	447	461	470
502	507	532	537	568	581
584	621	623	628	632	637
640	647	650	679	696	709
710	724	727	743	748	762
774	776	811	836	836	843
849	855	858	858	884	894
898	918	919	930	935	935
939	945	975	979	979	980
997	998	1009	1015	1016	1026
1040	1043	1054	1066	1107	1108
1109	1119	1121	1148	1149	1171
1189	1189	1214	1214	1216	1224
1264	1267	1268	1296	1305	1325
1326	1338	1344	1352	1366	1369
1372	1372	1412	1432	1451	1465
1485	1492	1499	1510	1538	1540
1542	1557	1572	1602	1607	1623
1625	1635	1638	1639	1652	1653
1656	1788	3132	3171	3172	3173
3178	3181	3181	3183	3184	3190
3192	3194	3197	3199	3201	3202
3205	3205	3206	3222	3230	3308

Table S22. NPA Charge for BBO–COF 2 formation with NaN_3: Radical intermediate

Azide	Alpha Charge	Beta Charge	Charge Sum
C 1	-0.12342	-0.12537	-0.24879
C 2	-0.16701	-0.16737	-0.33438

C 3	-0.12801	-0.12815	-0.25616
C 4	0.01629	0.01768	0.03397
C 5	0.18171	0.18014	0.36185
C 6	-0.17329	-0.17412	-0.34741
N 7	-0.27434	-0.28197	-0.55631
C 8	0.21866	0.21002	0.42868
N 9	-0.29427	-0.14355	-0.43782
C 10	-0.0463	-0.04013	-0.08643
C 11	-0.10835	-0.10604	-0.21439
C 12	-0.11129	-0.10972	-0.22101
C 13	-0.03586	-0.03479	-0.07065
C 14	-0.11583	-0.11115	-0.22698
C 15	-0.09542	-0.0964	-0.19182
O 16	-0.38787	-0.38911	-0.77698
N 17	-0.11763	0.04495	-0.07268
N 18	-0.41077	0.2801	-0.13067
H 19	0.10804	0.10814	0.21618
H 20	0.11019	0.11023	0.22042
H 21	0.12931	0.12902	0.25833
H 22	0.11238	0.11241	0.22479
H 23	0.2306	0.23201	0.46261
H 24	0.12937	0.12935	0.25872
H 25	0.12002	0.11997	0.23999
H 26	0.11921	0.11911	0.23832
H 27	0.1288	0.12893	0.25773
C 28	-0.00842	-0.00842	-0.01684
C 29	-0.1029	-0.10271	-0.20561
C 30	-0.10304	-0.10269	-0.20573
C 31	-0.02547	-0.02552	-0.05099
H 32	0.12235	0.12235	0.2447
C 33	-0.02546	-0.02558	-0.05104
H 34	0.12226	0.12225	0.24451
C 35	-0.10568	-0.1053	-0.21098
H 36	0.11897	0.11896	0.23793
C 37	-0.02267	-0.02266	-0.04533
C 38	-0.11091	-0.11091	-0.22182
C 39	-0.11246	-0.11246	-0.22492
C 40	-0.11854	-0.11854	-0.23708
H 41	0.12342	0.12342	0.24684

C 42	-0.11967	-0.11967	-0.23934
H 43	0.12136	0.12136	0.24272
C 44	-0.12414	-0.12414	-0.24828
H 45	0.12085	0.12085	0.2417
H 46	0.11967	0.11967	0.23934
H 47	0.11934	0.11934	0.23868
C 48	-0.02269	-0.02268	-0.04537
C 49	-0.11263	-0.11264	-0.22527
C 50	-0.11082	-0.11084	-0.22166
C 51	-0.11983	-0.11982	-0.23965
H 52	0.12121	0.12121	0.24242
C 53	-0.11845	-0.11845	-0.2369
H 54	0.12373	0.12373	0.24746
C 55	-0.12419	-0.12421	-0.2484
H 56	0.11956	0.11956	0.23912
H 57	0.12102	0.12102	0.24204
H 58	0.11933	0.11933	0.23866

Table S23. NPA Spin for BBO–COF 2 formation with NaN_3: Radical intermediate

Azide	Alpha Spin	Beta Spin	Alpha – Beta Difference Density
C 1	3.12342	3.12537	-0.00195
C 2	3.16701	3.16737	-0.00036
C 3	3.12801	3.12815	-0.00014
C 4	2.98371	2.98232	0.00139
C 5	2.81829	2.81986	-0.00157
C 6	3.17329	3.17412	-0.00083
N 7	3.77434	3.78197	-0.00763
C 8	2.78134	2.78998	-0.00864
N 9	3.79427	3.64355	0.15072
C 10	3.0463	3.04013	0.00617
C 11	3.10835	3.10604	0.00231
C 12	3.11129	3.10972	0.00157
C 13	3.03586	3.03479	0.00107
C 14	3.11583	3.11115	0.00468
C 15	3.09542	3.0964	-0.00098
O 16	4.38787	4.38911	-0.00124

N	17	3.61763	3.45505	0.16258
N	18	3.91077	3.2199	0.69087
H	19	0.39196	0.39186	1E-04
H	20	0.38981	0.38977	4E-05
H	21	0.37069	0.37098	-0.00029
H	22	0.38762	0.38759	3E-05
H	23	0.2694	0.26799	0.00141
H	24	0.37063	0.37065	-2E-05
H	25	0.37998	0.38003	-5E-05
H	26	0.38079	0.38089	-1E-04
H	27	0.3712	0.37107	0.00013
C	28	3.00842	3.00842	0
C	29	3.1029	3.10271	0.00019
C	30	3.10304	3.10269	0.00035
C	31	3.02547	3.02552	-5E-05
H	32	0.37765	0.37765	0
C	33	3.02546	3.02558	-0.00012
H	34	0.37774	0.37775	-1E-05
C	35	3.10568	3.1053	0.00038
H	36	0.38103	0.38104	-1E-05
C	37	3.02267	3.02266	1E-05
C	38	3.11091	3.11091	0
C	39	3.11246	3.11246	0
C	40	3.11854	3.11854	0
H	41	0.37658	0.37658	0
C	42	3.11967	3.11967	0
H	43	0.37864	0.37864	0
C	44	3.12414	3.12414	0
H	45	0.37915	0.37915	0
H	46	0.38033	0.38033	0
H	47	0.38066	0.38066	0
C	48	3.02269	3.02268	1E-05
C	49	3.11263	3.11264	-1E-05
C	50	3.11082	3.11084	-2E-05
C	51	3.11983	3.11982	1E-05
H	52	0.37879	0.37879	0
C	53	3.11845	3.11845	0
H	54	0.37627	0.37627	0
C	55	3.12419	3.12421	-2E-05

H 56	0.38044	0.38044	0
H 57	0.37898	0.37898	0
H 58	0.38067	0.38067	0

Table S24. BBO–COF 3 formation with NaN_3: Before H atom abstraction

Energy: –1537.59144639 hartrees

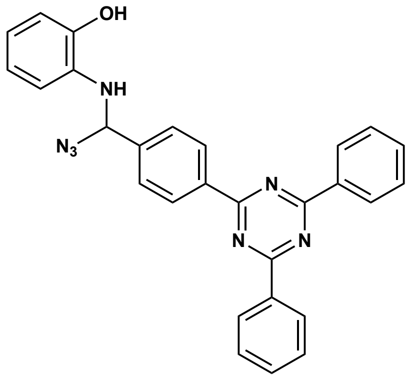

C	8.68730	0.18891	-1.50234
C	8.26594	-0.79915	-0.59847
C	6.93704	-0.78764	-0.14372
C	6.05085	0.18558	-0.59878
C	6.45100	1.23664	-1.52665
C	7.81482	1.17995	-1.95237
N	4.70240	0.27942	-0.24566
O	5.59491	2.11473	-1.89319
C	4.00381	-0.54428	0.60240
N	4.36046	-0.20631	2.19585
C	2.51557	-0.41568	0.46901
C	1.72079	-1.55523	0.26660
C	0.33881	-1.45225	0.14324
C	-0.29537	-0.19871	0.22560
C	0.50204	0.94447	0.43998
C	1.88035	0.83628	0.56677
C	-1.75615	-0.08456	0.09410
N	-2.47237	-1.21038	-0.10777
C	-3.79437	-1.05574	-0.22371
N	-4.43405	0.12477	-0.15267
C	-3.64365	1.19394	0.05061
N	-2.31472	1.14019	0.17927
N	4.34524	-1.20564	2.89716
N	4.33739	-2.14916	3.56510
H	9.71899	0.18635	-1.85529
H	8.95804	-1.55642	-0.23737
H	6.61140	-1.53437	0.57903
H	8.14998	1.94423	-2.65103
H	4.29328	1.13161	-0.63450
H	4.32777	-1.58811	0.51630
H	2.19419	-2.53179	0.19784
H	-0.26368	-2.33920	-0.01914
H	0.02233	1.91426	0.51245
H	2.47472	1.72597	0.75629
C	-4.29494	2.52873	0.13839

C	-3.52179	3.68601	0.32905
C	-5.69024	2.65036	0.03155
C	-4.13224	4.93754	0.41089
H	-2.44475	3.58866	0.40936
C	-6.29864	3.90318	0.11464
H	-6.28401	1.75497	-0.11615
C	-5.52239	5.05106	0.30451
H	-3.52190	5.82532	0.55699
H	-7.37991	3.98412	0.03106
H	-5.99714	6.02742	0.36859
C	-4.61782	-2.27516	-0.44750
C	-4.01094	-3.53979	-0.52153
C	-6.01195	-2.17859	-0.58838
C	-4.78275	-4.68252	-0.73196
H	-2.93428	-3.61033	-0.41273
C	-6.78197	-3.32290	-0.79921
H	-6.47697	-1.20056	-0.53079
C	-6.17073	-4.57878	-0.87170
H	-4.30030	-5.65533	-0.78734
H	-7.86049	-3.23458	-0.90704
H	-6.77147	-5.47034	-1.03605

Table S25. Vibrational frequencies for BBO–COF 3 formation with NaN_3: Before H atom abstraction

9	14	21	23	28	29
36	43	51	63	70	84
109	129	145	146	168	181
201	217	230	239	247	267
293	302	315	356	397	418
419	420	441	444	459	460
488	516	522	561	568	583
589	593	610	631	632	648
653	660	664	688	689	692
708	710	711	713	739	754
789	798	812	829	850	853
856	857	864	866	873	876
880	905	930	954	956	982
994	995	997	1007	1007	1009
1016	1018	1032	1047	1047	1051

1076	1103	1104	1112	1141	1166
1173	1174	1188	1188	1192	1204
1205	1213	1232	1236	1248	1273
1305	1318	1331	1332	1344	1352
1360	1361	1363	1379	1392	1402
1414	1435	1453	1481	1484	1504
1511	1519	1527	1536	1538	1553
1568	1587	1608	1629	1632	1636
1652	1652	1656	2158	3051	3136
3154	3173	3174	3175	3182	3185
3187	3187	3199	3199	3200	3227
3228	3229	3229	3231	3232	3455

Table S26. BBO–COF 3 formation with NaN_3: Radical intermediate

Energy: –1536.99264920 hartrees
$\langle S^2 \rangle = 0.7579$

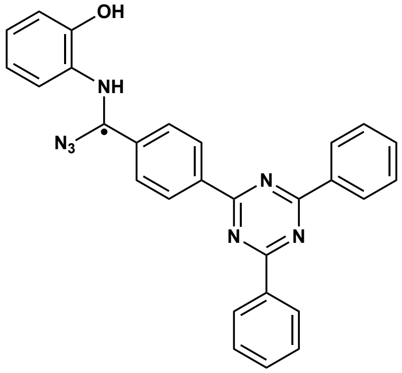

C	8.69904	1.09551	-0.97149
C	8.47070	-0.00188	-0.12048
C	7.15823	-0.39414	0.16529
C	6.09199	0.31420	-0.39530
C	6.28402	1.45295	-1.28775
C	7.64695	1.80572	-1.54021
N	4.72366	0.07754	-0.20594
C	4.02658	-0.83654	0.48712
N	4.67019	-1.80139	1.14393
C	2.54482	-0.66178	0.40965
C	1.97614	0.61793	0.54986
C	0.60112	0.80402	0.44967
C	-0.25317	-0.28512	0.20259

C	0.31545	-1.56553	0.05463
C	1.68796	-1.75040	0.16090
O	5.26477	2.05499	-1.77622
N	3.80634	-2.53754	1.98090
N	4.13012	-3.48851	2.61223
H	9.72390	1.39552	-1.19242
H	9.30487	-0.54750	0.31439
H	6.95401	-1.23876	0.81272
H	7.83037	2.65073	-2.20053
H	4.21866	0.75792	-0.79317
H	2.61839	1.47026	0.75257
H	0.17608	1.79474	0.56611
H	-0.33348	-2.41080	-0.14583
H	2.10714	-2.74315	0.04107
C	-1.70986	-0.09085	0.09882
C	-3.52865	1.28280	0.13978
C	-3.79988	-0.94299	-0.22019
N	-2.48474	-1.16874	-0.13528
N	-2.20184	1.15567	0.24253
N	-4.37523	0.26428	-0.09030
C	-4.68793	-2.10940	-0.47582
C	-4.14714	-3.39943	-0.60333
C	-6.07690	-1.93676	-0.59255
C	-4.97929	-4.49319	-0.84235
H	-3.07435	-3.52863	-0.51206
C	-6.90709	-3.03206	-0.83239
H	-6.49070	-0.93922	-0.49363
C	-6.36163	-4.31386	-0.95795
H	-4.54815	-5.48664	-0.93824
H	-7.98092	-2.88549	-0.92145
H	-7.00932	-5.16733	-1.14454
C	-4.10819	2.64477	0.29024
C	-3.27212	3.75239	0.50763
C	-5.49694	2.84159	0.21612
C	-3.81493	5.02974	0.64738
H	-2.20037	3.59687	0.56283
C	-6.03755	4.11991	0.35744
H	-6.13923	1.98415	0.04769
C	-5.19889	5.21828	0.57327
H	-3.15662	5.87884	0.81337
H	-7.11439	4.25953	0.29862
H	-5.62082	6.21468	0.68233

Table S27. Vibrational frequencies for BBO–COF 3 formation with NaN_3: Radical intermediate

9	19	27	28	30	43
53	58	65	72	89	106
109	138	154	175	177	204
212	216	230	238	258	265
292	296	335	346	410	419
420	421	444	459	461	470
486	506	528	537	565	586
608	625	631	632	641	658
666	682	688	690	701	710
712	730	746	753	767	789
813	814	838	853	856	857
859	865	866	876	884	900
933	937	949	956	957	991
994	996	997	1008	1009	1010
1017	1019	1033	1042	1047	1051
1076	1104	1104	1110	1145	1146
1171	1175	1177	1188	1189	1204
1206	1217	1220	1233	1266	1307
1331	1332	1342	1348	1361	1364
1371	1395	1402	1409	1434	1444
1479	1486	1487	1512	1532	1536
1538	1544	1547	1563	1572	1600
1607	1633	1634	1637	1653	1653
1654	1789	3136	3174	3174	3175
3181	3187	3187	3199	3199	3201
3218	3227	3228	3229	3231	3231

Table S28. NPA charge for BBO–COF 3 formation with NaN_3: Radical intermediate

Azide	Alpha Charge	Beta Charge	Charge Sum
C 1	-0.12234	-0.1244	-0.24674
C 2	-0.16236	-0.16473	-0.32709
C 3	-0.12829	-0.12777	-0.25606
C 4	0.02057	0.02016	0.04073

C 5	0.18342	0.18145	0.36487
C 6	-0.17048	-0.17255	-0.34303
N 7	-0.27128	-0.28065	-0.55193
C 8	0.21533	0.2076	0.42293
N 9	-0.29545	-0.14276	-0.43821
C 10	-0.03617	-0.02973	-0.0659
C 11	-0.11716	-0.11493	-0.23209
C 12	-0.08869	-0.08823	-0.17692
C 13	-0.06188	-0.06029	-0.12217
C 14	-0.09663	-0.08913	-0.18576
C 15	-0.10341	-0.1044	-0.20781
O 16	-0.37968	-0.38389	-0.76357
N 17	-0.11652	0.04441	-0.07211
N 18	-0.40819	0.28217	-0.12602
H 19	0.10894	0.10904	0.21798
H 20	0.111	0.11112	0.22212
H 21	0.13037	0.13007	0.26044
H 22	0.11332	0.11338	0.2267
H 23	0.23053	0.23197	0.4625
H 24	0.12936	0.12934	0.2587
H 25	0.12833	0.1283	0.25663
H 26	0.12772	0.12753	0.25525
H 27	0.12933	0.12945	0.25878
C 28	0.2259	0.22835	0.45425
C 29	0.22378	0.22416	0.44794
C 30	0.22407	0.22385	0.44792
N 31	-0.25777	-0.25662	-0.51439
N 32	-0.25668	-0.25627	-0.51295
N 33	-0.26678	-0.26435	-0.53113
C 34	-0.04396	-0.04391	-0.08787
C 35	-0.09769	-0.09775	-0.19544
C 36	-0.10031	-0.10025	-0.20056
C 37	-0.12285	-0.12277	-0.24562
H 38	0.13264	0.13264	0.26528
C 39	-0.12428	-0.12426	-0.24854
H 40	0.12959	0.12959	0.25918
C 41	-0.11475	-0.11474	-0.22949
H 42	0.12136	0.12136	0.24272
H 43	0.11963	0.11963	0.23926

H 44	0.11929	0.11929	0.23858
C 45	-0.04422	-0.04426	-0.08848
C 46	-0.09744	-0.09738	-0.19482
C 47	-0.10053	-0.10028	-0.20081
C 48	-0.12256	-0.12256	-0.24512
H 49	0.13286	0.13286	0.26572
C 50	-0.12432	-0.12439	-0.24871
H 51	0.12941	0.1294	0.25881
C 52	-0.11467	-0.11448	-0.22915
H 53	0.12172	0.12172	0.24344
H 54	0.11956	0.11956	0.23912
H 55	0.11936	0.11935	0.23871

Table S29. NPA spin for BBO–COF 3 formation with NaN_3: Radical intermediate

Azide	Alpha Spin	Beta Spin	Alpha – Beta Difference Density
C 1	3.12234	3.1244	-0.00206
C 2	3.16236	3.16473	-0.00237
C 3	3.12829	3.12777	0.00052
C 4	2.97943	2.97984	-0.00041
C 5	2.81658	2.81855	-0.00197
C 6	3.17048	3.17255	-0.00207
N 7	3.77128	3.78065	-0.00937
C 8	2.78467	2.7924	-0.00773
N 9	3.79545	3.64276	0.15269
C 10	3.03617	3.02973	0.00644
C 11	3.11716	3.11493	0.00223
C 12	3.08869	3.08823	0.00046
C 13	3.06188	3.06029	0.00159
C 14	3.09663	3.08913	0.0075
C 15	3.10341	3.1044	-0.00099
O 16	4.37968	4.38389	-0.00421
N 17	3.61652	3.45559	0.16093
N 18	3.90819	3.21783	0.69036
H 19	0.39106	0.39096	0.0001
H 20	0.389	0.38888	0.00012
H 21	0.36963	0.36993	-0.0003

H 22	0.38668	0.38662	6E-05
H 23	0.26947	0.26803	0.00144
H 24	0.37064	0.37066	-2E-05
H 25	0.37167	0.3717	-3E-05
H 26	0.37228	0.37247	-0.00019
H 27	0.37067	0.37055	0.00012
C 28	2.7741	2.77165	0.00245
C 29	2.77622	2.77584	0.00038
C 30	2.77593	2.77615	-0.00022
N 31	3.75777	3.75662	0.00115
N 32	3.75668	3.75627	0.00041
N 33	3.76678	3.76435	0.00243
C 34	3.04396	3.04391	5E-05
C 35	3.09769	3.09775	-6E-05
C 36	3.10031	3.10025	6E-05
C 37	3.12285	3.12277	8E-05
H 38	0.36736	0.36736	0
C 39	3.12428	3.12426	2E-05
H 40	0.37041	0.37041	0
C 41	3.11475	3.11474	1E-05
H 42	0.37864	0.37864	0
H 43	0.38037	0.38037	0
H 44	0.38071	0.38071	0
C 45	3.04422	3.04426	-4E-05
C 46	3.09744	3.09738	6E-05
C 47	3.10053	3.10028	0.00025
C 48	3.12256	3.12256	0
H 49	0.36714	0.36714	0
C 50	3.12432	3.12439	-7E-05
H 51	0.37059	0.3706	-1E-05
C 52	3.11467	3.11448	0.00019
H 53	0.37828	0.37828	0
H 54	0.38044	0.38044	0
H 55	0.38064	0.38065	-1E-05

Table S30. BBO–COF 2 formation with NaCN: Before H atom abstraction

Energy: –1418.09462218 hartrees

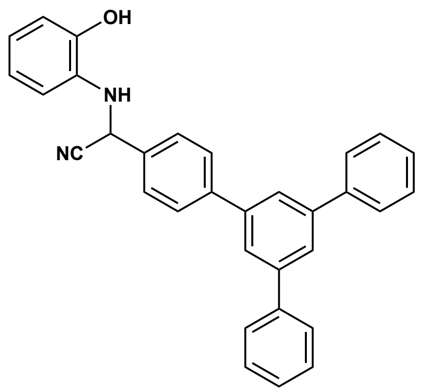

C	8.69112	0.88247	-1.25297
C	8.44435	-0.46505	-1.54552
C	7.18710	-0.99873	-1.21744
C	6.18699	-0.21375	-0.64631
C	6.40435	1.18739	-0.34075
C	7.70951	1.67744	-0.65883
O	5.47559	1.91725	0.17916
N	4.88204	-0.74790	-0.43028
C	4.26084	-0.51700	0.88221
C	4.70668	-1.48970	1.90259
N	5.06692	-2.29682	2.66147
C	2.74312	-0.45625	0.75905
C	2.20792	0.57783	-0.02697
C	0.83188	0.69683	-0.19703
C	-0.05973	-0.21415	0.40410
C	0.48413	-1.24203	1.18943
C	1.86652	-1.35872	1.36766
C	-1.52884	-0.08805	0.21590
C	-2.14756	1.17261	0.18946
C	-3.53280	1.30943	0.01384
C	-4.31109	0.15129	-0.13604
C	-3.72564	-1.12372	-0.11568
C	-2.33737	-1.22569	0.05865
H	9.66229	1.31878	-1.49013
H	9.20669	-1.09295	-2.00184
H	6.97840	-2.05129	-1.42293
H	7.90189	2.72640	-0.43868
H	4.81276	-1.72165	-0.71463
H	4.61688	0.49589	1.15771
H	2.89657	1.27560	-0.49641
H	0.44244	1.49456	-0.82550
H	-0.17646	-1.94502	1.69201
H	2.25859	-2.15509	1.99544
H	-1.53468	2.06160	0.30561
H	-5.38525	0.24332	-0.27089
H	-1.87339	-2.20762	0.07494
C	-4.16624	2.65606	-0.00977
C	-3.74076	3.67119	0.86597
C	-5.20769	2.95188	-0.90780
C	-4.33413	4.93482	0.84493
H	-2.95076	3.46151	1.58211
C	-5.80356	4.21448	-0.92865
H	-5.53534	2.19254	-1.61318
C	-5.36965	5.21303	-0.05190

H	-3.98953	5.70101	1.53538
H	-6.60048	4.42113	-1.63942
H	-5.83087	6.19750	-0.06946
C	-4.56153	-2.34388	-0.28111
C	-5.81864	-2.45215	0.34061
C	-4.11761	-3.42354	-1.06580
C	-6.60388	-3.59597	0.18295
H	-6.17198	-1.64150	0.97273
C	-4.90082	-4.56861	-1.22307
H	-3.15921	-3.35486	-1.57360
C	-6.14891	-4.66081	-0.60013
H	-7.56833	-3.65875	0.68186
H	-4.53724	-5.38699	-1.84010
H	-6.75876	-5.55268	-0.72208

Table S31. Vibrational frequencies for BBO–COF 2 formation with NaCN: Before H atom abstraction

12	19	22	40	43	46
47	54	60	76	94	118
119	124	137	176	193	219
236	255	267	289	294	311
327	348	373	375	402	418
419	421	453	471	479	484
507	528	557	563	582	594
610	623	626	628	636	639
649	655	681	691	709	709
710	725	735	747	771	774
776	824	831	836	848	857
857	858	869	877	895	912
920	921	927	934	936	954
971	979	979	996	996	1001
1009	1015	1016	1027	1042	1047
1054	1066	1074	1107	1108	1120
1120	1126	1148	1168	1189	1189
1210	1214	1214	1221	1227	1259
1267	1268	1285	1297	1324	1326
1331	1341	1345	1352	1366	1372
1372	1377	1403	1438	1451	1470
1477	1493	1499	1515	1533	1539

1540	1553	1578	1609	1623	1624
1626	1637	1638	1652	1653	1658
2303	2894	3113	3131	3165	3173
3173	3176	3176	3180	3181	3182
3190	3192	3194	3196	3198	3200
3202	3204	3206	3206	3207	3520

Table S32. BBO–COF 2 formation with NaCN: Radical intermediate

Energy: –1417.49659486 hartrees
$\langle S^2 \rangle = 0.7598$

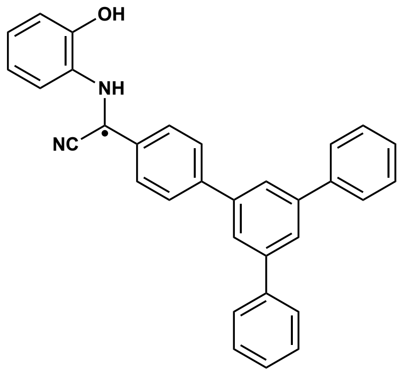

C	-9.09280	0.77514	0.31321
C	-8.66572	-0.23402	-0.57491
C	-7.31456	-0.57393	-0.64601
C	-6.38503	0.08834	0.17165
C	-6.79171	1.14711	1.10472
C	-8.19024	1.44909	1.12601
O	-5.90807	1.72769	1.81863
N	-5.02032	-0.10442	0.21001
C	-4.15088	-0.97753	-0.40203
C	-4.62708	-2.12216	-1.07470
N	-4.95042	-3.09195	-1.65165
C	-2.72490	-0.75349	-0.33167
C	-2.16221	0.42237	0.23744
C	-0.78787	0.60856	0.30447
C	0.12487	-0.34828	-0.18457
C	-0.43260	-1.51413	-0.75716
C	-1.80157	-1.71233	-0.83214
C	1.58553	-0.14253	-0.10684
C	2.14843	1.14704	-0.17701
C	3.53201	1.35763	-0.10233
C	4.38151	0.24859	0.03954
C	3.85814	-1.05228	0.10981
C	2.46963	-1.22972	0.03895
H	-10.15104	1.03147	0.36076
H	-9.38398	-0.74926	-1.20825
H	-6.98985	-1.34934	-1.33126
H	-8.51949	2.22945	1.80857
H	-4.63757	0.56254	0.89232
H	-2.80172	1.20874	0.62724
H	-0.41545	1.51571	0.77487
H	0.22311	-2.27079	-1.18204
H	-2.18045	-2.62044	-1.29229

H	1.49082	2.00322	-0.29289
H	5.45590	0.39895	0.09576
H	2.06311	-2.23473	0.09949
C	4.76409	-2.22329	0.26321
C	5.98282	-2.29405	-0.43596
C	4.42860	-3.29345	1.11248
C	6.83433	-3.39131	-0.29154
H	6.25254	-1.49137	-1.11748
C	5.27771	-4.39247	1.25645
H	3.50260	-3.25274	1.67981
C	6.48618	-4.44733	0.55573
H	7.76677	-3.42531	-0.85069
H	4.99673	-5.20395	1.92383
H	7.14754	-5.30321	0.66734
C	4.09368	2.73398	-0.17851
C	3.56462	3.68501	-1.06993
C	5.17032	3.12416	0.63864
C	4.09025	4.97656	-1.14094
H	2.74608	3.40207	-1.72639
C	5.69884	4.41466	0.56737
H	5.57964	2.41534	1.35387
C	5.16125	5.34861	-0.32310
H	3.66529	5.69111	-1.84211
H	6.52521	4.69367	1.21736
H	5.57020	6.35468	-0.37740

Table S33. Vibrational frequencies for BBO–COF 2 formation with NaCN: Radical intermediate

10	14	27	29	43	46
53	58	60	77	100	120
121	138	153	162	187	217
236	253	268	285	291	308
311	351	375	384	413	418
419	423	457	473	493	499
512	534	548	553	566	581
592	622	623	627	636	639
652	654	696	709	710	721
724	733	736	766	773	775
788	811	825	831	832	838
840	857	858	882	883	905
906	914	932	932	940	959

969	979	979	995	996	1006
1012	1015	1016	1024	1038	1048
1054	1066	1107	1107	1119	1119
1128	1163	1170	1188	1188	1213
1213	1219	1229	1266	1270	1274
1299	1313	1325	1330	1330	1345
1350	1365	1368	1372	1377	1388
1409	1447	1451	1476	1487	1492
1498	1518	1537	1539	1542	1556
1568	1593	1616	1622	1625	1630
1630	1648	1652	1653	2228	3143
3170	3170	3174	3175	3177	3179
3179	3184	3189	3191	3193	3197
3199	3200	3201	3202	3203	3205

Table S34. NPA Charge for BBO–COF 2 formation with NaCN: Radical intermediate

Cyano	Alpha Charge	Beta Charge	Charge Sum
C 1	-0.15928	-0.094	-0.25328
C 2	-0.16676	-0.13832	-0.30508
C 3	-0.17104	-0.10529	-0.27633
C 4	0.04167	0.02389	0.06556
C 5	0.1614	0.20962	0.37102
C 6	-0.18438	-0.15291	-0.33729
O 7	-0.41047	-0.32978	-0.74025
N 8	-0.37942	-0.12707	-0.50649
C 9	-0.09978	0.08408	-0.0157
C 10	0.13899	0.11119	0.25018
N 11	-0.26101	-0.1367	-0.39771
C 12	-0.02288	-0.04541	-0.06829
C 13	-0.16574	-0.08559	-0.25133
C 14	-0.08948	-0.1245	-0.21398
C 15	-0.0922	-0.00646	-0.09866
C 16	-0.10411	-0.1265	-0.23061
C 17	-0.13885	-0.08309	-0.22194
C 18	-0.01381	-0.02335	-0.03716
C 19	-0.11287	-0.09321	-0.20608

C 20	-0.02354	-0.03155	-0.05509
C 21	-0.12569	-0.09975	-0.22544
C 22	-0.02645	-0.03491	-0.06136
C 23	-0.1082	-0.08733	-0.19553
H 24	0.11117	0.10898	0.22015
H 25	0.1127	0.1117	0.2244
H 26	0.12323	0.12121	0.24444
H 27	0.11494	0.11398	0.22892
H 28	0.23002	0.22444	0.45446
H 29	0.12258	0.12048	0.24306
H 30	0.11618	0.11721	0.23339
H 31	0.11544	0.11612	0.23156
H 32	0.12554	0.12375	0.24929
H 33	0.12039	0.11975	0.24014
H 34	0.11783	0.11702	0.23485
H 35	0.12034	0.11967	0.24001
C 36	-0.02068	-0.01954	-0.04022
C 37	-0.11268	-0.11352	-0.2262
C 38	-0.11097	-0.11188	-0.22285
C 39	-0.1207	-0.12029	-0.24099
H 40	0.12113	0.12118	0.24231
C 41	-0.11953	-0.11909	-0.23862
H 42	0.12335	0.12339	0.24674
C 43	-0.12559	-0.1264	-0.25199
H 44	0.11881	0.1188	0.23761
H 45	0.12	0.11998	0.23998
H 46	0.11845	0.11848	0.23693
C 47	-0.02096	-0.01986	-0.04082
C 48	-0.11087	-0.11173	-0.2226
C 49	-0.11264	-0.11341	-0.22605
C 50	-0.11937	-0.11895	-0.23832
H 51	0.12323	0.12326	0.24649
C 52	-0.12059	-0.12021	-0.2408
H 53	0.12109	0.12113	0.24222
C 54	-0.12542	-0.12615	-0.25157
H 55	0.12009	0.12008	0.24017
H 56	0.11885	0.11883	0.23768
H 57	0.11852	0.11855	0.23707

Table S35. NPA Spin for BBO–COF 2 formation with NaCN: Radical intermediate

Cyano	Alpha Spin	Beta Spin	Alpha – Beta Difference Density
C 1	3.15928	3.094	0.06528
C 2	3.16676	3.13832	0.02844
C 3	3.17104	3.10529	0.06575
C 4	2.95833	2.97611	-0.01778
C 5	2.8386	2.79038	0.04822
C 6	3.18438	3.15291	0.03147
O 7	4.41047	4.32978	0.08069
N 8	3.87942	3.62707	0.25235
C 9	3.09978	2.91592	0.18386
C 10	2.86101	2.88881	-0.0278
N 11	3.76101	3.6367	0.12431
C 12	3.02288	3.04541	-0.02253
C 13	3.16574	3.08559	0.08015
C 14	3.08948	3.1245	-0.03502
C 15	3.0922	3.00646	0.08574
C 16	3.10411	3.1265	-0.02239
C 17	3.13885	3.08309	0.05576
C 18	3.01381	3.02335	-0.00954
C 19	3.11287	3.09321	0.01966
C 20	3.02354	3.03155	-0.00801
C 21	3.12569	3.09975	0.02594
C 22	3.02645	3.03491	-0.00846
C 23	3.1082	3.08733	0.02087
H 24	0.38883	0.39102	-0.00219
H 25	0.3873	0.3883	-0.001
H 26	0.37677	0.37879	-0.00202
H 27	0.38506	0.38602	-0.00096
H 28	0.26998	0.27556	-0.00558
H 29	0.37742	0.37952	-0.0021
H 30	0.38382	0.38279	0.00103
H 31	0.38456	0.38388	0.00068
H 32	0.37446	0.37625	-0.00179
H 33	0.37961	0.38025	-0.00064

H 34	0.38217	0.38298	-0.00081
H 35	0.37966	0.38033	-0.00067
C 36	3.02068	3.01954	0.00114
C 37	3.11268	3.11352	-0.00084
C 38	3.11097	3.11188	-0.00091
C 39	3.1207	3.12029	0.00041
H 40	0.37887	0.37882	5E-05
C 41	3.11953	3.11909	0.00044
H 42	0.37665	0.37661	4E-05
C 43	3.12559	3.1264	-0.00081
H 44	0.38119	0.3812	-1E-05
H 45	0.38	0.38002	-2E-05
H 46	0.38155	0.38152	3E-05
C 47	3.02096	3.01986	0.0011
C 48	3.11087	3.11173	-0.00086
C 49	3.11264	3.11341	-0.00077
C 50	3.11937	3.11895	0.00042
H 51	0.37677	0.37674	3E-05
C 52	3.12059	3.12021	0.00038
H 53	0.37891	0.37887	4E-05
C 54	3.12542	3.12615	-0.00073
H 55	0.37991	0.37992	-1E-05
H 56	0.38115	0.38117	-2E-05
H 57	0.38148	0.38145	3E-05

Table S36. BBO–COF 3 formation with NaCN: Before H atom abstraction

Energy: –1466.23927512 hartrees

C	-8.67945	1.21174	0.27424
C	-8.40692	0.40402	1.38579
C	-7.12506	-0.15729	1.50485
C	-6.12608	0.10159	0.56808
C	-6.37137	0.93973	-0.58822
C	-7.69886	1.45964	-0.68758
N	-4.80101	-0.38725	0.76774
C	-4.14143	-1.03776	-0.37561
C	-4.55215	-2.44666	-0.54838
N	-4.88812	-3.55910	-0.62850
O	-5.44295	1.18350	-1.45248
C	-2.63163	-0.87398	-0.29673

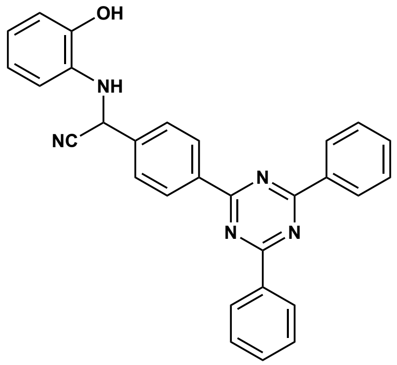

C	-2.13196	0.44111	-0.27810
C	-0.76484	0.67600	-0.21178
C	0.14883	-0.39717	-0.15685
C	-0.35335	-1.70918	-0.17895
C	-1.72680	-1.94215	-0.25094
C	1.59879	-0.14865	-0.08167
N	2.03009	1.12799	-0.05741
C	3.35323	1.30593	0.01026
N	4.25395	0.30965	0.05539
C	3.73830	-0.93055	0.02807
N	2.43256	-1.20823	-0.03967
H	-9.67014	1.65183	0.15470
H	-9.16835	0.19977	2.13544
H	-6.89619	-0.80280	2.35598
H	-7.91120	2.09114	-1.54875
H	-4.72255	-0.94522	1.61429
H	-4.50048	-0.43732	-1.24150
H	-2.84243	1.26133	-0.32695
H	-0.38602	1.69222	-0.20669
H	0.33986	-2.54228	-0.13640
H	-2.09639	-2.96397	-0.27141
C	4.68845	-2.07577	0.07604
C	4.21044	-3.39622	0.05404
C	6.07327	-1.85194	0.14319
C	5.10014	-4.46983	0.09888
H	3.14033	-3.56371	0.00147
C	6.96123	-2.92715	0.18774
H	6.43820	-0.83083	0.15911
C	6.47814	-4.23957	0.16583
H	4.71744	-5.48734	0.08130
H	8.03140	-2.74107	0.23925
H	7.17080	-5.07731	0.20048
C	3.86297	2.70363	0.03815
C	2.96813	3.78541	-0.00711
C	5.24201	2.96025	0.10974
C	3.44429	5.09634	0.01870
H	1.90426	3.58288	-0.06231
C	5.71572	4.27223	0.13536
H	5.92969	2.12234	0.14475
C	4.81890	5.34470	0.08982
H	2.74115	5.92472	-0.01711
H	6.78573	4.45827	0.19068
H	5.18869	6.36726	0.10946

Table S37. Vibrational frequencies for BBO–COF 3 formation with NaCN: Before H atom abstraction

12	20	23	29	33	40
48	51	68	72	100	107
109	126	164	172	193	215
224	232	258	277	281	313
327	348	375	418	419	420
424	446	453	459	479	491
504	526	558	564	596	607
612	631	632	645	660	668
680	689	699	701	710	712
714	737	751	772	792	813
832	838	857	858	862	866
867	868	881	885	917	929
956	956	958	988	996	998
1008	1009	1010	1011	1017	1019
1037	1045	1047	1051	1070	1077
1103	1104	1126	1144	1168	1174
1175	1188	1189	1204	1206	1209
1219	1222	1231	1259	1283	1332
1332	1333	1341	1348	1361	1363
1376	1397	1401	1409	1435	1456
1477	1484	1488	1514	1531	1535
1538	1544	1551	1569	1580	1612
1626	1633	1637	1653	1653	1655
2305	2829	3114	3132	3167	3175
3175	3177	3188	3188	3195	3200
3200	3211	3224	3227	3227	3229

Table S38. BBO–COF 3 formation with NaCN: Radical intermediate

Energy: –1465.64800558
$<S^2> = 0.7579$

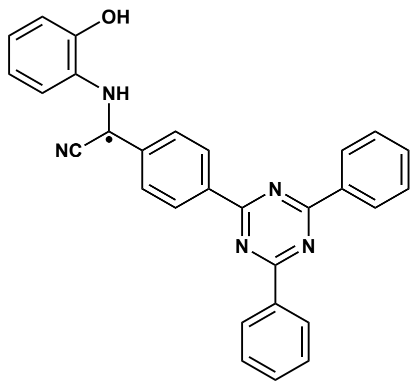

C	-9.02140	0.72005	-0.18565
C	-8.57316	-0.59222	-0.45878
C	-7.21984	-0.90388	-0.36160
C	-6.30079	0.09175	0.01356
C	-6.72976	1.46886	0.30118
C	-8.13524	1.72118	0.17878
N	-4.93616	-0.03341	0.13710
C	-4.04419	-1.07319	0.03119
C	-4.50861	-2.40882	-0.02710
N	-4.82929	-3.53456	-0.07817
O	-5.86203	2.34015	0.62772
C	-2.62901	-0.82961	0.01167
C	-2.07837	0.48741	-0.02472
C	-0.71295	0.69750	-0.02657
C	0.20578	-0.37911	0.00059
C	-0.33352	-1.69027	0.02567
C	-1.69541	-1.90914	0.03174
C	1.63828	-0.14960	-0.00391
N	2.09657	1.12855	-0.02754
C	3.41837	1.28404	-0.03207
N	4.31531	0.27814	-0.01453
C	3.77681	-0.95632	0.00924
N	2.47227	-1.22162	0.01514
H	-10.08394	0.94719	-0.26560
H	-9.28221	-1.36371	-0.74862
H	-6.88354	-1.91212	-0.57497
H	-8.47729	2.73226	0.38648
H	-4.56599	0.89149	0.39206
H	-2.72787	1.35659	-0.07165
H	-0.32788	1.71128	-0.05817
H	0.34731	-2.53474	0.04362
H	-2.06908	-2.92861	0.05645
C	4.71735	-2.11332	0.03046
C	6.10607	-1.90527	0.02516
C	4.22699	-3.42916	0.05610
C	6.98480	-2.98960	0.04504
H	6.48041	-0.88759	0.00547
C	5.10655	-4.51196	0.07602
H	3.15363	-3.58448	0.06014

C	6.48898	-4.29700	0.07054
H	8.05823	-2.81382	0.04068
H	4.71247	-5.52537	0.09585
H	7.17398	-5.14184	0.08609
C	3.95076	2.67629	-0.05797
C	3.07406	3.77357	-0.06994
C	5.33512	2.91182	-0.07035
C	3.57165	5.07670	-0.09395
H	2.00577	3.58690	-0.05975
C	5.83138	4.21619	-0.09468
H	6.00864	2.06182	-0.06066
C	4.95212	5.30367	-0.10654
H	2.88076	5.91643	-0.10255
H	6.90604	4.38410	-0.10420
H	5.33908	6.32009	-0.12522

Table S39. Vibrational Frequencies for BBO–COF 3 formation with NaCN: Radical intermediate

11	15	27	29	30	34
57	64	71	73	91	114
130	138	153	178	184	222
223	232	253	264	285	308
314	349	389	420	421	424
429	453	458	461	474	502
517	536	547	549	582	591
611	631	632	644	660	670
685	687	702	708	711	727
740	745	768	785	797	811
815	831	835	844	852	857
863	864	866	883	913	948
953	954	983	988	995	996
1000	1006	1007	1015	1016	1018
1024	1044	1047	1050	1076	1101
1102	1131	1155	1169	1172	1173
1186	1187	1201	1203	1220	1228
1245	1273	1316	1329	1333	1341
1345	1360	1362	1372	1383	1393
1410	1411	1445	1473	1481	1489
1492	1508	1519	1534	1537	1548
1553	1565	1569	1593	1629	1631

1635	1651	1652	1653	2247	3150
3170	3170	3182	3182	3183	3190
3192	3195	3195	3198	3220	3221
3226	3227	3228	3229	3229	3349

Table S40. NPA Charge for BBO–COF 3 formation with NaCN: Radical intermediate

Cyano	Alpha Charge	Beta Charge	Charge Sum
C 1	-0.15375	-0.09518	-0.24893
C 2	-0.1705	-0.12359	-0.29409
C 3	-0.16188	-0.11261	-0.27449
C 4	0.03303	0.03764	0.07067
C 5	0.16389	0.21152	0.37541
C 6	-0.18381	-0.14475	-0.32856
N 7	-0.36844	-0.12945	-0.49789
C 8	-0.05708	0.05632	-0.00076
C 9	0.13646	0.11836	0.25482
N 10	-0.23003	-0.14907	-0.3791
O 11	-0.41052	-0.31012	-0.72064
C 12	-0.03002	-0.02428	-0.0543
C 13	-0.15886	-0.10598	-0.26484
C 14	-0.07599	-0.0937	-0.16969
C 15	-0.10846	-0.04702	-0.15548
C 16	-0.09312	-0.08987	-0.18299
C 17	-0.14148	-0.10657	-0.24805
C 18	0.19885	0.23055	0.4294
N 19	-0.27049	-0.25158	-0.52207
C 20	0.22363	0.22121	0.44484
N 21	-0.29958	-0.25709	-0.55667
C 22	0.22187	0.21751	0.43938
N 23	-0.27247	-0.25131	-0.52378
H 24	0.11276	0.11077	0.22353
H 25	0.11454	0.11297	0.22751
H 26	0.12422	0.12265	0.24687
H 27	0.11683	0.11562	0.23245
H 28	0.23129	0.2255	0.45679
H 29	0.12127	0.11998	0.24125

H 30	0.12636	0.12686	0.25322
H 31	0.12578	0.12568	0.25146
H 32	0.1248	0.12367	0.24847
C 33	-0.04034	-0.03946	-0.0798
C 34	-0.10287	-0.10214	-0.20501
C 35	-0.09915	-0.10018	-0.19933
C 36	-0.1255	-0.12513	-0.25063
H 37	0.1297	0.12968	0.25938
C 38	-0.12478	-0.12378	-0.24856
H 39	0.1324	0.13241	0.26481
C 40	-0.11851	-0.11846	-0.23697
H 41	0.11826	0.11824	0.2365
H 42	0.11971	0.11968	0.23939
H 43	0.11777	0.11777	0.23554
C 44	-0.04784	-0.04726	-0.0951
C 45	-0.09667	-0.09731	-0.19398
C 46	-0.10098	-0.0997	-0.20068
C 47	-0.12444	-0.12367	-0.24811
H 48	0.13259	0.1326	0.26519
C 49	-0.12553	-0.12544	-0.25097
H 50	0.12942	0.12938	0.2588
C 51	-0.11859	-0.11799	-0.23658
H 52	0.12018	0.12016	0.24034
H 53	0.11817	0.11817	0.23634
H 54	0.11786	0.11784	0.2357

Table S41. NPA Spin for BBO–COF 3 formation with NaCN: Radical intermediate

Cyano	Alpha Spin	Beta Spin	Alpha – Beta Difference Density
C 1	3.15375	3.09518	0.05857
C 2	3.1705	3.12359	0.04691
C 3	3.16188	3.11261	0.04927
C 4	2.96697	2.96236	0.00461
C 5	2.83611	2.78848	0.04763
C 6	3.18381	3.14475	0.03906
N 7	3.86844	3.62945	0.23899
C 8	3.05708	2.94368	0.1134

C 9	2.86354	2.88164	-0.0181
N 10	3.73003	3.64907	0.08096
O 11	4.41052	4.31012	0.1004
C 12	3.03002	3.02428	0.00574
C 13	3.15886	3.10598	0.05288
C 14	3.07599	3.0937	-0.01771
C 15	3.10846	3.04702	0.06144
C 16	3.09312	3.08987	0.00325
C 17	3.14148	3.10657	0.03491
C 18	2.80115	2.76945	0.0317
N 19	3.77049	3.75158	0.01891
C 20	2.77637	2.77879	-0.00242
N 21	3.79958	3.75709	0.04249
C 22	2.77813	2.78249	-0.00436
N 23	3.77247	3.75131	0.02116
H 24	0.38724	0.38923	-0.00199
H 25	0.38546	0.38703	-0.00157
H 26	0.37578	0.37735	-0.00157
H 27	0.38317	0.38438	-0.00121
H 28	0.26871	0.2745	-0.00579
H 29	0.37873	0.38002	-0.00129
H 30	0.37364	0.37314	0.0005
H 31	0.37422	0.37432	-1E-04
H 32	0.3752	0.37633	-0.00113
C 33	3.04034	3.03946	0.00088
C 34	3.10287	3.10214	0.00073
C 35	3.09915	3.10018	-0.00103
C 36	3.1255	3.12513	0.00037
H 37	0.3703	0.37032	-2E-05
C 38	3.12478	3.12378	0.001
H 39	0.3676	0.36759	1E-05
C 40	3.11851	3.11846	5E-05
H 41	0.38174	0.38176	-2E-05
H 42	0.38029	0.38032	-3E-05
H 43	0.38223	0.38223	0
C 44	3.04784	3.04726	0.00058
C 45	3.09667	3.09731	-0.00064
C 46	3.10098	3.0997	0.00128
C 47	3.12444	3.12367	0.00077

H 48	0.36741	0.3674	1E-05
C 49	3.12553	3.12544	9E-05
H 50	0.37058	0.37062	-4E-05
C 51	3.11859	3.11799	0.0006
H 52	0.37982	0.37984	-2E-05
H 53	0.38183	0.38183	0
H 54	0.38214	0.38216	-2E-05

Table S42 BBO–COF 2 formation with $NaSCH_3$: Before H atom abstraction
Energy: –1763.35572631 hartrees

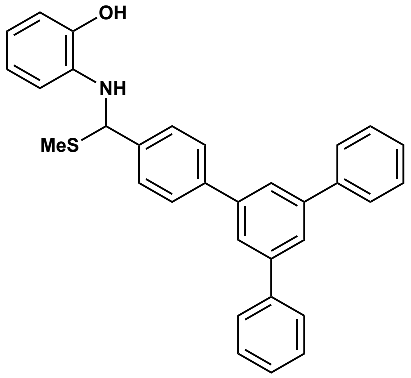

C	-8.21999	1.78463	1.18125
C	-7.07611	1.70621	1.98454
C	-5.89743	1.16803	1.43083
C	-5.87485	0.69664	0.12238
C	-7.04089	0.76483	-0.74051
C	-8.20599	1.33423	-0.14334
N	-4.71288	0.16245	-0.49847
C	-3.95636	-0.86866	0.14605
S	-4.23364	-2.60507	-0.59339
C	-2.44760	-0.66139	0.11642
C	-1.86570	0.32716	-0.68514
C	-0.48243	0.51058	-0.70642
C	0.37050	-0.28521	0.07724
C	-0.21910	-1.27268	0.88758
C	-1.60066	-1.45639	0.90456
C	1.84333	-0.08810	0.05499
C	2.39856	1.19931	-0.03007
C	3.78632	1.40267	-0.05800
C	4.63294	0.28544	0.00697
C	4.11241	-1.01467	0.09477
C	2.71998	-1.18395	0.11518
O	-6.97252	0.30864	-1.94293
C	-6.06355	-2.68744	-0.56179
H	-9.13951	2.20432	1.59216
H	-7.08465	2.06655	3.01140
H	-4.98669	1.13385	2.03065
H	-9.10390	1.38899	-0.75745
H	-4.96078	-0.03470	-1.47237
H	-4.28263	-0.93715	1.18971
H	-2.51865	0.95709	-1.28112

H	-0.05878	1.26923	-1.36076
H	0.40712	-1.88364	1.53385
H	-2.03145	-2.22531	1.54182
H	1.73358	2.05652	-0.07916
H	5.70967	0.42920	-0.01216
H	2.30705	-2.18638	0.17985
H	-6.49766	-1.85568	-1.12790
H	-6.33403	-3.63352	-1.04284
H	-6.44323	-2.68533	0.46474
C	5.02139	-2.19147	0.16078
C	6.19793	-2.15642	0.93088
C	4.72950	-3.37197	-0.54619
C	7.05235	-3.25916	0.99082
H	6.43152	-1.26469	1.50691
C	5.58191	-4.47610	-0.48585
H	3.83719	-3.41670	-1.16493
C	6.74869	-4.42540	0.28242
H	7.95139	-3.20998	1.60098
H	5.33642	-5.37486	-1.04669
H	7.41241	-5.28529	0.33012
C	4.35063	2.77651	-0.15396
C	5.47137	3.04441	-0.96057
C	3.77790	3.84618	0.55750
C	6.00181	4.33281	-1.05132
H	5.91450	2.23997	-1.54193
C	4.30587	5.13548	0.46619
H	2.92290	3.66157	1.20241
C	5.42145	5.38569	-0.33814
H	6.86309	4.51569	-1.68984
H	3.84699	5.94437	1.02978
H	5.83167	6.38998	-0.41067

Table S43. Vibrational frequencies for BBO–COF 2 formation with $NaSCH_3$: Before H atom abstraction

10	16	25	32	43	46
50	53	56	65	94	99
109	120	133	145	170	183
202	225	236	243	270	287
294	302	306	333	357	389
401	417	418	420	422	467
477	497	507	526	571	575

583	590	623	625	627	629
636	642	654	668	681	706
709	709	710	725	734	756
771	775	776	806	828	835
844	848	857	858	858	872
879	898	903	919	921	923
934	936	969	979	979	990
997	997	1006	1009	1014	1015
1016	1026	1040	1047	1054	1066
1097	1108	1108	1120	1120	1135
1152	1167	1189	1189	1201	1205
1214	1215	1217	1251	1265	1267
1268	1296	1315	1325	1327	1341
1344	1351	1366	1369	1372	1372
1395	1400	1432	1451	1468	1489
1489	1492	1499	1503	1526	1534
1539	1540	1550	1585	1607	1623
1624	1625	1637	1638	1652	1654
1657	3034	3067	3123	3124	3129
3130	3161	3172	3172	3173	3173
3178	3181	3182	3191	3192	3192
3195	3198	3200	3202	3203	3206

Table S44. BBO–COF 2 formation with $NaSCH_3$: Radical intermediate

Energy: –1762.73603979 hartrees
$\langle S^2 \rangle = 0.7628$

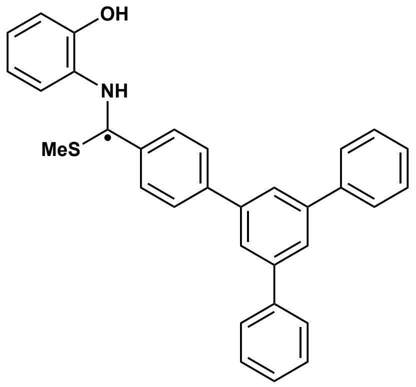

C	-6.22123	3.18602	0.76064
C	-5.03334	2.65416	1.29651
C	-4.60511	1.38305	0.89772
C	-5.35146	0.65045	-0.03169
C	-6.60480	1.15643	-0.59094
C	-6.98803	2.46333	-0.14838
N	-5.08029	-0.64403	-0.47248
C	-4.07723	-1.53770	-0.19682
S	-4.61249	-3.23463	-0.12308
C	-2.68823	-1.21427	-0.12850
C	-2.17503	-0.00279	-0.69178
C	-0.82506	0.29501	-0.66687
C	0.13377	-0.57524	-0.09224

C	-0.36890	-1.77915	0.46040
C	-1.71711	-2.08913	0.44844
C	1.56824	-0.25000	-0.08049
C	2.02129	1.08754	-0.07599
C	3.38400	1.41185	-0.05722
C	4.33283	0.37552	-0.03275
C	3.92199	-0.96817	-0.03453
C	2.55274	-1.26158	-0.06070
O	-7.27963	0.43175	-1.39823
C	-5.82387	-3.20737	1.26495
H	-6.54899	4.17979	1.06656
H	-4.44820	3.22083	2.01723
H	-3.69532	0.96261	1.31631
H	-7.91247	2.87138	-0.55219
H	-5.89055	-0.97309	-1.01388
H	-2.86118	0.68662	-1.17168
H	-0.49466	1.21366	-1.14666
H	0.31367	-2.47117	0.94896
H	-2.05328	-3.01660	0.90141
H	1.29125	1.88970	-0.02965
H	5.39174	0.61430	-0.07528
H	2.23969	-2.30106	-0.06822
H	-6.58993	-2.44788	1.09096
H	-6.29582	-4.19539	1.29186
H	-5.32122	-3.01498	2.21757
C	4.92894	-2.06409	-0.03419
C	6.09452	-1.97748	0.74916
C	4.74747	-3.21966	-0.81617
C	7.04136	-3.00377	0.75164
H	6.24626	-1.10545	1.38006
C	5.69165	-4.24830	-0.81338
H	3.86681	-3.30104	-1.44762
C	6.84499	-4.14626	-0.02977
H	7.92953	-2.91439	1.37344
H	5.52875	-5.12799	-1.43202
H	7.58081	-4.94696	-0.02765
C	3.82214	2.83414	-0.05821
C	3.15507	3.79874	-0.83532
C	4.91735	3.25767	0.71684
C	3.56486	5.13342	-0.83679
H	2.31887	3.49354	-1.45853
C	5.33092	4.59131	0.71490
H	5.43320	2.53681	1.34593
C	4.65629	5.53751	-0.06224

H	3.03313	5.85731	-1.45007
H	6.17530	4.89343	1.33073
H	4.97548	6.57705	-0.06254

Table S45. Vibrational frequencies for BBO–COF 2 formation with $NaSCH_3$: Radical intermediate

13	20	29	41	42	44
48	55	59	60	83	106
116	125	135	152	163	172
185	223	236	241	266	276
288	300	309	328	357	393
409	419	420	433	453	455
467	504	516	523	541	562
578	587	605	621	624	627
636	640	650	663	676	708
709	715	719	727	738	739
769	780	791	813	820	828
831	834	838	856	857	870
877	898	899	909	929	931
934	945	962	971	978	978
979	991	994	995	1004	1009
1014	1016	1031	1044	1054	1063
1106	1106	1111	1116	1118	1149
1164	1174	1188	1188	1212	1213
1217	1241	1264	1268	1274	1301
1317	1325	1331	1340	1347	1353
1364	1368	1371	1371	1388	1437
1444	1451	1476	1484	1488	1490
1497	1505	1511	1529	1535	1539
1542	1545	1586	1607	1618	1621
1626	1627	1642	1651	1653	3052
3132	3138	3151	3168	3169	3169
3171	3173	3177	3178	3178	3189
3190	3193	3196	3198	3199	3201
3202	3204	3206	3207	3220	3353

Table S46. NPA Charge for BBO–COF 2 formation with $NaSCH_3$: Radical intermediate

Sme	Alpha Charge	Beta Charge	Charge Sum
C 1	-0.15029	-0.11003	-0.26032
C 2	-0.17195	-0.14478	-0.31673
C 3	-0.16691	-0.11749	-0.2844
C 4	0.03515	0.01147	0.04662
C 5	0.16728	0.19601	0.36329
C 6	-0.18817	-0.15317	-0.34134
N 7	-0.37819	-0.19212	-0.57031
C 8	-0.14451	0.11136	-0.03315
S 9	0.0985	0.11141	0.20991
C 10	-0.04835	-0.07432	-0.12267
C 11	-0.17759	-0.06152	-0.23911
C 12	-0.08017	-0.13076	-0.21093
C 13	-0.12643	0.01827	-0.10816
C 14	-0.09805	-0.13587	-0.23392
C 15	-0.16384	-0.0684	-0.23224
C 16	-0.01028	-0.02457	-0.03485
C 17	-0.12487	-0.08526	-0.21013
C 18	-0.02059	-0.03625	-0.05684
C 19	-0.144	-0.09134	-0.23534
C 20	-0.02134	-0.03712	-0.05846
C 21	-0.12405	-0.08548	-0.20953
O 22	-0.40486	-0.35049	-0.75535
C 23	-0.42351	-0.40329	-0.8268
H 24	0.10961	0.10823	0.21784
H 25	0.1113	0.11045	0.22175
H 26	0.11927	0.11788	0.23715
H 27	0.11353	0.11253	0.22606
H 28	0.2295	0.22813	0.45763
H 29	0.12885	0.12542	0.25427
H 30	0.11409	0.1157	0.22979
H 31	0.11296	0.11411	0.22707
H 32	0.12232	0.11922	0.24154
H 33	0.12101	0.11976	0.24077
H 34	0.11789	0.11633	0.23422
H 35	0.11894	0.11771	0.23665
H 36	0.12926	0.12948	0.25874
H 37	0.11996	0.12119	0.24115

H 38	0.12013	0.12084	0.24097
C 39	-0.01971	-0.01757	-0.03728
C 40	-0.11235	-0.11379	-0.22614
C 41	-0.11071	-0.11238	-0.22309
C 42	-0.12134	-0.12061	-0.24195
H 43	0.12029	0.12039	0.24068
C 44	-0.12029	-0.11947	-0.23976
H 45	0.12325	0.12332	0.24657
C 46	-0.12636	-0.12773	-0.25409
H 47	0.11826	0.11824	0.2365
H 48	0.11938	0.11935	0.23873
H 49	0.11788	0.11794	0.23582
C 50	-0.02061	-0.01828	-0.03889
C 51	-0.10962	-0.11152	-0.22114
C 52	-0.11212	-0.11382	-0.22594
C 53	-0.11976	-0.11884	-0.2386
H 54	0.1231	0.1232	0.2463
C 55	-0.1215	-0.12065	-0.24215
H 56	0.11984	0.11998	0.23982
C 57	-0.12546	-0.12722	-0.25268
H 58	0.12001	0.11998	0.23999
H 59	0.11813	0.11812	0.23625
H 60	0.11804	0.11811	0.23615

Table S47. NPA Spin for BBO–COF 2 formation with $NaSCH_3$: Radical intermediate

Sme	Alpha Spin	Beta Spin	Alpha – Beta Difference Density
C 1	3.15029	3.11003	0.04026
C 2	3.17195	3.14478	0.02717
C 3	3.16691	3.11749	0.04942
C 4	2.96485	2.98853	-0.02368
C 5	2.83272	2.80399	0.02873
C 6	3.18817	3.15317	0.035
N 7	3.87819	3.69212	0.18607
C 8	3.14451	2.88864	0.25587
S 9	7.9015	7.88859	0.01291
C 10	3.04835	3.07432	-0.02597

C 11	3.17759	3.06152	0.11607
C 12	3.08017	3.13076	-0.05059
C 13	3.12643	2.98173	0.1447
C 14	3.09805	3.13587	-0.03782
C 15	3.16384	3.0684	0.09544
C 16	3.01028	3.02457	-0.01429
C 17	3.12487	3.08526	0.03961
C 18	3.02059	3.03625	-0.01566
C 19	3.144	3.09134	0.05266
C 20	3.02134	3.03712	-0.01578
C 21	3.12405	3.08548	0.03857
O 22	4.40486	4.35049	0.05437
C 23	3.42351	3.40329	0.02022
H 24	0.39039	0.39177	-0.00138
H 25	0.3887	0.38955	-0.00085
H 26	0.38073	0.38212	-0.00139
H 27	0.38647	0.38747	-0.001
H 28	0.2705	0.27187	-0.00137
H 29	0.37115	0.37458	-0.00343
H 30	0.38591	0.3843	0.00161
H 31	0.38704	0.38589	0.00115
H 32	0.37768	0.38078	-0.0031
H 33	0.37899	0.38024	-0.00125
H 34	0.38211	0.38367	-0.00156
H 35	0.38106	0.38229	-0.00123
H 36	0.37074	0.37052	0.00022
H 37	0.38004	0.37881	0.00123
H 38	0.37987	0.37916	0.00071
C 39	3.01971	3.01757	0.00214
C 40	3.11235	3.11379	-0.00144
C 41	3.11071	3.11238	-0.00167
C 42	3.12134	3.12061	0.00073
H 43	0.37971	0.37961	1E-04
C 44	3.12029	3.11947	0.00082
H 45	0.37675	0.37668	7E-05
C 46	3.12636	3.12773	-0.00137
H 47	0.38174	0.38176	-2E-05
H 48	0.38062	0.38065	-3E-05
H 49	0.38212	0.38206	6E-05

C 50	3.02061	3.01828	0.00233
C 51	3.10962	3.11152	-0.0019
C 52	3.11212	3.11382	-0.0017
C 53	3.11976	3.11884	0.00092
H 54	0.3769	0.3768	1E-04
C 55	3.1215	3.12065	0.00085
H 56	0.38016	0.38002	0.00014
C 57	3.12546	3.12722	-0.00176
H 58	0.37999	0.38002	-3E-05
H 59	0.38187	0.38188	-1E-05
H 60	0.38196	0.38189	7E-05

Table S48. BBO–COF 3 formation with $NaSCH_3$: Before H atom abstraction

Energy: –1811.49936069 hartrees

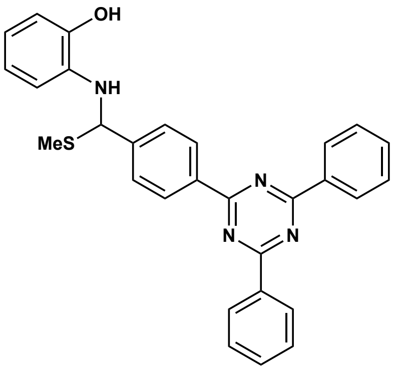

C	8.75945	0.21375	-1.20461
C	8.06515	-0.96298	-1.51664
C	6.68399	-1.02790	-1.25666
C	6.02098	0.05718	-0.69653
C	6.70942	1.28902	-0.34726
C	8.10619	1.31135	-0.63715
N	4.63369	0.12296	-0.43964
O	6.04476	2.25155	0.19167
C	3.87616	-0.96045	0.04090
S	4.33913	-1.58050	1.85076
C	4.34875	0.02939	2.71523
C	2.38539	-0.71194	0.00709
C	1.82959	0.57821	0.01387
C	0.44912	0.76431	0.00241
C	-0.42801	-0.33431	-0.03283
C	0.12609	-1.62981	-0.04980
C	1.50243	-1.80783	-0.02423
C	-1.88540	-0.13688	-0.05844
N	-2.68148	-1.22487	-0.09910
C	-3.99785	-0.99407	-0.12142
N	-4.55843	0.22789	-0.10599
C	-3.69257	1.25476	-0.06528
N	-2.36280	1.12494	-0.04042
H	9.83028	0.27432	-1.40299
H	8.58020	-1.81777	-1.95010
H	6.13542	-1.93856	-1.49899
H	8.65475	2.21816	-0.38765

H	4.48366	1.03361	0.00824
H	4.10677	-1.86706	-0.52444
H	3.33917	0.44510	2.81208
H	4.75192	-0.15207	3.71646
H	4.99974	0.74337	2.19916
H	2.47518	1.45023	0.00967
H	0.03816	1.76795	0.00991
H	-0.53717	-2.48741	-0.08263
H	1.91163	-2.81540	-0.01826
C	-4.25382	2.63327	-0.04680
C	-5.64293	2.83939	-0.06502
C	-3.39875	3.74698	-0.01112
C	-6.16544	4.13293	-0.04756
H	-6.29993	1.97692	-0.09265
C	-3.92347	5.03946	0.00596
H	-2.32683	3.58357	0.00250
C	-5.30793	5.23738	-0.01209
H	-7.24292	4.27949	-0.06169
H	-3.25049	5.89289	0.03323
H	-5.71561	6.24555	0.00136
C	-4.90721	-2.17104	-0.16678
C	-6.30065	-1.99593	-0.19577
C	-4.38312	-3.47435	-0.18108
C	-7.15099	-3.10116	-0.23853
H	-6.70139	-0.98830	-0.18425
C	-5.23530	-4.57785	-0.22372
H	-3.30679	-3.60473	-0.15819
C	-6.62200	-4.39591	-0.25271
H	-8.22802	-2.95227	-0.26072
H	-4.81674	-5.58130	-0.23424
H	-7.28550	-5.25700	-0.28599

Table S49. Vibrational frequencies for BBO–COF 3 formation with $NaSCH_3$: Before H atom abstraction

11	17	19	26	31	31
34	42	57	67	74	78
107	113	140	153	166	169
188	216	220	230	248	264
279	302	312	327	360	413
418	419	420	439	448	459
459	486	507	520	567	574
591	601	610	631	632	647

658	660	686	689	693	708
709	712	713	737	753	787
793	811	813	829	846	855
857	857	865	867	872	878
880	923	924	955	957	983
985	990	996	997	998	1007
1009	1010	1016	1018	1032	1047
1047	1050	1077	1103	1103	1104
1142	1158	1170	1174	1177	1188
1188	1203	1205	1209	1216	1228
1234	1265	1273	1318	1331	1333
1345	1358	1361	1363	1368	1386
1390	1395	1405	1436	1451	1479
1485	1496	1499	1507	1519	1524
1527	1536	1539	1551	1562	1594
1610	1630	1632	1636	1651	1652
1653	3037	3096	3116	3130	3132
3138	3166	3174	3175	3176	3183
3187	3188	3199	3200	3213	3223
3227	3229	3229	3231	3232	3380

Table S50. BBO–COF 3 formation with $NaSCH_3$: Radical intermediate

Energy: –1810.89202681 hartrees
$\langle S^2 \rangle = 0.7586$

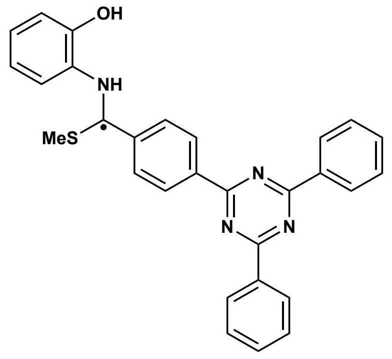

C	8.54958	1.43738	-0.40757
C	8.12127	0.31759	-1.14892
C	6.82579	-0.17438	-0.97336
C	5.96182	0.43873	-0.05550
C	6.36533	1.61368	0.71905
C	7.70336	2.07236	0.49225
N	4.63204	0.11411	0.19865
O	5.53318	2.15725	1.51839
C	3.81478	-0.93463	-0.09432
S	4.49711	-2.53776	-0.45248
C	5.63459	-2.79558	0.96748
C	2.39971	-0.74870	-0.09595
C	1.82723	0.56629	-0.14633
C	0.46372	0.76530	-0.12479
C	-0.44859	-0.32211	-0.06277
C	0.10883	-1.62986	-0.02761

C	1.47039	-1.83739	-0.04483
C	-1.87685	-0.10948	-0.04563
N	-2.70375	-1.19237	-0.00783
C	-4.00805	-0.93973	0.00637
N	-4.56431	0.28952	-0.01448
C	-3.67509	1.30512	-0.05140
N	-2.35407	1.16640	-0.06783
H	9.56273	1.81436	-0.54653
H	8.78846	-0.16013	-1.86252
H	6.48390	-1.02424	-1.55553
H	8.03213	2.94169	1.05728
H	4.25831	0.85312	0.81544
H	5.08413	-2.74725	1.91091
H	6.05682	-3.79749	0.83996
H	6.44119	-2.05846	0.96365
H	2.47995	1.42971	-0.23564
H	0.06722	1.77410	-0.17297
H	-0.56455	-2.47919	0.02332
H	1.85435	-2.85122	-0.00229
C	-4.22431	2.69161	-0.07519
C	-3.36178	3.80009	-0.09783
C	-5.61141	2.91115	-0.07451
C	-3.87517	5.09706	-0.11930
H	-2.29130	3.62558	-0.09706
C	-6.12390	4.20963	-0.09682
H	-6.27382	2.05258	-0.05632
C	-5.25859	5.30793	-0.11920
H	-3.19439	5.94508	-0.13555
H	-7.20076	4.36426	-0.09632
H	-5.65798	6.31968	-0.13591
C	-4.93678	-2.10680	0.04841
C	-4.43440	-3.41836	0.06757
C	-6.32746	-1.91297	0.06938
C	-5.30280	-4.50958	0.10666
H	-3.35946	-3.56232	0.05118
C	-7.19554	-3.00579	0.10871
H	-6.71098	-0.89862	0.05467
C	-6.68755	-4.30849	0.12746
H	-4.89860	-5.51931	0.12100
H	-8.27058	-2.83989	0.12482
H	-7.36406	-5.15989	0.15809

Table S51. Vibrational frequencies for BBO–COF 3 formation with $NaSCH_3$: Radical intermediate

14	19	28	29	31	37
53	64	70	72	93	99
117	134	143	146	167	176
202	223	228	231	249	263
268	287	326	340	364	422
422	434	439	442	458	461
463	495	513	532	550	560
589	610	631	632	637	654
660	667	680	685	687	694
709	711	724	740	745	782
799	810	821	834	836	844
848	856	859	864	865	878
913	943	950	950	951	986
988	988	994	996	996	1001
1004	1006	1009	1016	1018	1042
1046	1050	1072	1100	1100	1114
1149	1163	1168	1170	1185	1186
1196	1199	1201	1219	1251	1269
1293	1320	1326	1332	1339	1349
1359	1362	1374	1379	1387	1411
1414	1445	1446	1480	1484	1488
1491	1496	1508	1517	1533	1533
1540	1542	1545	1572	1591	1627
1630	1634	1648	1650	1651	3058
3143	3144	3154	3167	3167	3176
3179	3180	3185	3193	3193	3195
3203	3206	3219	3221	3226	3226

Table S52. NPA Charge for BBO–COF 3 formation with $NaSCH_3$: Radical intermediate

Sme	Alpha Charge	Beta Charge	Charge Sum
C 1	-0.14561	-0.10605	-0.25166
C 2	-0.1692	-0.1464	-0.3156
C 3	-0.16253	-0.1188	-0.28133

C 4	0.02826	0.01252	0.04078
C 5	0.16972	0.19869	0.36841
C 6	-0.18032	-0.1541	-0.33442
N 7	-0.36115	-0.18837	-0.54952
O 8	-0.39818	-0.34232	-0.7405
C 9	-0.06865	0.07569	0.00704
S 10	0.10906	0.12187	0.23093
C 11	-0.42005	-0.40538	-0.82543
C 12	-0.07343	-0.0453	-0.11873
C 13	-0.15695	-0.09309	-0.25004
C 14	-0.08895	-0.0953	-0.18425
C 15	-0.11919	-0.02473	-0.14392
C 16	-0.10172	-0.09475	-0.19647
C 17	-0.14609	-0.09439	-0.24048
C 18	0.16875	0.23908	0.40783
N 19	-0.28059	-0.24578	-0.52637
C 20	0.21921	0.21421	0.43342
N 21	-0.32641	-0.24316	-0.56957
C 22	0.22151	0.21808	0.43959
N 23	-0.2786	-0.24501	-0.52361
H 24	0.11122	0.10991	0.22113
H 25	0.11208	0.11133	0.22341
H 26	0.12015	0.11882	0.23897
H 27	0.11555	0.11477	0.23032
H 28	0.23282	0.22925	0.46207
H 29	0.1241	0.12488	0.24898
H 30	0.12261	0.1235	0.24611
H 31	0.13166	0.13104	0.2627
H 32	0.12315	0.12138	0.24453
H 33	0.12617	0.12637	0.25254
H 34	0.12478	0.12454	0.24932
H 35	0.12256	0.12088	0.24344
C 36	-0.04631	-0.04531	-0.09162
C 37	-0.0972	-0.09828	-0.19548
C 38	-0.10331	-0.10009	-0.2034
C 39	-0.12563	-0.12405	-0.24968
H 40	0.13251	0.13251	0.26502
C 41	-0.12598	-0.12606	-0.25204
H 42	0.12943	0.12934	0.25877

C 43	-0.12108	-0.11938	-0.24046
H 44	0.11944	0.11939	0.23883
H 45	0.11742	0.11741	0.23483
H 46	0.1171	0.11704	0.23414
C 47	-0.03848	-0.0372	-0.07568
C 48	-0.10004	-0.10149	-0.20153
C 49	-0.10524	-0.10236	-0.2076
C 50	-0.12624	-0.12444	-0.25068
H 51	0.13193	0.13194	0.26387
C 52	-0.12579	-0.12568	-0.25147
H 53	0.12992	0.12984	0.25976
C 54	-0.12132	-0.12001	-0.24133
H 55	0.11858	0.11853	0.23711
H 56	0.11758	0.11757	0.23515
H 57	0.11696	0.11691	0.23387

Table S53. NPA Spin for BBO–COF 3 formation with $NaSCH_3$: Radical intermediate

Sme	Alpha Spin	Beta Spin	Alpha – Beta Difference Density
C 1	3.14561	3.10605	0.03956
C 2	3.1692	3.1464	0.0228
C 3	3.16253	3.1188	0.04373
C 4	2.97174	2.98748	-0.01574
C 5	2.83028	2.80131	0.02897
C 6	3.18032	3.1541	0.02622
N 7	3.86115	3.68837	0.17278
O 8	4.39818	4.34232	0.05586
C 9	3.06865	2.92431	0.14434
S 10	7.89094	7.87813	0.01281
C 11	3.42005	3.40538	0.01467
C 12	3.07343	3.0453	0.02813
C 13	3.15695	3.09309	0.06386
C 14	3.08895	3.0953	-0.00635
C 15	3.11919	3.02473	0.09446
C 16	3.10172	3.09475	0.00697
C 17	3.14609	3.09439	0.0517
C 18	2.83125	2.76092	0.07033

N	19	3.78059	3.74578	0.03481
C	20	2.78079	2.78579	-0.005
N	21	3.82641	3.74316	0.08325
C	22	2.77849	2.78192	-0.00343
N	23	3.7786	3.74501	0.03359
H	24	0.38878	0.39009	-0.00131
H	25	0.38792	0.38867	-0.00075
H	26	0.37985	0.38118	-0.00133
H	27	0.38445	0.38523	-0.00078
H	28	0.26718	0.27075	-0.00357
H	29	0.3759	0.37512	0.00078
H	30	0.37739	0.3765	0.00089
H	31	0.36834	0.36896	-0.00062
H	32	0.37685	0.37862	-0.00177
H	33	0.37383	0.37363	0.0002
H	34	0.37522	0.37546	-0.00024
H	35	0.37744	0.37912	-0.00168
C	36	3.04631	3.04531	0.001
C	37	3.0972	3.09828	-0.00108
C	38	3.10331	3.10009	0.00322
C	39	3.12563	3.12405	0.00158
H	40	0.36749	0.36749	0
C	41	3.12598	3.12606	-8E-05
H	42	0.37057	0.37066	-9E-05
C	43	3.12108	3.11938	0.0017
H	44	0.38056	0.38061	-5E-05
H	45	0.38258	0.38259	-1E-05
H	46	0.3829	0.38296	-6E-05
C	47	3.03848	3.0372	0.00128
C	48	3.10004	3.10149	-0.00145
C	49	3.10524	3.10236	0.00288
C	50	3.12624	3.12444	0.0018
H	51	0.36807	0.36806	1E-05
C	52	3.12579	3.12568	0.00011
H	53	0.37008	0.37016	-8E-05
C	54	3.12132	3.12001	0.00131
H	55	0.38142	0.38147	-5E-05
H	56	0.38242	0.38243	-1E-05
H	57	0.38304	0.38309	-5E-05

Computational data for Benzobisoxazoles computed at the B3LYP/6-31G* level of theory

Table S54. BBO–COF 2 planarity model calculations

Energy: –2415.67715495 hartrees

C	0.08120	-1.18671	-0.86631
C	1.18374	-0.51473	-0.36807
C	1.13912	0.62837	0.46653
C	-0.08120	1.18663	0.86646
C	-1.18374	0.51466	0.36821
C	-1.13912	-0.62843	-0.46639
N	2.43956	1.01615	0.75794
C	3.19313	0.15674	0.13516
O	2.50849	-0.80941	-0.57449
C	4.64802	0.10582	0.10643
C	5.33356	-0.88558	-0.61315
C	6.72356	-0.90839	-0.62360
C	7.47385	0.05021	0.07937
C	6.77398	1.03828	0.79705
C	5.38643	1.06978	0.81328
C	8.95800	0.02357	0.06642
C	9.69423	1.21621	0.01307
C	11.09593	1.20906	-0.01833
C	11.75943	-0.02623	0.00747
C	11.05365	-1.23686	0.06144
C	9.65283	-1.19432	0.08912
C	11.86080	2.48208	-0.07751
C	11.77504	-2.53592	0.10659
C	11.43641	3.61393	0.63867
C	12.15198	4.80893	0.58093
C	13.30989	4.89818	-0.19371
C	13.74498	3.78164	-0.90991
C	13.02800	2.58749	-0.85252
C	11.31749	-3.58934	0.91590
C	11.99364	-4.80794	0.95542
C	13.14329	-4.99974	0.18705
C	13.61046	-3.96178	-0.62122
C	12.93344	-2.74375	-0.66073
N	-2.43956	-1.01623	-0.75778
C	-3.19313	-0.15673	-0.13512
O	-2.50849	0.80933	0.57464

C	-4.64803	-0.10582	-0.10639
C	-5.33356	0.88562	0.61314
C	-6.72356	0.90844	0.62358
C	-7.47385	-0.05020	-0.07934
C	-6.77398	-1.03832	-0.79695
C	-5.38643	-1.06982	-0.81317
C	-8.95800	-0.02356	-0.06640
C	-9.69424	-1.21619	-0.01302
C	-11.09593	-1.20904	0.01834
C	-11.75943	0.02625	-0.00751
C	-11.05365	1.23688	-0.06151
C	-9.65283	1.19433	-0.08917
C	-11.77503	2.53593	-0.10672
C	-11.86080	-2.48206	0.07755
C	-11.31747	3.58933	-0.91606
C	-11.99361	4.80792	-0.95565
C	-13.14327	4.99976	-0.18731
C	-13.61046	3.96183	0.62099
C	-12.93345	2.74380	0.66056
C	-11.43640	-3.61393	-0.63859
C	-12.15197	-4.80892	-0.58084
C	-13.30991	-4.89816	0.19378
C	-13.74501	-3.78159	0.90992
C	-13.02803	-2.58744	0.85252
H	0.14551	-2.06023	-1.50394
H	-0.14551	2.06014	1.50410
H	4.77340	-1.62904	-1.17020
H	7.23752	-1.66551	-1.20830
H	7.32929	1.77532	1.36959
H	4.85449	1.82977	1.37595
H	9.16760	2.16289	-0.05930
H	12.84461	-0.04432	0.03741
H	9.09512	-2.12491	0.13153
H	10.55144	3.54767	1.26548
H	11.80862	5.66919	1.14953
H	13.86812	5.82935	-0.23890
H	14.64058	3.84215	-1.52273
H	13.36073	1.73281	-1.43502
H	10.43959	-3.44163	1.53873
H	11.62613	-5.60595	1.59527
H	13.67056	-5.94935	0.21822
H	14.49993	-4.10259	-1.22975
H	13.29059	-1.95110	-1.31242
H	-4.77340	1.62912	1.17014

H	-7.23752	1.66559	1.20823
H	-7.32929	-1.77539	-1.36944
H	-4.85449	-1.82985	-1.37580
H	-9.16761	-2.16288	0.05938
H	-12.84461	0.04434	-0.03747
H	-9.09511	2.12492	-0.13160
H	-10.43955	3.44159	-1.53888
H	-11.62609	5.60591	-1.59552
H	-13.67054	5.94937	-0.21852
H	-14.49994	4.10266	1.22950
H	-13.29061	1.95117	1.31228
H	-10.55140	-3.54768	-1.26538
H	-11.80859	-5.66920	-1.14940
H	-13.86814	-5.82932	0.23897
H	-14.64063	-3.84208	1.52272
H	-13.36077	-1.73275	1.43498

Table S55. Vibrational frequencies for BBO–COF 2 planarity model calculations

6	7	12	17	26	27
35	44	44	46	47	48
50	57	58	59	67	79
88	104	113	118	123	135
154	164	177	192	211	233
238	240	248	266	272	281
287	288	307	308	322	350
362	370	376	396	397	407
415	417	418	419	420	420
421	425	444	450	455	509
510	528	531	535	541	550
585	585	624	624	626	626
628	628	636	636	642	642
654	654	656	658	709	709
713	714	715	715	715	717
719	726	726	736	746	760
760	776	776	785	785	807
823	839	839	842	845	859
859	861	861	862	862	862
865	867	868	887	905	905
919	919	926	926	936	937
937	937	938	946	970	970

971	971	976	976	989	989
997	997	997	997	1012	1012
1017	1017	1020	1020	1029	1029
1039	1040	1059	1059	1069	1070
1083	1090	1113	1113	1114	1114
1124	1124	1125	1125	1148	1149
1156	1171	1194	1194	1194	1194
1217	1217	1218	1218	1220	1220
1232	1253	1271	1271	1273	1273
1300	1300	1305	1323	1330	1330
1331	1333	1344	1346	1346	1350
1353	1361	1366	1368	1368	1375
1375	1378	1378	1402	1443	1444
1456	1456	1472	1477	1482	1501
1501	1507	1507	1531	1546	1546
1548	1548	1549	1552	1591	1602
1614	1631	1632	1633	1634	1635
1645	1646	1647	1647	1648	1662
1662	1663	1663	1667	1668	1683
3181	3181	3182	3182	3187	3187
3188	3188	3197	3197	3197	3197
3200	3200	3201	3201	3202	3202
3203	3203	3207	3207	3208	3208
3208	3208	3212	3212	3212	3212
3226	3226	3227	3227	3244	3245

Table S56. BBO–COF 3 planarity model calculations

Energy: –2511.96107540 hartrees

C	-1.14161	0.77757	-0.00022
C	0.07522	1.47055	-0.00020
C	1.18103	0.63875	-0.00020
C	1.14161	-0.77757	-0.00024
C	-0.07522	-1.47055	-0.00027
C	-1.18103	-0.63874	-0.00025
N	2.44330	-1.25694	-0.00023
C	3.19267	-0.19259	-0.00018
O	2.50382	1.00320	-0.00017
N	-2.44330	1.25695	-0.00020
C	-3.19267	0.19259	-0.00021

O	-2.50382	-1.00320	-0.00025
C	4.64792	-0.13082	-0.00014
C	-4.64792	0.13082	-0.00018
C	5.32714	1.09896	-0.00009
C	6.71595	1.12890	-0.00006
C	7.45682	-0.06334	-0.00006
C	6.77304	-1.29126	-0.00010
C	5.38637	-1.32727	-0.00014
C	-5.32714	-1.09896	-0.00017
C	-6.71595	-1.12890	-0.00016
C	-7.45682	0.06334	-0.00013
C	-6.77304	1.29126	-0.00012
C	-5.38637	1.32727	-0.00015
C	-8.93970	0.02784	-0.00011
C	8.93970	-0.02784	-0.00002
N	9.59309	-1.19896	0.00015
C	10.93348	-1.11919	0.00022
N	11.61789	0.03504	0.00011
C	10.87996	1.15586	-0.00008
N	9.53736	1.17290	-0.00013
N	-9.59309	1.19896	0.00009
C	-10.93348	1.11919	0.00019
N	-11.61789	-0.03504	0.00009
C	-10.87996	-1.15586	-0.00011
N	-9.53735	-1.17290	-0.00018
C	-11.59148	-2.45664	-0.00017
C	-11.70473	2.38530	0.00038
C	11.70473	-2.38531	0.00038
C	11.59148	2.45664	-0.00017
C	11.03999	-3.62185	0.00070
C	11.76606	-4.81008	0.00085
C	13.16279	-4.77910	0.00069
C	13.83114	-3.55221	0.00038
C	13.10842	-2.36196	0.00023
C	12.99473	2.49817	0.00012
C	13.66162	3.72059	0.00003
C	12.93724	4.91523	-0.00034
C	11.54055	4.88167	-0.00064
C	10.87026	3.66108	-0.00055
C	-11.03999	3.62185	0.00062
C	-11.76606	4.81008	0.00081
C	-13.16279	4.77910	0.00075
C	-13.83114	3.55221	0.00052
C	-13.10842	2.36196	0.00033

C	-10.87026	-3.66108	-0.00047
C	-11.54055	-4.88167	-0.00052
C	-12.93723	-4.91523	-0.00028
C	-13.66162	-3.72059	0.00001
C	-12.99473	-2.49817	0.00007
H	0.13418	2.55227	-0.00016
H	-0.13418	-2.55227	-0.00030
H	4.76200	2.02472	-0.00008
H	7.24517	2.07456	-0.00003
H	7.34718	-2.21036	-0.00010
H	4.85412	-2.27249	-0.00019
H	-4.76200	-2.02472	-0.00019
H	-7.24517	-2.07456	-0.00017
H	-7.34718	2.21036	-0.00011
H	-4.85412	2.27249	-0.00016
H	9.95614	-3.63422	0.00083
H	11.24228	-5.76211	0.00111
H	13.72816	-5.70734	0.00081
H	14.91738	-3.52364	0.00026
H	13.61641	-1.40440	-0.00001
H	13.54630	1.56506	0.00040
H	14.74802	3.74238	0.00026
H	13.45910	5.86861	-0.00041
H	10.97341	5.80854	-0.00094
H	9.78699	3.62308	-0.00078
H	-9.95614	3.63422	0.00067
H	-11.24229	5.76211	0.00100
H	-13.72816	5.70733	0.00090
H	-14.91738	3.52364	0.00048
H	-13.61641	1.40440	0.00015
H	-9.78699	-3.62308	-0.00066
H	-10.97340	-5.80854	-0.00076
H	-13.45910	-5.86861	-0.00033
H	-14.74802	-3.74238	0.00020
H	-13.54630	-1.56507	0.00030

Table S57. Vibrational frequencies for BBO–COF 3 planarity model calculations

6	7	13	18	23	28
29	29	31	34	41	53
57	58	60	64	73	77
93	102	108	108	110	141
173	180	184	197	211	216
216	222	231	242	250	266

272	281	299	310	325	349
365	376	384	414	415	416
416	417	417	417	427	442
443	449	457	457	479	482
484	525	528	534	547	556
612	612	632	632	633	634
646	647	660	661	675	676
684	685	689	689	706	707
715	715	715	718	718	721
727	738	747	763	763	796
796	809	816	816	823	860
860	860	862	862	863	864
866	866	867	868	868	869
869	888	891	891	937	946
957	957	959	959	984	984
986	986	990	990	1004	1004
1007	1007	1008	1008	1012	1012
1019	1019	1021	1021	1035	1035
1052	1052	1055	1055	1076	1079
1086	1091	1109	1109	1111	1111
1142	1143	1155	1172	1179	1179
1179	1179	1193	1193	1194	1194
1210	1210	1211	1211	1214	1214
1230	1230	1231	1254	1303	1325
1335	1335	1336	1336	1343	1346
1351	1362	1365	1366	1366	1368
1369	1401	1405	1406	1407	1407
1441	1441	1454	1457	1477	1492
1492	1496	1496	1533	1542	1542
1543	1545	1549	1551	1565	1566
1568	1569	1592	1611	1623	1635
1642	1642	1645	1645	1647	1662
1662	1662	1662	1668	1668	1683
3185	3185	3185	3185	3197	3197
3197	3197	3208	3208	3208	3209
3222	3222	3224	3224	3235	3235
3236	3236	3236	3236	3237	3237
3241	3241	3242	3242	3246	3247

References

1. M. Inbasekaran, R. Strom, *Org. Prep. Proced. Int.* 1991, **23**, 447-450.

2. J. F. Mike, A. J. Makowski, M. Jeffries-EL, *Org. Lett.* 2008, **10**, 4915-4918.

3. S. Jiang, J. Bacsa, X. Wu, J. Jones, R. Dawson, A. Trewin, D. J. Adams, A. I. Cooper, *Chem. Commun.* 2011, **47**, 8919-8921.

4. H. Tanaka, K. Shizu, H. Nakanotani, C. Adachi, *Chem. Mater.* 2013, **25**, 3766-3771.

5. A. K. Sekizkardes, S. Altarawneh, Z. Kahveci, T. Islamoglu, H. M. El-Kaderi, *Macromolecules.* 2014, **47**, 8328-8334.

6. D. A. Pyles, J. W. Crowe, L. A. Baldwin, P. L. McGrier, *ACS Macro Lett.* 2016, **5**, 1055-1058.

7. M. J. Frisch, G. W. Trucks, H. B. Schlegel, G. E. Scuseria, M. A. Robb, J. R. Cheeseman, G. Scalmani, V. Barone, G. A. Petersson, H. Nakatsuji, X. Li, M. Caricato, A. V. Marenich, J. Bloino, B. G. Janesko, R. Gomperts, B. Mennucci, Hratch, M., *Gaussian, Inc., Wallingford, CT,* 2016.

8. V. A. Rassolov, M. A. Ratner, J. A. Pople, P. C. Redfern, L. A. Curtiss, *J. Comput. Chem.* **2001**, *22* (9), 976.

9. A. D. J. Becke, *J. Chem. Phys.* **1993**, *98*, 5648.

10. C. Lee, W. Yang, R. G. Parr, *Phys. Rev. B.* **1988**, *37*, 785

11. A. E. Reed, R. B. Weinstock, F. J. Weinhold, *Chem. Phys.* **1985**, *831 (10), 1736*

12. E. Harder, W. Damm, J. Maple, C. Wu, M. Reboul, J. Y. Xiang, L. Wang, D. Lupyan, M. K. Dahlgren, J. L. Knight, J. W. Kaus, D. S. Cerutti, G. Krilov, W. L. Jorgensen, R. Abel, R. A. Friesner, *J. Chem. Theory Comput.* **2016**, *12*, 281.

13. *Schrödinger Release 2016-3: MacroModel.* Schrödinger LLC: New York, 2016.

14. T. Clark, J. Chandrasekhar, G. W. Spitznagel, P. V. R. Schleyer, *J. Comput. Chem.* **1983**, *4*, 294.
